# Estimating Multilevel Models on Data Streams

**DOI:** 10.1007/s11336-018-09656-z

**Published:** 2019-01-22

**Authors:** L. Ippel, M. C. Kaptein, J. K. Vermunt

**Affiliations:** 1 Institute of Data Science, Maastricht University, Maastricht, The Netherlands; 2 Tilburg University, Tilburg, The Netherlands

**Keywords:** Data streams, expectation maximization algorithm, multilevel models, machine (online) learning, SEMA, nested data

## Abstract

Social scientists are often faced with data that have a nested structure: pupils are nested within schools, employees are nested within companies, or repeated measurements are nested within individuals. Nested data are typically analyzed using multilevel models. However, when data sets are extremely large or when new data continuously augment the data set, estimating multilevel models can be challenging: the current algorithms used to fit multilevel models repeatedly revisit all data points and end up consuming much time and computer memory. This is especially troublesome when predictions are needed in real time and observations keep *streaming* in. We address this problem by introducing the Streaming Expectation Maximization Approximation (SEMA) algorithm for fitting multilevel models online (or “row-by-row”). In an extensive simulation study, we demonstrate the performance of SEMA compared to traditional methods of fitting multilevel models. Next, SEMA is used to analyze an empirical data stream. The accuracy of SEMA is competitive to current state-of-the-art methods while being orders of magnitude faster.

**Electronic supplementary material:**

The online version of this article (10.1007/s11336-018-09656-z) contains supplementary material, which is available to authorized users.

## Introduction

Novel technological advances—such as the widespread use of smartphone applications and the increased use of experience sampling methods—facilitate monitoring individuals over extensive periods of time (Barrett and Barrett, 2001; Beck, 2015; Buskirk and Andrus, 2012). When we monitor the behavior of customers on webpages, patients’ compliance with their medical regimen, or students’ performances, we are often interested in the behavior or traits of individuals. Based on individual-level estimates of traits, we can tailor actions or treatments; for example, we could recommend certain books tailored to individuals’ preferences as displayed by their browsing behavior (see, for example, Kaptein and Duplisnky, 2013). Such tailoring can only be carried out in real time when up-to-date predictions at the level of the individual are continuously available. In this paper, we present a computationally efficient algorithm for generating predictions of individuals’ traits in situations in which data are continuously collected.

When we continuously monitoring the attitudes and behaviors of individuals, data collection is effectively never finished: new customers continue to visit websites, patients continue to see their doctors, and students continue to enter and leave universities. This situation, in which new data enter continuously, is known as a *data stream* (Gaber, 2012; Gaber, Zaslavsky, and Krishnaswamy, 2005). Due to the continuous influx of new observations, data streams quickly result in (extremely) large data sets—possibly larger than would fit in computer memory. Even when the storage of all of these observations is technically feasible, obtaining up-to-date predictions using all available information is often computationally infeasible: the computational time to reestimate the necessary model parameters each time the data set is augmented often increases nonlinearly and quickly becomes unacceptable. In addition, the aforementioned examples all describe situations in which the collected data have a nested structure. This nesting introduces dependencies among the observations, and these dependencies in turn violate a key assumption of many statistical models that assume that observations are (conditionally) independent (Kenny and Judd, 1986). Nested structures are often dealt with using multilevel models (Goldstein and McDonald, 1988; Steenbergen and Jones, 2002) which, due to their complexity, only exaggerate the computation-time problems encountered when dealing with streaming data. Since the likelihood function of a multilevel model has to be maximized iteratively (using, for example, the expectation maximization algorithm [EM, Dempster, Laird, and Rubin, 1977)], the computation time increases exponentially. Thus, when real-time predictions of individuals’ scores are needed during a data stream, efficient computational methods designed to deal with data streams are needed.

In the literature, several adaptations of the EM algorithm that are computationally more efficient than the traditional EM algorithm have been proposed. For instance, Neal and Hinton (1998) provide analytic proof and justifications for a number of possible adaptations to the general EM algorithm to deal with large and/or growing data sets using batches of data. These adaptations are further explained and extended in McLachlan and Peel’s *Finite Mixture Models* book (2000, ch. 12) and by Thiesson, Meek, and Heckerman (2001). Wolfe, Haghighi, and Klein (2008) discuss how to parallelize the EM algorithm to deal with extremely large, but static, data sets. Finally, for a number of specific statistical models, computationally efficient versions of the EM algorithm have recently been proposed (Cappé and Moulines, 2009; Cappé 2011; Ippel, Kaptein, and Vermunt, 2016a; Liu, Almhana, Choulakian, and McGorman, 2006). The current paper adds to this literature by presenting a computationally efficient algorithm for the estimation of multilevel models—or “linear mixed models”—in data streams. While Ippel, Kaptein, and Vermunt, (2016a) already present an efficient algorithm for simple random intercept models, the current work nontrivially extends these results—most notably in the ‘E-step’—to allow for an arbitrary number of random effects and the covariances between these, and the inclusion of additional level 1 effects.

The SEMA (Streaming Expectation Maximization Approximation) algorithm can be categorized as an *online* learning algorithm. Online learning refers to “computing estimates of model parameters on-the-fly, without storing the data and by continuously updating the estimates as more observations become available” (Cappé, 2011). A simple illustration of online learning can be provided by inspecting the computation of a simple sample mean. The standard, *offline*, algorithm for computing a sample mean using,

$$\begin{aligned} \frac{1}{n}\sum _{t=1}^n x_t, \end{aligned}$$

is inefficient since whenever a new data point enters, we increase *n* by one, and we *redo* our computation by revisiting all our stored data points. As a result, all data have to be available in computer memory, and the computation time grows each time a new observation is added.

An *online* algorithm for computing the sample mean solves these issues. When computing the sample mean online, it is only necessary to store the sufficient statistics, *n* and $\bar{x}$, and *update* these when a new data point enters^1^:

1 $$\begin{aligned} \begin{aligned} n&\leftarrow n + 1\\ \bar{x}&\leftarrow \bar{x} + \frac{x_t-{\bar{x}}}{n}. \end{aligned} \end{aligned}$$

Here, *n* is the total number of observations, $\bar{x}$ is the sample mean, and ‘$\leftarrow$’ is the assignment operator, indicating that the left-hand side is replaced by what is on the right-hand side. Note that we will use this operator throughout the paper.

In the following section, the traditional, offline, estimation of multilevel models using the EM algorithm is explained in detail. Next, we illustrate the online fitting procedure of multilevel models using the SEMA algorithm we propose, and we discuss its computational gains. Subsequently, we present a simulation study examining the performance of SEMA in terms of estimation accuracy and prediction error. The simulation study is followed by an empirical example which highlights the challenges researchers encounter when analyzing data streams in practice. Finally, the results of both evaluations are discussed, and several directions for future research are highlighted.

Note that, in online supplementary material, additional results are presented. There, we present the remaining results of the simulation study and the application. Furthermore, in supplementary material, two illustrations are provided: the first illustration shows the effect of poor starting values on SEMA’s performances, while the second illustration shows the influence of the sequence in which data points arrive on SEMA’s parameter estimates.

## Offline Estimation of Multilevel Models

Let individual *j* have $i = 1, \dots , n_j$ observations, and let $n = \sum _{j=1}^J n_j$ be total number of observations collected from *J* individuals. The multilevel model can be denoted as:

2 $$\begin{aligned}&{y}_{ij}={\varvec{x}}_{ij}'{\varvec{\beta }} + {\varvec{z}}_{ij}'{\varvec{b}}_j+{\epsilon }_{ij},\nonumber \\&\quad {\varvec{b}}_j \sim {\mathcal {MVN}}(0 ,{\varvec{\Phi }})\nonumber \\&\quad {\epsilon }_{ij} \sim \mathcal {N}(0,\sigma ^2), \end{aligned}$$

where ${y}_{ij}$ is the response *i* of individual *j*, ${\varvec{x}}_{ij}$ is a $p \times 1$ vector of *fixed-*effect data, ${\varvec{z}}_{ij}$ is a $r\times 1$ vector of *random-*effect data, ${\varvec{\beta }}$ is a $p \times 1$ vector of fixed-effect coefficients, ${\varvec{b}}_j$ is a $r\times 1$ vector of random-effect coefficients, ${\varvec{\Phi }}$ is a $r\times r$ matrix with (co)variances of the random effects, ${\epsilon }_{ij}$ is the error term for each observation, and $\sigma ^2$ is the variance of the error term. The number of observations per individual, $n_j$, might differ across individuals. Furthermore, the variance of the random effects and the error variance are assumed to be independent: ${\varvec{\epsilon }} \perp {\varvec{b}}_j$.

Often, the maximum likelihood framework is used to estimate the parameters of the above multilevel model. If the random effects (${\varvec{b}}_j$) would have been observed, maximizing the log-likelihood

3 $$\begin{aligned} \begin{aligned} \ell ({\varvec{\beta }}, {\varvec{\Phi }}, \sigma ^2 |y, {\varvec{b}}_j) =&-\frac{n}{2}\ln \sigma ^2-\frac{1}{2} \sum _{j=1}^J \sum _{i=1}^{n_j} \Big ( \frac{(y_{ij}-{\varvec{x}}_{ij}'{\varvec{\beta }} - {\varvec{z }}_{ij}'{\varvec{b}}_j)}{\sigma } \Big ) ^2\\&-\frac{J}{2} \ln |{\varvec{\Phi }}| -\frac{1}{2}\sum _{j=1}^J {{\varvec{b}}'_j{\varvec{\Phi }}^{-1}{\varvec{b}}_j} \end{aligned} \end{aligned}$$

would be relatively straightforward. However, since the random effects are not directly observed (i.e., these are latent) we are confronted with a missing-data problem. The EM algorithm (Dempster, Laird, and Rubin, 1977) handles this missing-data problem by imputing the missing values with the expectations of ${\varvec{b}}_j$’s given the model parameters ${\varvec{\beta }}$, ${\varvec{\Phi }}$, and $\sigma ^2$ in the E-step, and subsequently maximizing the log-likelihood function given these expectations in the M-step.

### The Offline E-Step

When the missing ${\varvec{b}}_j$’s are imputed, there exist closed-form expressions to compute the model parameters. These expressions rely on a number of *complete-data sufficient statistics* (CDSS), which are computed as part of the E-step. Each of the model parameters, ${\varvec{\beta }}, {\varvec{\Phi }}$, and $\sigma ^2$, has its own CDSS which we refer to as ${\varvec{t}}_1$, ${\varvec{T}}_2,$ and $t_3$.

The CDSS for ${\varvec{\beta }}$ is defined as follows:

4 $$\begin{aligned} {\varvec{t}}_{1(k)} = \sum _{j=1}^J {\varvec{X}}'_j{\varvec{Z}}_j\hat{{\varvec{b}}}_{j(k)}, \end{aligned}$$

where ${\varvec{X}}_j$ is an $n_j \times p$ matrix, ${\varvec{Z}}_j$ is $n_j \times r$ matrix, *k* indexes the current iteration, ${\varvec{t}}_{1(k)}$ is an $p \times 1$ vector, and $\hat{{\varvec{b}}}_{j(k)}$ is given by:

5 $$\begin{aligned} \begin{aligned} \hat{{\varvec{b}}}_{j(k)} =&{\varvec{C}}_{j(k)}^{-1}\left( {\varvec{Z}}'_j{\varvec{y}}_j-{\varvec{Z}}'_j{\varvec{X}}_j\hat{{\varvec{\beta }}}_{(k-1)}\right) . \end{aligned} \end{aligned}$$

Here ${\varvec{C}}_{j(k)}$ quantifies the uncertainty of the imputations of ${\varvec{b}}_j$’s, and the subscript $k-1$ indicates that $\hat{{\varvec{\beta }}}$ of the previous iteration is used in the computation.^2^${\varvec{C}}_{j(k)}$ itself is an $r \times r$ matrix given by:

6 $$\begin{aligned} {\varvec{C}}_{j(k)} = {\varvec{Z}}'_j{\varvec{Z}}_j+ \hat{\sigma }_{(k-1)}^2 \hat{{\varvec{\Phi }}}_{(k-1)}^{-1}. \end{aligned}$$

The CDSS for the variance of the random effect, ${\varvec{T}}_{2(k)}$, is given by:

7 $$\begin{aligned} {\varvec{T}}_{2(k)}= \sum _{j=1}^J \hat{{\varvec{b}}}_{j(k)}\hat{{\varvec{b}}}_{j(k)}'+ \hat{\sigma }_{(k-1)}^2 \sum _{j=1}^J {\varvec{C}}_{j(k)}^{-1}, \end{aligned}$$

where ${\varvec{T}}_{2(k)}$ is an $r \times r$ matrix. In words, ${\varvec{T}}_{2(k)}$ is the sum of the squared random-effect coefficients plus the additional uncertainty due to the fact that ${\varvec{b}}_{j(k)}$ is not observed.

Lastly, the CDSS of the residual variance, $\sigma ^2_{(k)}$, $t_{3(k)}$ is given by:

8 $$\begin{aligned} t_{3(k)} = \sum _{j=1}^J u'u + \hat{\sigma }_{(k-1)}^2tr\left( \sum _{j=1}^J{\varvec{C}}^{-1}_{j(k)} {\varvec{Z}}_j'{\varvec{Z}}_j\right) . \end{aligned}$$

where $u = {\varvec{y}}_j-{\varvec{X}}_{j}\hat{{\varvec{\beta }}}_{(k-1)} - {\varvec{Z}}_{j}\hat{{\varvec{b}}}_{j(k)}$, is the residual.

### The Offline M-Step

In the M-step, the log-likelihood function is maximized, given the CDSS computed in the E-step. In iteration *k*, the coefficients of the fixed effects, ${\varvec{\beta }}$, are computed using the normal equations:

9 $$\begin{aligned} \begin{aligned} \hat{{\varvec{\beta }}}_{(k)} =&\left( \sum _{j=1}^J {\varvec{X}}'_j {\varvec{X}}_j \right) ^{-1} \sum _{j=1}^J {\varvec{X}}'_j{\varvec{y}}_j-{\varvec{t}}_{1(k)}. \end{aligned} \end{aligned}$$

The variance of the random effects (${\varvec{\Phi }}_{(k)}$) is computed by dividing ${\varvec{T}}_{2(k)}$ by the number of individuals:

10 $$\begin{aligned} \begin{aligned} \hat{{\varvec{\Phi }}}_{(k)}&= \frac{{\varvec{T}}_{2(k)}}{J}. \end{aligned} \end{aligned}$$

Lastly, the residual variance ($\sigma ^2_{(k)}$) is given by:

11 $$\begin{aligned} \begin{aligned} \hat{\sigma }_{(k)}^2&=\frac{t_{3(k)}}{n} \end{aligned} \end{aligned}$$

## Online Estimation of Multilevel Models

Here, we introduce the Streaming Expectation Maximization Approximation (SEMA) algorithm. At the end of this section, the full algorithm (see Algorithm 1) is described.

### The Online E-step

Previously, we used subscript *k* to indicate the iterations of the EM algorithm. In this section, we drop this subscript to emphasize that unlike the EM algorithm, the SEMA algorithm only updates the CDSS using a single data point, without revisiting previous data points. Note that the term data point refers to a vector which includes an identifier for an individual, the covariates with fixed effects and random effects, and the observation of the dependent variable. When a data point enters, the SEMA algorithm performs an E-step only for the individual that belongs to the data point that recently entered. After the E-step for this individual, all three model parameters are updated in the M-step. Due to this updating scheme, SEMA updates the parameter estimates when a new data point enters, instead of fitting the model anew.

Two aspects of Eq. 4 ($\mathbf {t}_1$) are challenging in the context of a data stream. First, the CDSS for $\hat{{\varvec{\beta }}}$ consists of a summation over *J* individuals. If the (weighted) contribution of a new data point would simply be added, then this would result in including the data from the same individual repeatedly. Second, to compute ${\varvec{t}}_1$ we need $\hat{{\varvec{b}}}_j$ which depends on the model parameters. Because the model parameters are updated each time a new data point enters, obtaining the exact same result using either the online or offline computation of this CDSS would imply that all contributions to ${\varvec{t}}_1$ need to be recomputed for each data point. This is not feasible. Therefore, we resort to an approximate solution. Note that this approximation improves as the number of observations per individual grows.

The solution we chose is as follows: when a new data point enters, the contribution of the individual belonging to this data point is subtracted from ${\varvec{t}}_1$ to account for the fact that this individual has already contributed to ${\varvec{t}}_1$. Next, $\hat{{\varvec{b}}}_j$ of this individual is recomputed, such that the new contribution to ${\varvec{t}}_1$ of this individual can be added. Because the online implementation of the CDSS is not exactly the same as the offline CDSS, we refer to the online computed CDSS of the fixed effects as ${\tilde{{\varvec{t}}}}_1$. The contribution to ${\tilde{{\varvec{t}}}}_1$ resulting from a single individual can be computed using:

12 $$\begin{aligned} {\tilde{{\varvec{t}}}}_{1(t)} \leftarrow {\tilde{{\varvec{t}}}}_{1(t-1)}-{\varvec{t}}_{1j_{t(t-1)}} + {\varvec{t}}_{1j_{t(t)}}, \end{aligned}$$

where ${\varvec{t}}_{1j_{t(t-1)}}$ represents the previous contribution of individual $j_t$, which is the individual associated with the most recent data point.

For the CDSS, we use subscript *t* to indicate that the CDSS is obtained by subtracting the previous contribution of individual $j_t$ after which the new contribution is added. The computation of ${\varvec{t}}_{1j}$ is given by

13 $$\begin{aligned} {\varvec{t}}_{1j}= {\varvec{X}}_j'{\varvec{Z}}_j\hat{{\varvec{b}}}_j, \end{aligned}$$

where the ${\varvec{X}}_j'{\varvec{Z}}_j$ matrix can be updated online:

14 $$\begin{aligned} {\varvec{X}}'_{j}{\varvec{Z}}_{j} \leftarrow {\varvec{X}}'_{j}{\varvec{Z}}_{j}+ {\varvec{x}}_{ij} {\varvec{z}}'_{ij}. \end{aligned}$$

Here, ${\varvec{X}}'_{j}{\varvec{Z}}_{j}$ is only updated for the individual associated with the most recent data point, and ${\varvec{x}}_{ij}$ and ${\varvec{z}}'_{ij}$ are the new values of fixed effects and random covariates of this individual. Unlike Eq. 12, Eq. 14 is exact. Using Eq. 14, none of the data points themselves (${\varvec{x}}_{ij}$ and ${\varvec{z}}_{ij}$) need to be stored since only the results of the matrix multiplication are stored. When new data present themselves, the outer product of ${\varvec{x}}_{ij} {\varvec{z}}_{ij}'$ is merely added to the current result.

The coefficients of the random effects (Eq. 5: $\hat{{\varvec{b}}}_j={\varvec{C}}_j^{-1}({\varvec{Z}}'_j{\varvec{y}}_j-{\varvec{Z}}'_j{\varvec{X}}_j\hat{{\varvec{\beta }}})$) can similarly be approximated online. We first detail how ${\varvec{C}}_j$ (Eq. 6) is computed online. The computation of ${\varvec{C}}_j$ uses a matrix product ${\varvec{Z}}'_j{\varvec{Z}}_j$. When new data enter, this matrix product can be updated online as follows:

15 $$\begin{aligned} {\varvec{Z}}'_j{\varvec{Z}}_j \leftarrow {\varvec{Z}}'_j{\varvec{Z}}_j + {\varvec{z}}_{ij} {\varvec{z}}_{ij}', \end{aligned}$$

which is similar to Eq. 14. The ${\varvec{Z}}'_j{\varvec{Z}}_j$ matrix needs to be stored per individual. The online computation of ${\varvec{C}}_j$ is given by:

16 $$\begin{aligned} {\varvec{C}}_j = {\varvec{Z}}'_j{\varvec{Z}}_j + \hat{\sigma }^2\hat{{\varvec{\Phi }}}^{-1}. \end{aligned}$$

Using the online formulation of ${\varvec{C}}_j$, the next step to compute $\hat{{\varvec{b}}}_j$ is given by:

17 $$\begin{aligned} {\varvec{z}}_jy_j \leftarrow {\varvec{z}}_jy_j+ {\varvec{z}}_{ij} y_{ij}, \end{aligned}$$

where ${\varvec{z}}_jy_j$ is an $r \times 1$ vector. Note that the matrix multiplication ${\varvec{Z}}'_j{\varvec{X}}_j$ (see Eq. 5) is equal to the transpose of the matrix ${\varvec{X}}_j'{\varvec{Z}}_j$ in Eq. 14. The online computation of $\hat{{\varvec{b}}}_j$ is:

18 $$\begin{aligned} \hat{{\varvec{b}}}_j={\varvec{C}}_j^{-1}({\varvec{z}}_jy_j-({\varvec{X}}'{\varvec{Z}}_j)' \hat{{\varvec{\beta }}}) \end{aligned}$$

Similar to the computation of ${\tilde{{\varvec{t}}}}_1$, ${\tilde{{\varvec{T}}}}_2$ is also a summation over individuals (Eq. 7: ${\varvec{T}}_{2}= \sum _{j=1}^J \hat{{\varvec{b}}}_{j}\hat{{\varvec{b}}}_{j}'+ \hat{\sigma }^2 \sum _{j=1}^J {\varvec{C}}_{j}^{-1}$). Therefore, a similar update regime is used for this CDSS:

19 $$\begin{aligned} \tilde{{\varvec{T}}}_{2(t)} \leftarrow \tilde{{\varvec{T}}}_{2(t-1)}-{\varvec{T}}_{2j_t(t-1)} + {\varvec{T}}_{2j_t(t)}, \end{aligned}$$

where

20 $$\begin{aligned} {\varvec{T}}_{2j} = \hat{{\varvec{b}}}_j\hat{{\varvec{b}}}_j'+ \hat{\sigma }^2 {\varvec{C}}_j^{-1}. \end{aligned}$$

In order to update $\tilde{{\varvec{T}}}_2$ online, the previous contribution of this individual is again subtracted before the new contribution is computed and added.

Finally, the online computation of $t_3$ is presented (Eq. 8). The computation of $t_3$ is unlike the previous two CDSS, a summation over *n* data points. Therefore, we first rewrite the contribution of each single data point, as a contribution of an individual to the $\tilde{t}_3$:

21 $$\begin{aligned} \begin{aligned} t_{3j}&= y_j'y_j + \hat{{\varvec{\beta }}}'{\varvec{X}}'_j{\varvec{X}}_j\hat{{\varvec{\beta }}}+\hat{{\varvec{b}}}'_j {\varvec{Z}}'_j{\varvec{Z}}_j\hat{{\varvec{b}}}_j - 2y'_j{\varvec{X}}_j\hat{{\varvec{\beta }}} - 2y'_j{\varvec{Z}}_j\hat{{\varvec{b}}}_j\\&\quad + 2\hat{{\varvec{\beta }}}'{\varvec{X}}'_j{\varvec{Z}}_j\hat{{\varvec{b}}}_j+ \hat{\sigma }^2 tr({\varvec{C}}_j^{-1}) \end{aligned} \end{aligned}$$

where $y_j'y_j$ is computed as the sum of the squared observations of the dependent variable: $\sum _{i=1}^{n_j} y_{ij}^2$, and where the computation of ${\varvec{X}}'_j{\varvec{X}}_j$ is similar to that of ${\varvec{Z}}'_j{\varvec{Z}}_j$. Using Eq. 21, $\tilde{t}_3$ can be updated similarly to the other CDSS:

22 $$\begin{aligned} \tilde{t}_{3(t)} \leftarrow \tilde{t}_{3(t-1)}-t_{3j_t(t-1)} + t_{3j_t(t)}, \end{aligned}$$

Equation 21 is a reformulation of the estimation of $\tilde{t}_{3(t)}$, compared to what was presented in Ippel, Kaptein, and Vermunt (2016a). In the 2016 paper, $\tilde{t}_{3(t)}$ is computed using averages as summary statistics. That implementation, however, cannot be used when one chooses to model level 1 effects (fixed effects and random slopes). Using the current implementation, level 1 effects can be included in the model. The online implementation of the E-step presented here makes it possible to drop the historical data points and only store summaries of the data points (see for exact details Algorithm 1 below).

### The Online M-Step

The online implementation of the M-step of both the variance of the random effects, $\hat{{\varvec{\Phi }}}= \frac{{\tilde{{\varvec{T}}}}_2}{J}$, and the residual variance, $\hat{\sigma }^2= \frac{{\tilde{{t}}}_3}{n}$, is the same as the offline implementation discussed above. This, however, does not hold for the online computation of $\hat{{\varvec{\beta }}}= ( \sum _{j=1}^J {\varvec{X}}'_j {\varvec{X}}_j \big )^{-1} \sum _{j=1}^J {\varvec{X}}'_j{\varvec{y}}_j-\tilde{{\varvec{t}}}_{1}$, which we detail in this section.

The first element of Eq. 9 is the $\sum _{j=1}^J {\varvec{X}}'_j{\varvec{X}}_j$ matrix. This matrix can be updated online using the same update regime as already presented in Eq. 14:

23 $$\begin{aligned} {\varvec{X}}'{\varvec{X}} \leftarrow {\varvec{X}}'{\varvec{X}} + {\varvec{x}}_{ij} {\varvec{x}}_{ij}'. \end{aligned}$$

However, in order to subsequently compute $\hat{{\varvec{\beta }}}$, the inverse of ${\varvec{X}}'{\varvec{X}}$ is needed. Computing the inverse of a matrix can be a costly procedure if the number of covariates is large. A solution is to directly update the inverted matrix using the Sherman–Morrison formula (Escobar and Moser, 1993; Plackett, 1950; Sherman and Morrison, 1950):

24 $$\begin{aligned} ({\varvec{X}}'{\varvec{X}})^{-1}\leftarrow ({\varvec{X}}'{\varvec{X}})^{-1} - \frac{({\varvec{X}}'{\varvec{X}})^{-1}{\varvec{x}}_{ij}{\varvec{x}}_{ij}'({\varvec{X}}'{\varvec{X}})^{-1}}{1+{\varvec{x}}_{ij}'({\varvec{X}}'{\varvec{X}})^{-1}{\varvec{x}}_{ij}}. \end{aligned}$$

Using this formulation, ${\varvec{X}}'{\varvec{X}}$ only has to be inverted once, after which the inverted matrix is directly updated with the new data. In practice, this means that one has to wait until enough data have entered, such that ${\varvec{X}}'{\varvec{X}}$ is invertible.

The second part of Eq. 9 is the multiplication of the covariates with the dependent variable. This can be updated online as follows:

25 $$\begin{aligned} {\varvec{x}}y\leftarrow {\varvec{x}}y + {\varvec{x}}_{ij} y_{ij}, \end{aligned}$$

where ${\varvec{x}}y$ is a $p \times 1$ vector. Inserting the online computed components of Eq. 9 into the equation results in the computation of $\hat{{\varvec{\beta }}}$:

26 $$\begin{aligned} \hat{{\varvec{\beta }}} = ({\varvec{X}}'{\varvec{X}})^{-1}({\varvec{x}}y-\tilde{{\varvec{t}}}_1) \end{aligned}$$

We present an overview of the SEMA algorithm, assuming that ${\varvec{X}}'{\varvec{X}}$ is already inverted, in Algorithm 1. The first line indicates which elements the algorithm uses, where $\theta$ denotes the elements which are available at the global level, whereas $\theta _j$ contains all the elements which are stored for each individual. Only $\theta _j$ for the individual that belongs to the most recent data point is used in the update step; the remaining $\theta _j$’s do not have to be available in memory. The standard EM algorithm would use all data, from each individual, to fit the model. Thus, while the memory usage of the EM algorithm grows as a function of *n*, SEMA’s memory usage only grows with *J*. An implementation of the SEMA algorithm in [R] (R core Team, 2018) can be found at https://github.com/L-Ippel/SEMA.
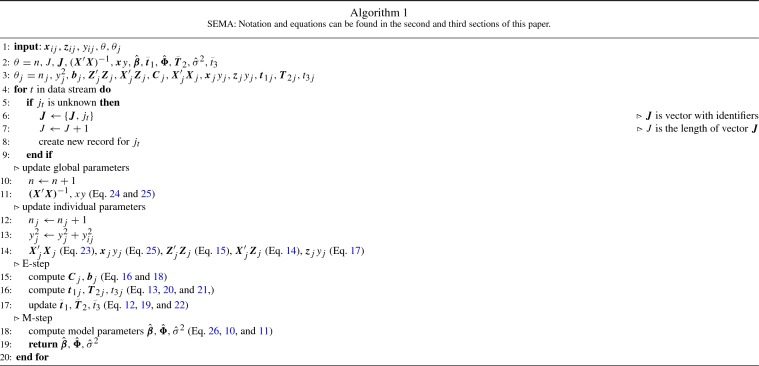


## Computational Complexity

We have motivated the SEMA algorithm described above by focusing on its computational gains. While below we strengthen this argument by presenting the running times of both EM and SEMA in our simulation studies, we first focus theoretically on the computational gains attained by SEMA. Evaluating the exact computational complexity of the EM algorithm is not straightforward. First, the complexity is dependent on the stopping criterion of the algorithm (maximum number of iterations versus some convergence rule); second, the implementation (using the formulations which are sums over data points versus sums over individuals) influences the computational complexity. Third, the context in which the complexity is evaluated matters: the number of individuals in the data compared to the number of observations within individuals influences the number of computations needed. Finally, it makes a large difference whether all the observations are assumed to be already available in memory or whether new observations streaming in. Because of these difficulties, we do not provide exact bounds, but rather we provide an intuition regarding the computational gains of switching from an offline (EM) algorithm to an online (SEMA) algorithm.

To illustrate the computational gains of SEMA, let us revisit the computation of a simple sample mean—as discussed in the introduction—either offline or online. Using an offline procedure, each time a new data point enters, the entire procedure needs to be redone: we need to recount the number of data points and we need to recompute a sum over all the data points. Ignoring the details of the exact computation, this process thus executes one set of computations for $n=1$, two sets of computations for $n=2$, etc. Hence, the number of computations involved scales by

$$\begin{aligned} \begin{aligned} 1+2+3+\dots +n&=\frac{1}{2}n(n+1),\\&=O(n^2). \end{aligned} \end{aligned}$$

as a function of *n*. It is well known that when data keep entering at a rapid pace even rather simple computations become infeasible when the number of computations scales quadratically with *n* (ch. 3, Cormen, Leiserson, Rivest and Stein, 2009). Using an online algorithm instead, the mean can be directly updated as shown in Eq. 1. The computational complexity of this online algorithm to compute the sample mean is equal to

$$\begin{aligned} \begin{aligned} 1+1+1+\dots +1&=n,\\&=O(n), \end{aligned} \end{aligned}$$

and is thus linear in *n*.

**Fig. 1:**
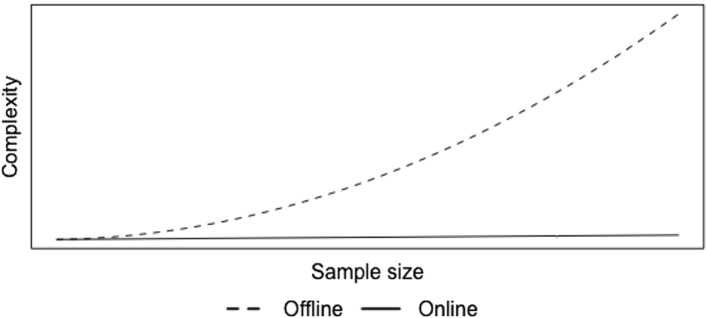
Computational complexity of online versus offline algorithms to compute the sample mean.

In Fig. 1, the differences in computational complexity between the offline and online computations of the sample mean are illustrated. For the EM algorithm versus the SEMA algorithm, this difference is magnified. While SEMA is still *O*(*n*), the offline EM algorithm *repeatedly* revisits all the data to reestimate the multilevel model and thus the number of computations grows even faster than $O(n^2)$. This is illustrated most easily by comparing specific parts of the EM and the SEMA algorithm; we highlight two differences that directly influence the computation times:

1. The computation of $(\mathbf {X}'\mathbf {X})^{-1}$: while the EM algorithm recomputes the $\mathbf {X}'\mathbf {X}$ matrix each time a new data point arrives and subsequently and computes the inverse of this matrix, the SEMA algorithm directly updates the inverted matrix. Inverting the matrix can be especially costly when there are a large number of covariates. Thus, SEMA beats EM by both not revisiting historical data, and not requiring repeated matrix inversions.
2. The computation of the CDSS: using the traditional formulation of the EM algorithm, all contributions to the CDSS for all individuals are reestimated when new data enter, and this process is repeated multiple times; it is repeated for as many iterations as necessary to allow the EM algorithm to converge. On the other hand, the SEMA algorithm only recomputes the contributions to these CDSS for one single individual, and does so only once.

Note that some of these improvements do come at a cost: because CDSS contributions associated with individuals that do not reoccur in the data stream are not updated, their estimates become outdated. Especially when individuals do not return repeatedly, these outdated contributions could bias the resulting estimates. Regular updates—or “sweeps”—through the individual-level estimates that recompute the CDSS contributions for all individuals at given intervals could decrease this bias. This idea is already introduced in Ippel, Kaptein, and Vermunt (2016a) and referred to as “SEMA Update”.

## Simulation Study

### Design

Our simulation study is directly inspired by the application presented in Kooreman and Scherpenzeel (2014). In this study, the authors use a random intercept model with level 1 and level 2 predictors to analyze their longitudinal data regarding fluctuations in people’s weight (for more details, see “SEMA in action” below). By carefully extending the model used by Kooreman and Scherpenzeel (2014), we examine the influence of two important factors on the performance of SEMA.

First, we examine the changes in the number of random effects. Varying the number of random effects influences the reliability of the random coefficients, because the information of the data is spread out over more latent variables. Accordingly, the settings with more random effects are expected to be more difficult for SEMA to fit the multilevel model, i.e., SEMA will need to process more data before the parameter estimates are close to the ML values. Second, we examine variations in the associations between the random effects; this factor is also well known to affect the performance of the EM algorithm. Increasing the strength of the associations between the random effects, i.e., introducing collinearity, makes it more difficult to estimate the coefficients.

In our simulations, we examine a total of 4 conditions. Inspired by the application presented in Kooreman and Scherpenzeel (2014), all four conditions consider 15 fixed effects: 5 continuous and one categorical variable with 4 categories (i.e., 3 dummy variables) at level 1, and 3 continuous variables and 2 categorical variables, one consisting of 2 categories (to represent gender) and one variable with 3 categories (education level) at level 2. Next, the four conditions are as follows:

- Condition A: a simple random intercept model (a model containing a single variance component),
- Conditions B, C, and D: a random intercepts *and* slopes model with weak (B), medium (C), and strong (D) associations between these random effects. Note that in these three conditions we have a total of 5 variance components, one for intercept and four “slopes” for four of the five level 1 fixed effects.

Inspired by the application, the (true) parameter values used to generate the data are:

- Fixed effects: 100.0, 0.1, 0.5, 0.9, 1.3, 1.7, 2.1, 2.5, 2.9, 3.3, 3.7, 4.1, 4.5, 4.9, and 5.3;
- Variance random effects: 50 (of intercept $=100.0$; condition A), 0.2, 0.6, 1.8, and 5.0 (of, respectively, 0.1, 0.5, 0.9, and 1.3; conditions B–D);
- Correlations between the random effects: 0 (condition B), .15 (condition C) or .5 (condition D);
- Residual variance: 5.0 (all conditions);

The generated data streams consist of $n=50{,}000$ observations, and the number of individuals was equal to 1000. The data were generated as follows: first the level-2 observations were generated, both fixed-effect data and the random-effect coefficients including the (co)variances. The coefficients of the random effects as well as the level-2 data were drawn from multivariate normal distributions. Then, using these 1000 individuals, 50,000 samples were drawn at random, resulting in a data stream where the observations from each individual are spread out over the entire data stream. In expectation, each individual has $n_j = 50$ observations. Note that additional studies are presented in online supplementary material.

### Procedure

At the start of the analysis of the data stream, we used a training set of $n=2000$. While the EM and SEMA algorithms require some data to ensure that the $\mathbf {X}'\mathbf {X}$ is invertible, this training set is mainly used to ensure that the start values are chosen well. When these start values are far from the ML values, the EM algorithm requires many iterations to converge. For the SEMA algorithm, this issue is even more pronounced as the CDSS are only updated one individual at a time. Since this study is not concerned with how many iterations the EM algorithm requires to converge, the start values for the EM algorithm are those values the data were generated with. The EM algorithm was run until convergence with a maximum of 800 iterations, where convergence is defined by parameter values changing less than 0.0001 from one iteration to the next. The obtained values were subsequently used as start values for the SEMA algorithm.

Besides the SEMA algorithm as introduced in this paper, we also implemented SEMA Update (SU, Ippel, Kaptein, and Vermunt 2016a). In this algorithm, at set times, the estimates for each of the *J* individuals are updated by performing a “sweep” through all the currently stored estimates. In our study, we set the additional update regime to every $n=1000$ data points. This update is useful in situations where individuals do not return often (or drop out), since the update allows their outdated contributions to be revised. Note that this update only uses the statistics which are aggregated at the individual level, and it therefore does not revisit older data points.

We compare these two implementations of the SEMA algorithm with two implementations of the EM algorithm. The first implementation uses all data. To keep the simulation study within an acceptable running time, the EM algorithm was set to update the parameter estimates in (incremental) batches of $n=1000$ data points. The maximum number of iterations was set to 20, and the start values were those estimates obtained in the previous batch. At the end of the stream, the EM algorithm was run until convergence. The second EM implementation was inspired by an approach commonly used in data streams: a sliding window (Gaber, Zaslavsky, and Krishnaswamy, 2005). A sliding window is an efficient tool to make sure that the analyses will not take increasingly more time or computer memory by fixing the amount of data taken into account. Whenever new data enter the data set, the oldest data are forgotten. Thus, the data under consideration, i.e., the “Window”, only consist of the *m* most recent data points. In our study, the sliding window EM implementation (SWEM) used a window of $m = 10{,}000$ data points. Similar to the EM implementation, the SWEM implementation was set to update only every 1000 data points. During the simulation study, we monitored two aspects of the estimation procedures. First, we monitored the accuracy of the parameter estimates of SEMA compared to the EM implementations. Second, we examined the prediction accuracy of the different procedures (where for new individuals the first prediction was generated by setting the random effects ($\mathbf {b}_j$) equal to zero). All conditions were replicated 1000 times.

### Results

In Table 1, the estimated fixed effects and their standard errors across conditions are presented. The results are shown at two points during the stream, $n=25{,}000$ and $n=50{,}000$. Only two coefficients are presented, though the remaining coefficients have similar results, see online supplementary material. The results of SEMA are very similar to the results of the EM algorithm, although the variance over the simulation runs is larger for SEMA compared to EM and SEMA Update (SU): the additional updates of SU result in smaller variances. The standard errors are very similar: they deviate with (less than) .002 across methods. The results of SWEM vary slightly more than the EM due to the fact that this method only uses the $n=10{,}000$ most recent data points. In Table 2, the estimates of random effects are presented at two points during the data stream. All methods show a slight underestimation of the random intercept which can be expected from ML estimates, though all methods do retrieve the data-generating values of the random slopes, which are smaller in value. The estimates of the random intercept vary more than the estimates of the random slopes, independent of the estimation method. Most likely, this is a result of the ML framework and not of the estimation methods. To improve these results, the restricted ML (REML) framework could be used. In the REML framework, corrected variance terms are used for the estimation, controlling for the loss of the degrees of freedom for the estimation of the coefficients of the fixed effects (Harville, 1977).

**Fig. 2:**
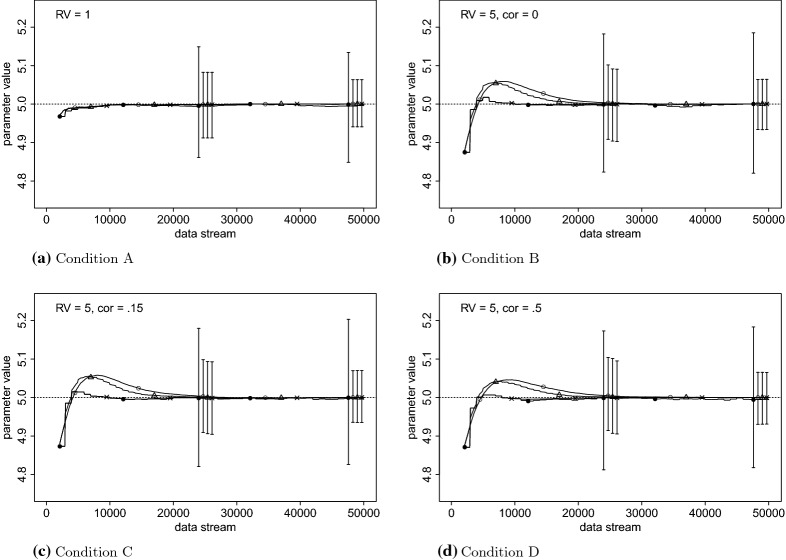
Estimated residual variance, the true value is 5. The error bars indicate the 95% empirical interval of the 1000 simulation runs. The ‘$\times$’ is EM, triangle is SEMA Update, open circle is SEMA, and closed circle is Sliding Window EM.

**Fig. 3:**
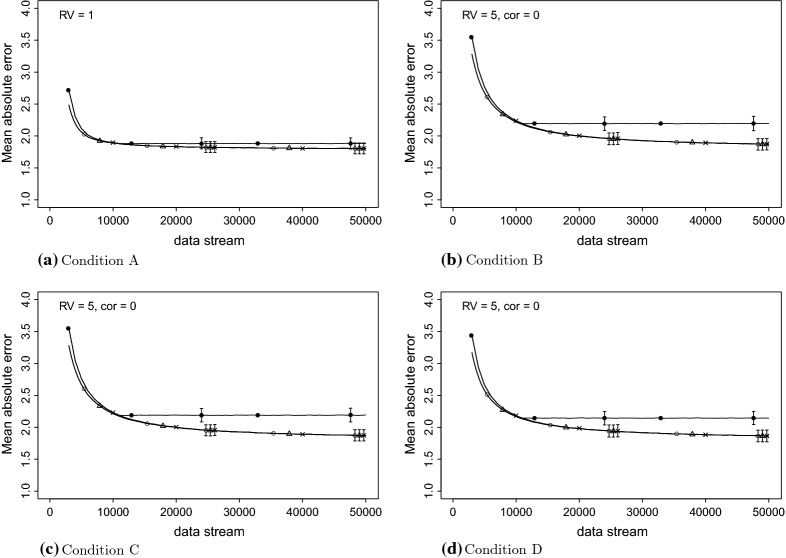
Estimated residual variance, the true value is 5. The error bars indicate the 95% empirical interval of the 1000 simulation runs. The ‘$\times$’ is EM, triangle is SEMA Update, open circle is SEMA, and closed circle is Sliding Window EM.

Table 3 contains the mean absolute error (MAE), the root-mean-squared error (RMSE), and the 95% empirical confidence interval at the end of the data stream for the fixed effects, the variance of the random effects, and the residual variance. The presented results in this table are from the same fixed effects and random effects presented in Tables 1 and 2. The data-generating values of the presented parameters are $\beta = 100$ and .1; $\phi ^2 = 50$ and .2; and $\sigma ^2 = 5$. The residual variance and the mean absolute prediction error are also presented in Figs. 2 and 3. While EM generally slightly outperforms SEMA, in terms of MAE and RMSE, the confidence intervals are very similar and the differences are small.

Lastly, in Table 4, we present the mean absolute error and root-mean-squared error with 95% empirical confidence intervals over *all n* predictions. The method with the lowest mean absolute error and root-mean-squared error is SEMA Update (SU), followed by SEMA; however, all four methods are very similar.

**Table 1:** Average results of the estimates of two of the 15 fixed effects over 1000 simulation runs.

Condition	$n_{\times 1000}$	SEMA			SU			EM			SWEM		
		$\hat{\beta }$	$s^2$	se$^\mathrm{a}$	$\hat{\beta }$	$s^2$	se$^\mathrm{a}$	$\hat{\beta }$	$s^2$	se$^\mathrm{a}$	$\hat{\beta }$	$s^2$	se$^\mathrm{a}$
rv = 1	25	100.002	0.239	0.015	100.003	0.232	0.015	100.007	0.204	0.014	100.007	0.204	0.014
	50	100.001	0.227	0.015	100.002	0.216	0.015	100.005	0.198	0.014	100.007	0.196	0.014
	25	0.098	0.001	0.001	0.098	0.001	0.001	0.098	0.001	0.001	0.098	0.001	0.001
	50	0.100	0.000	0.000	0.100	0.000	0.000	0.100	0.000	0.000	0.100	0.001	0.001
rv = 5, cor = 0	25	99.994	0.262	0.016	99.993	0.252	0.016	99.991	0.210	0.014	99.991	0.210	0.014
	50	99.992	0.242	0.016	99.991	0.226	0.015	99.986	0.199	0.014	99.986	0.202	0.014
	25	0.099	0.001	0.001	0.099	0.001	0.001	0.099	0.001	0.001	0.099	0.001	0.001
	50	0.100	0.000	0.001	0.100	0.000	0.001	0.100	0.000	0.001	0.101	0.001	0.001
rv = 5, cor = .15	25	99.971	0.239	0.016	99.972	0.230	0.015	99.972	0.194	0.014	99.972	0.194	0.014
	50	99.970	0.222	0.015	99.970	0.207	0.014	99.970	0.185	0.014	99.972	0.186	0.014
	25	0.101	0.001	0.001	0.101	0.001	0.001	0.101	0.001	0.001	0.101	0.001	0.001
	50	0.101	0.000	0.001	0.101	0.000	0.001	0.101	0.000	0.001	0.100	0.001	0.001
rv = 5, cor = .5	25	99.997	0.212	0.015	99.997	0.199	0.014	99.999	0.156	0.012	99.999	0.156	0.012
	50	99.997	0.187	0.014	99.997	0.167	0.013	99.992	0.141	0.012	99.999	0.149	0.012
	25	0.102	0.001	0.001	0.102	0.001	0.001	0.101	0.001	0.001	0.101	0.001	0.001
	50	0.100	0.000	0.001	0.100	0.000	0.001	0.100	0.000	0.001	0.099	0.001	0.001

Data-generating values were: $\beta =100$ and $\beta = .1$.

$^\mathrm{a}$se = $\frac{\sqrt{\frac{1}{S-1}\sum _s(\hat{\beta }_s - \beta )^2}}{\sqrt{S}}$, where *S* is the total number of simulation runs.

**Table 2:** Average results of the estimates of the variance of one (condition A) or two (conditions B–D) of the 5 random effects over 1000 simulation runs.

Condition	$n_{\times 1000}$	SEMA			SU			EM			SWEM		
		$\hat{\phi }^2$	$s^2$	se$^\mathrm{a}$	$\hat{\phi }^2$	$s^2$	se$^\mathrm{a}$	$\hat{\phi }^2$	$s^2$	se$^\mathrm{a}$	$\hat{\phi }^2$	$s^2$	se$^\mathrm{a}$
rv = 1	25	49.756	5.305	0.073	49.744	5.302	0.073	49.702	5.285	0.073	49.702	5.285	0.073
	50	49.730	5.157	0.072	49.712	5.152	0.072	49.687	5.139	0.072	49.690	5.209	0.073
rv = 5, cor = 0	25	49.631	5.068	0.072	49.622	5.067	0.072	49.568	5.054	0.072	49.568	5.054	0.072
	50	49.626	4.944	0.071	49.603	4.942	0.071	49.569	4.932	0.072	49.569	5.117	0.073
	25	0.220	0.007	0.031	0.202	0.004	0.032	0.194	0.002	0.032	0.194	0.002	0.032
	50	0.199	0.000	0.032	0.199	0.000	0.032	0.199	0.000	0.032	0.199	0.002	0.032
rv = 5, cor = .15	25	49.703	4.776	0.070	49.693	4.773	0.070	49.644	4.765	0.070	49.644	4.765	0.070
	50	49.715	4.623	0.069	49.693	4.622	0.069	49.669	4.621	0.069	49.656	4.724	0.070
	25	0.228	0.007	0.031	0.209	0.004	0.031	0.194	0.002	0.032	0.194	0.002	0.032
	50	0.201	0.000	0.032	0.201	0.000	0.032	0.201	0.000	0.032	0.200	0.002	0.032
rv = 5, cor = .5	25	49.738	5.538	0.075	49.730	5.531	0.075	49.722	5.523	0.075	49.722	5.523	0.075
	50	49.740	5.328	0.073	49.729	5.323	0.073	49.755	5.334	0.073	49.733	5.447	0.074
	25	0.234	0.006	0.031	0.219	0.004	0.031	0.197	0.001	0.032	0.197	0.001	0.032
	50	0.200	0.000	0.032	0.200	0.000	0.032	0.200	0.000	0.032	0.200	0.001	0.032

Data-generating values were: $\phi ^2 = 50$ and $\phi ^2 = 0.2$.

$^\mathrm{a}$se = $\frac{\sqrt{\frac{1}{S-1}\sum _s(\hat{\phi }^2_s - \phi ^2)^2}}{\sqrt{S}}$, where *S* is the total number of simulation runs.

**Table 3:** Overview of results at the end of the data stream: mean absolute error (MAE), root-mean-squared error (RMSE), and the empirical 95% confidence interval.

Condition	SEMA				SU			
	*MAE*	RMSE	$CI_{low}$	$CI_{up}$	*MAE*	RMSE	$CI_{low}$	$CI_{up}$
$$\beta $$								
rv = 1	0.375	0.476	99.057	100.931	0.366	0.464	99.067	100.931
	0.008	0.010	0.080	0.120	0.008	0.010	0.080	0.120
rv = 5, cor = 0	0.393	0.492	99.041	101.016	0.380	0.475	99.075	100.954
	0.014	0.018	0.064	0.136	0.014	0.018	0.064	0.136
rv = 5, cor = .15	0.377	0.472	99.073	100.883	0.362	0.455	99.105	100.856
	0.014	0.018	0.067	0.135	0.014	0.018	0.067	0.135
rv = 5, cor = .5	0.345	0.432	99.183	100.858	0.325	0.409	99.223	100.815
	0.015	0.018	0.062	0.134	0.014	0.018	0.063	0.133
$$\phi $$								
rv = 1	1.802	2.286	45.293	54.279	1.803	2.287	45.278	54.270
rv = 5, cor = 0	1.800	2.254	45.745	54.065	1.803	2.257	45.722	54.048
	0.011	0.014	0.173	0.227	0.011	0.014	0.173	0.227
rv = 5, cor = .15	1.768	2.168	45.660	53.883	1.771	2.171	45.635	53.849
	0.012	0.015	0.173	0.229	0.012	0.015	0.174	0.229
rv = 5, cor = .5	1.851	2.322	45.406	54.469	1.852	2.322	45.413	54.451
	0.011	0.014	0.174	0.229	0.011	0.014	0.174	0.229
$$\sigma ^2$$								
rv = 1	0.025	0.031	4.941	5.064	0.025	0.031	4.941	5.064
rv = 5, cor = 0	0.027	0.034	4.934	5.064	0.027	0.034	4.934	5.065
rv = 5, cor = .15	0.027	0.034	4.936	5.070	0.027	0.034	4.935	5.070
rv = 5, cor = .5	0.027	0.034	4.931	5.066	0.027	0.034	4.931	5.066

Condition	EM				SWEM			
	*MAE*	RMSE	$CI_{low}$	$CI_{up}$	*MAE*	RMSE	$CI_{low}$	$CI_{up}$
$$\beta $$								
rv = 1	0.350	0.445	99.124	100.880	0.347	0.443	99.109	100.877
	0.008	0.010	0.080	0.120	0.020	0.025	0.052	0.146
rv = 5, cor = 0	0.355	0.447	99.160	100.908	0.357	0.449	99.158	100.928
	0.014	0.018	0.065	0.136	0.026	0.033	0.036	0.162
rv = 5, cor = .15	0.341	0.431	99.133	100.852	0.344	0.431	99.139	100.838
	0.014	0.018	0.067	0.136	0.025	0.032	0.042	0.162
rv = 5, cor = .5	0.298	0.375	99.24	100.744	0.308	0.386	99.238	100.725
	0.014	0.018	0.063	0.134	0.025	0.031	0.035	0.161
$$\phi $$								
rv = 1	1.804	2.287	45.276	54.253	1.822	2.302	45.267	54.377
rv = 5, cor = 0	1.807	2.261	45.675	54.024	1.830	2.302	45.480	54.163
	0.011	0.014	0.173	0.227	0.032	0.040	0.129	0.280
rv = 5, cor = .15	1.774	2.174	45.615	53.819	1.790	2.200	45.479	53.724
	0.012	0.015	0.174	0.228	0.031	0.039	0.134	0.284
rv = 5, cor = .5	1.853	2.321	45.466	54.471	1.874	2.348	45.305	54.451
	0.011	0.014	0.174	0.229	0.026	0.033	0.142	0.270
$$\sigma ^2$$								
rv = 1	0.025	0.031	4.941	5.064	0.060	0.075	4.849	5.134
rv = 5, cor = 0	0.027	0.034	4.934	5.065	0.076	0.095	4.820	5.185
rv = 5, cor = .15	0.027	0.034	4.935	5.070	0.076	0.096	4.826	5.203
rv = 5, cor = .5	0.027	0.034	4.931	5.065	0.073	0.091	4.818	5.183

Data-generating values: $\beta = 100$ and .1; $\phi ^2 = 50$ and .2; $\sigma ^2 = 5.0$.

In Figs. 2 and 3, the four panels present the different conditions. In each panel, error bars depict the 95% empirical confidence interval. The first cluster of bars belongs to $n=25{,}000$ and the second cluster to the end of the data stream. Except for SWEM, the lengths of these bars are highly comparable for both the average residual variance (Fig. 2), as the moving average, absolute prediction error (Fig. 3). The moving average absolute prediction error was computed as follows: the window consists of 1000 data points and moves with 100 data points at the time.

The result obtained in this simulation study, combined with the simulation studies presented in supplementary material, clearly demonstrates the competitive performance of SEMA compared to EM. These studies show that the obtained estimates are similar and have similar variance. However, it has to be noted that—as expected based on our theoretical analysis of the computation complexity—the difference in computation time between SEMA and EM is large. Focusing just on condition A, we find that on average the simulation runs took 147.6 s per run for SEMA (including the training set of 2000 data points), while they took 1255.8 s for traditional EM. This is true despite the fact that SEMA provides updated estimates for *each individual data point during the data stream*, while our implementation of EM only updates its estimates once every 1000 data points.

## SEMA in Action: Predicting Weight Fluctuations

In this section, the SEMA algorithm is applied to an actual data stream originating from an experiment done by Kooreman and Scherpenzeel (2014). Using this application, we illustrate the practical issues that occur when analyzing data streams: we need to choose appropriate starting values, decide on an update regime of full SEMA updates, and deal with possible changes in the data-generating process that occur over time. In particular, the latter issue is instructive in this study; about 300 participants were added to the study after approximately two years of running the study. The application also highlights how the specification of random effects that depend on the time in the stream itself (e.g., days of the week, months, etc.) needs to be considered critically as, for the models to converge, we need observations at each possible level.

The study by Kooreman and Scherpenzeel (2014) concerned the fluctuations in individuals’ weight—over repeated measurements—in a longitudinal study using respondents from the Longitudinal Internet Studies for Social Sciences (LISS) panel. Among the respondents of the LISS panel, about 1000 *smart scales* were handed out. These smart weighting scales were equipped with an Internet connection. Respondents were instructed to use the scale barefoot, such that it could measure, among other variables, weight, percentage of muscle tissue, and percentage of fat tissue. The smart scale sent the data to a central server, where the data were combined with respondents’ survey data. The smart scales were handed out in the beginning of 2011, and the data collection continued until February 2014. While the data set contains the data from roughly 3 years, the authors used the data of 2011 only. We, however, analyze the full available data stream.

**Table 4:** Average mean absolute error (MAE) and average root-mean-squared error (RMSE) of the 1000 simulation runs.

Condition	Error	SEMA			SU			EM			SWEM		
		Mean	$CI_{low}$	$CI_{up}$	Mean	$CI_{low}$	$CI_{up}$	Mean	$CI_{low}$	$CI_{up}$	Mean	$CI_{low}$	$CI_{up}$
rv = 1	MAE	1.860	1.847	1.872	1.860	1.847	1.872	1.870	1.857	1.883	1.919	1.906	1.933
	RMSE	2.349	2.333	2.365	2.349	2.333	2.365	2.372	2.352	2.392	2.432	2.411	2.453
rv = 5, cor = 0	MAE	2.058	2.043	2.072	2.057	2.042	2.072	2.075	2.060	2.090	2.273	2.254	2.292
	RMSE	2.631	2.610	2.651	2.630	2.610	2.650	2.665	2.642	2.689	2.912	2.883	2.940
rv = 5, cor = .15	MAE	2.055	2.041	2.069	2.054	2.040	2.068	2.072	2.057	2.086	2.267	2.250	2.285
	RMSE	2.628	2.608	2.649	2.627	2.607	2.647	2.662	2.638	2.684	2.906	2.881	2.931
rv = 5, cor = .5	MAE	2.031	2.017	2.045	2.030	2.016	2.045	2.046	2.032	2.061	2.216	2.199	2.234
	RMSE	2.594	2.574	2.615	2.593	2.573	2.613	2.627	2.603	2.652	2.839	2.813	2.866

Average MAE = $\frac{1}{1000}(\frac{\sum _{i=1}^{n}|\hat{y}_i - y_i |}{n})$, $n = 48000$: the length of the data stream, without the training set.

Average RMSE = $$\frac{1}{1000}(\sqrt{\frac{\sum _{i=1}^{n} (\hat{y}_i - y_i)^2 }{n}})$$.

Since the data include time stamps, we were able to replay the data stream from 2011 till February 2014. Thus, in this evaluation of SEMA, the data of Kooreman and Scherpenzeel ($n = 78{,}021$, $J = 883$) were combined with the data of the remaining years. The first experimental factor of interest was the (instructed) frequency of the scale usage: every day, every week, or not specified. The second factor was the feedback respondents received: their weight and the norm what they should weigh, their weight and their goal weight, or only their weight. Both experimental factors were crossed, resulting in nine conditions of interest. Before running SEMA on the data stream we removed a number outliers (0.1% of the data), for which weight fluctuated with more than 5 kg within a day for a single respondent. The remaining data set consisted of $n = 288,521$ observations from a total of $J = 1269$ respondents. In Table 5, we present an overview of the model fitted to the data stream by indicating the variables included as fixed or random, as well as the number of levels (or categories) of each of the variables.

**Table 5:** Fitted model to the smart-scale data stream.

Variables	Fixed	Random	Number of categories	Reference
Intercept	$\checkmark$	$\checkmark$		
Day of the week	$\checkmark$	$\checkmark$	7	Friday
Gender	$\checkmark$		2	Male
Year of birth	$\checkmark$		–	1970 (centered)
Length	$\checkmark$		–	174cm (centered)
Feedback	$\checkmark$		3	Only weight
Frequency	$\checkmark$		3	Not specified
Time of measurement	$\checkmark$		4	Morning

The dependent variable is *weight*.

**Fig. 4:**
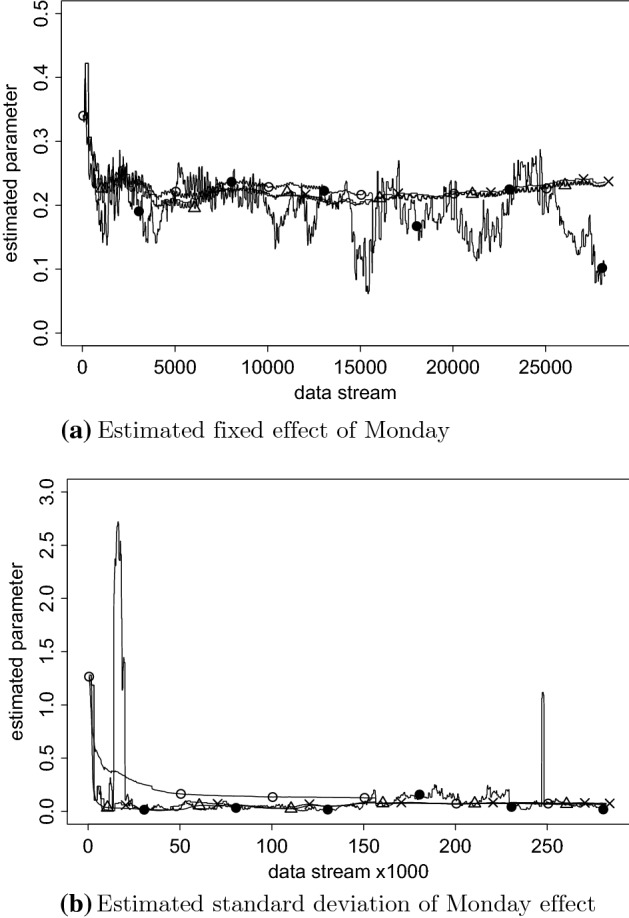
The estimated Monday effect and its standard deviation. The ‘$\times$’ is EM, triangle is SEMA Update, open circle is SEMA, and closed circle is Sliding Window EM, the most right ‘$\times$’ is EM using all data and 2000 iterations.

**Fig. 5:**
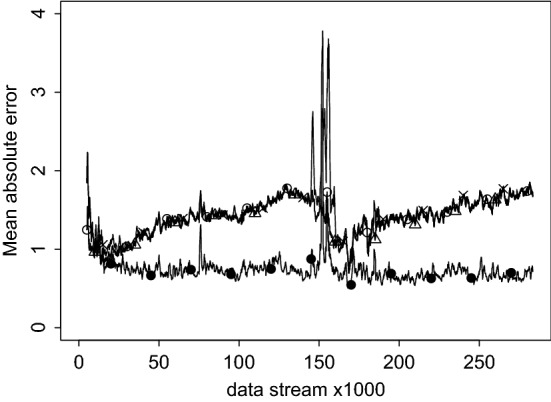
Mean absolute error (MAE), a moving average of 1000 data points, shifting with 500 data points at a time.

Since the authors of the original paper focused on the “effect of Monday,” which implies that on average individuals were 0.2 kg heavier on Mondays than on Fridays, we similarly focus on the estimation of this “Monday” effect. In this application, we used the same methods as presented in the simulation study. To ensure that we have good starting values, we used the first 2 months of data ($n = 6894$, $J = 472$) as a training set.^3^ Another practical decision is when to update the offline EM algorithm. For this study, we chose to rerun the EM algorithm every Sunday night, using a maximum of 1000 iterations. The sliding window implementation EM used a window of 12,000 data points, which is approximately equal to 2 months of data. SWEM and SEMA Update performed an additional update every night, where SWEM was allowed a maximum of 100 iterations and SU was allowed 2 EM cycles, since the model is rather large and the data rather noisy. In January 2013, new participants were added to the study, a large new group of about 300 new participants. To deal with this sudden—but known—change in the data-generating process, we retrain the model using all data observed so far including the newly recruited participants (which is at $n=163{,}000$). In order to do so, the EM algorithm was run until convergence with a maximum of 2000 iterations and after which the parameter estimates of EM algorithm were used as input for the other methods.

In Fig. 4, the estimates of the fixed effect of Monday are compared to Friday (a), the standard deviation of the effect of Monday (b). In Fig. 5, the moving average absolute prediction error is illustrated. Please find all remaining parameter estimates again in supplementary material online. The open circles indicate the SEMA algorithm, the triangles SEMA update, the ‘$\times$’ is EM and the black solid circle is Sliding Window EM. While all methods seem to illustrate a rather similar fluctuation of the Monday effect over time, the sliding window EM (SWEM) implementation fluctuates more than the other methods since it only uses about 2 months of the most recent data points. Finally, in Fig. 5, the moving average absolute prediction error of all four fitting procedures is presented. The window consists of 1000 data points, and the window shifts 100 points at a time. The high outlier from both the EM and SWEM is due to the fact that in that point in the data stream new participants were included in the stream. SWEM somewhat outperforms the other methods, most likely because in fact the data-generating mechanism changes over time, a change the other methods are insensitive to.

To conclude, based on our results, there seems to be some evidence in favor of a “Monday effect.” However, this result should be interpreted with care for several reasons. First, while three out of the four estimation methods replicate the findings reported on by Kooreman and Scherpenzeel (2014), SWEM shows a sharp decrease in effect size toward the end of the stream. Hence, the estimated effect seems variable over time. Second, it has to be noted that the estimated variance of the Monday effect is very large compared to its average effect. This implies that while there is some evidence in favor of a “Monday effect” on average, the variance of the effect between participants is large, and thus, the average effect is a poor description of the underlying true mechanism. Hence, we would conclude that while an average effect of Monday exists in the data analyzed by Kooreman and Scherpenzeel (2014), the effect seems unstable and very variable between participants. Note that the differences between the EM, SEMA, and SU estimates are negligible, and SEMA thus seems well suited to analyze the current data stream.

## Discussion

In this paper, we developed an extension of the Streaming Expectation Maximization Approximation (SEMA) algorithm of which a rudimentary version was supplied in Ippel, Kaptein, and Vermunt (2016a). In its original conception, SEMA was able to estimate simple multilevel models that contained only level-2 fixed effects and a single random intercept. The extension we discuss in the paper enables researchers to fit much more flexible multilevel models that include fixed effects at level-1 (e.g., repeated measurements), level-2 (e.g., individual characteristics), and multiple random intercepts and random slopes. This extension is not trivial: compared to the initial specification by Ippel, Kaptein, and Vermunt (2016a), the E-step of SEMA algorithm has been totally revised to deal with the covariances resulting from the larger number random effects. This change directly influences the specification of the CDSS and their update rules. In this paper, we have shown that—due to its online estimation method—SEMA is computationally more efficient than traditional fitting procedures. We have demonstrated in two extensive simulations and one application that this computational efficiency comes at very modest costs: the estimates resulting from SEMA are very close to the current state of the art.

Commonly used methods to fit multilevel models (e.g., EM algorithm or Newton–Raphson) repeatedly pass over the data set to estimate the model parameters. When new data enter, these procedures are repeated to update the model parameters including the new data. Especially when the number of random effects is large, many passes over the data are required to obtain stable estimates of the model parameters. In such cases, these traditional fitting procedures quickly become infeasible for large data sets or continuous data streams. SEMA, on the other hand, only uses each data point only once, after which it can be discarded. SEMA thus estimates the model parameters in a computationally less complex manner than the common procedures since it does not have to revisit the same data repeatedly. Therefore, SEMA can be used to analyze data streams while accounting for the nested structure that is often observed in data streams. SEMA also effectively deals with the problems of storing extremely large data sets: the information from each individual data point is aggregated to the level of individuals and hence more easily stored. Our algorithm enables researchers to use multilevel models for prediction purposes in real time. In a simulation study, we showed that even when the number of observations per individual is small and the number of parameters is large, parameter estimates were estimated accurately. Furthermore, we showed that the predictive performance of SEMA was competitive to traditional fitting procedures.

Alongside the development of SEMA, many related methods are currently being developed to analyze data streams. For instance, variational inference, expectation propagation, and sequential MCMC (sMCMC) sampling are actively explored (Bayesian) methods to deal with large data sets. Variational methods speed up posterior computations by replacing the (global) posterior, which often has an unknown distributional form, by a distribution with a known distributional form (Broderick, Boyd, Wibisono, Wilson, and Jordan, 2013; Kabisa (Tchumtchoua), Dunson, and Morris, 2016). (Stochastic) Expectation propagation similarly approximates the posterior; however, it does so locally (Li, Hernández-Lobato, and Turner, 2015). SMCMC provides an appealing extension to MCMC methods because the generated MCMC draws are updated as opposed to sampled anew when additional data enter (Yang and Dunson, 2013). The SEMA approach presented in this paper, which involves updating the likelihood during a data stream, could prove relevant to these fields of research by providing a computationally attractive method of updating the likelihood.

While the current extension of SEMA algorithm allows for fitting multilevel models with fixed and random effects in data streams, extensions are possible and need further development. First, the SEMA algorithm builds on the EM algorithm to fit a *linear* multilevel model. However, the EM algorithm is also used to fit nonlinear models whose likelihood is a member of the exponential family. Using the strong link with the EM algorithm, SEMA can potentially also be used to fit a range of alternative models which deal with multilevel data. Examples include the negative binomial which is a combination of the beta distribution with a Poisson distribution, or beta binomial function which is a combination of, respectively, a beta and a binomial distribution. These extensions are yet to be developed.

Second, SEMA, and its current [R] implementation, could be extended further by implementing efficient parallelization. For truly massive data sets, in which the number of participants *J* is extremely large, one might encounter a situation in which the storage—and subsequent update—of all $\theta _j$’s on a single machine is infeasible. In these cases, we can store subsets of the $\theta _j$’s on different machines—each of which can efficiently be retrieved using hashing. Next, we can use the current $\theta$, and the respective $\theta _j$ to compute an update of $\theta _j$; as $\theta$ will change slowly in a massive data stream, we can choose to batch update $\theta$ occasionally, while we update the respective $\theta _j$’s each in parallel on different machines as the data points arrive.

Third, in Kooreman and Scherpenzeel (2014)—our empirical example—the authors actually used a multilevel model with more fixed effects than the model we used in this paper. The original model also contained fixed effects for the calendar months. Fitting this model requires observations in (almost) each month, such that the ${\varvec{X}}'{\varvec{X}}$ matrix becomes invertible (i.e., at least semi-positive definite). Consequently, using SEMA as it is formulated in this paper, a model including the effects of months cannot be fitted to the data *before* the data stream has run for almost a year. Further research should focus on extending the model *during* the data stream, such that these effects can be included dynamically once enough data have been collected.

### Fluctuations Over Time

In addition to modeling the repeated measurements of the same individuals using a linear multilevel model, there is a broad range of more complex models that could potentially deal with dependencies between observations. Cappé (2011), for instance, studied an online EM algorithm to fit hidden Markov Models. In such a model, the influence of the previous observation is included in the current estimation model. Also, when observations are equally spaced, models such as state-space models (Arulampalam, Maskell, Gordon, and Clapp, 2002) or autoregressive models [e.g., AR(1)] can be used as well to model fluctuations over time. Extending SEMA to cover these cases provides a promising direction for future work.

Furthermore, the current version of SEMA assumes that the true data-generating process is stationary and that, over the course of the data stream, we converge to the “correct” parameter estimates. However, when monitoring individuals over time, it is likely that the data-generating process itself changes over time, also known as *concept drift* (Widmer and Kubat, 1996). Sliding window approaches, in which only the most recent data points are included in the analysis, are often used in such cases: we examined SWEM as an example. In this case, the chosen window size is inherently somewhat arbitrary, and appropriate window sizes will depend on the problem at hand. In general, a larger window stabilizes the estimates with the risk of being less sensitive to concept drift, while a smaller window allows for the quick detection of concept drift with the risk of obtaining extremely high variance estimates.

Note that when using a sliding window approach one still reestimates the model parameters each time the window slides, albeit using only the data within the window. SEMA provides an alternative: a fixed learn rate could be used to limit the influence of the older data when dealing with data streams. In Eq. 1, it is easily seen that the “learn rate” for computing an online sample mean is $\frac{1}{n}$. Thus, as the stream becomes longer (and *n* grows larger) the learn rate decreases and the computed mean stabilizes. If, instead, we would alter the update rule of $\bar{x}$ to read $\bar{x} \leftarrow \bar{x} + \frac{x_t-{\bar{x}}}{{\texttt {min}}(n, \alpha )}$ for some fixed value of $\alpha$ of say 10,000, we effectively create a smooth moving window in the sense that older data points are *smoothly* discarded—though without revisiting older data points. This can, with some effort, similarly be implemented in SEMA. For instance, for the estimation of the fixed effects the influence of the existing $(\mathbf {X}'\mathbf {X})^{-1}$ could be decreased such that the new data points get more weight. Introducing such a ‘smooth sliding window’, where previous data gradually receive less weight, provides a way of dealing with changing (true) parameter values.

### Missing Data

In a data stream, in addition to not observing all *p* covariates for each data point, often not all covariates enter at the same time. Some information might be missing or might be observed later, e.g., learning the gender of a respondent after already receiving a number of data points. Missingness is a research area on its own (Donders, van der Heijden, Stijnen, and Moons, 2006; van der Palm, van der Ark, and Vermunt, 2016), but the types of missingness generated in data streams raise new research questions. For example, related to the issue of item nonresponse, is the issue of unit nonresponse due to attrition. If a subgroup of respondents, e.g., the less affluent respondents, drop out of the study, the parameter estimates of the model could become biased. As SEMA only updates the CDSS contributions when an individual returns her contributions will become outdated if she does not return. While we do not explicitly study solutions to attrition in data streams, additional runs over the individuals (is implemented in “SEMA Update”) could be used to update all contributions to the CDSS. Alternatively, one could also choose to update the contributions of those who do not return within a given period of time (which is related to the partial EM algorithm, see, Neal and Hinton, 1998; Thiesson, Meek, and Heckerman, 2001). Note that both types of missingness, unit and item, are issues to be dealt with in future research on data streams.

### Closing Remarks

Continuous data collection is slowly becoming pervasive in the social sciences: popular data collection methods such as experience sampling and novel sensing technologies provide continuous data streams of human behavior. Often these data have a nested structure: observations are nested within individuals and the dependencies introduced by this nesting should be accounted for in the analysis. In this paper, we presented the SEMA algorithm, a computationally efficient algorithm to analyze data that contain a nested structure and arrive in a continuous fashion. Hence, multilevel models with numerous fixed and random effects can now be fit to continuous data streams (or extremely large static data sets), in a computationally efficient fashion.

## Electronic supplementary material

Below is the link to the electronic supplementary material.

Supplementary material 1 (pdf 3731 KB)

## Footnotes

1 See, e.g., Ippel, Kaptein, and Vermunt (2016b).

2 For more details and proof, see Raudenbush and Bryk (2002), Chapter 14.

3 Due to logistic reasons, not all smart scales were handed out at that moment.
