# Supplementary material for: Estimating Multilevel Models on Data Streams
Source: Psychometrika. 2019 Jan 22;84(1):41–64. doi: 10.1007/s11336-018-09656-z (PMC6373343; doi:10.1007/s11336-018-09656-z)
Supplement: Supplementary file 1 — Supplementary material 1 (pdf 3731 KB) [file 11336_2018_9656_MOESM1_ESM.pdf]

Supplementary Material of
Estimating Multilevel Models on Data Streams

December 6, 2018

1 Introduction

In this supplementary matrial additional, results of the first simulation study and the applications, and two additional simulation studies are presented. First are the results presented of the simulation study which is presented in the paper. We first show the estimated coefficients of the fixed effects, followed by the variances of the random effects and the correlations between these random effects. Then, Section 3 contains the remaining parameters of the application: fluctuations in weight by Koorenman and Scherpenzeel (2014). Here, we present the estimated coefficients of the fixed effects of this model (except Monday which is in the original paper), the same holds for the variance of the random effects. We also present the correlations between the random effects and, lastly, the residual variance. This document finishes with 2 smaller studies: 1) we show that even if starting values are less favourable SEMA preforms adequately, and 2) when there is a dependency between the value of the random effect $\mathbf{b}_j$ and the point of entering in the data stream, parameter estimates are only slightly affected.

2 Results simulation study 1

2.1 Fixed effects

Here, we present all the results of the simulation study, which due to the large number of results would make the paper too lengthy. The simulation study consists of 4 conditions, which differ as follows:

- Condition A: Random intercept (i.e., 1 random effect)
- Condition B: 5 Random Effects - no correlation between the random effects
- Condition C: 5 Random Effects - correlation between the random effects is equal to .15
- Condition D: 5 Random Effects - correlation between the random effects is equal to .5

All conditions of the simulation study contain 15 fixed effect coefficients: Intercept, 3 continuous level 2 variables, 1 dummy variable (like ‘gender’) and 1 categorical variable with 3 categories, i.e., 2 dummy variables, 5 continuous level 1 variables and 1 categorical variable with 4 categories, i.e., 3 dummy variables. The following 15 pages contain the estimated parameter estimates of all fixed effects. You find the data generating values in each of the figures captions. All figures are structured similarly: Condition A is top left, Condition B is top right, Condition C is bottom left, and Condition D is bottom right. All figures contain a black line which indicates the true value. Especially, for the estimates of the fixed effects, all four methods produced very similar results which makes it nearly impossible to identify the separate methods. To differentiate the four methods, each line contains symbols:

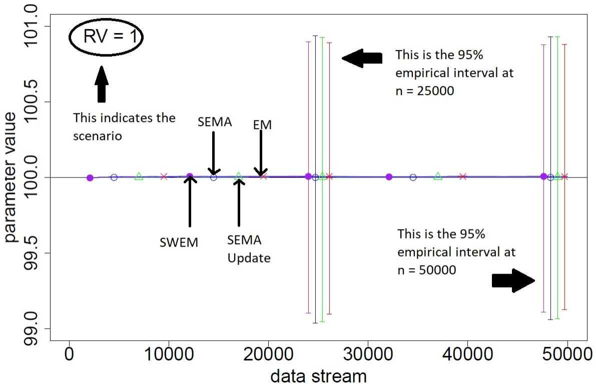

Figure (1) An example indicating how the figures are organized

- the blue line with open circle is SEMA,
- the green line with triangle is SEMA Update,
- the red line with '×' is EM,
- and the purple line with solid circle is Sliding Window EM.

Furthermore, each figure contains 2 sets of 4 bars. The first set is the 95% empirical interval of the simulation study at $n = 25,000$, the second set of bars indicate the 95% empirical interval at the end of the data streams. The color/symbol combinations identifying the different methods are also used for the bars, see also the example Figure 1. Note that from one page to the other, the Y-axes differ due to different data generating values and amount of true variance (with which the data were generated) or sampling variance.

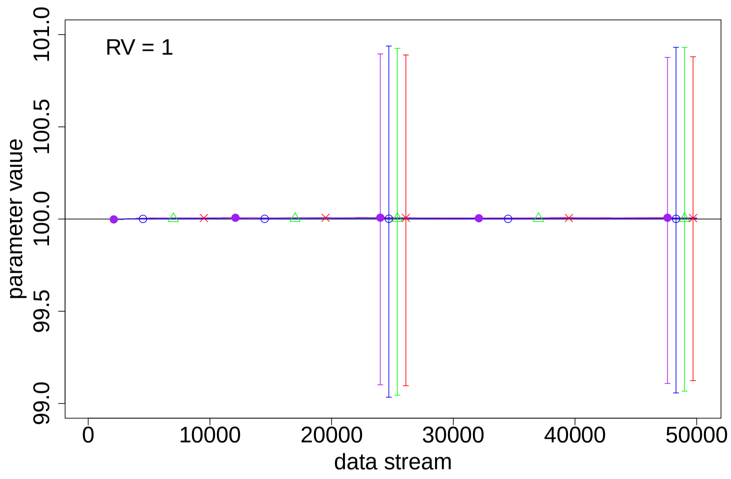

(a) Condition A

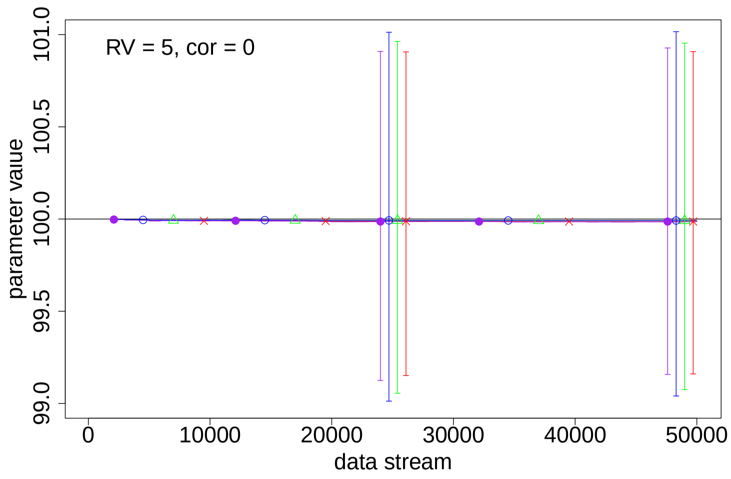

(b) Condition B

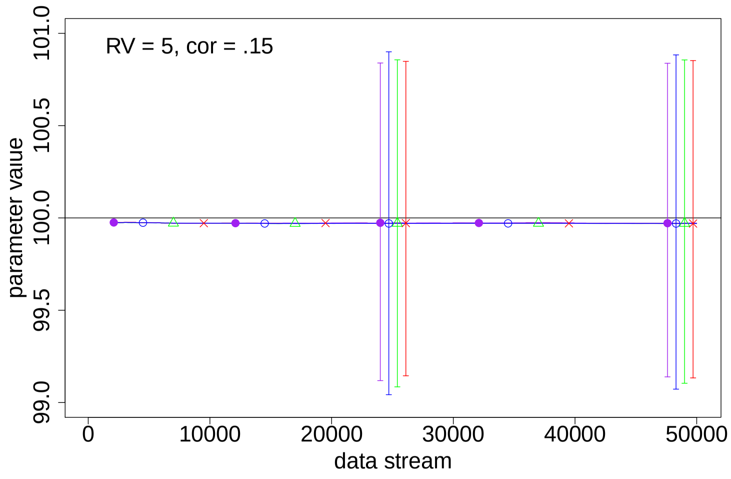

(c) Condition C

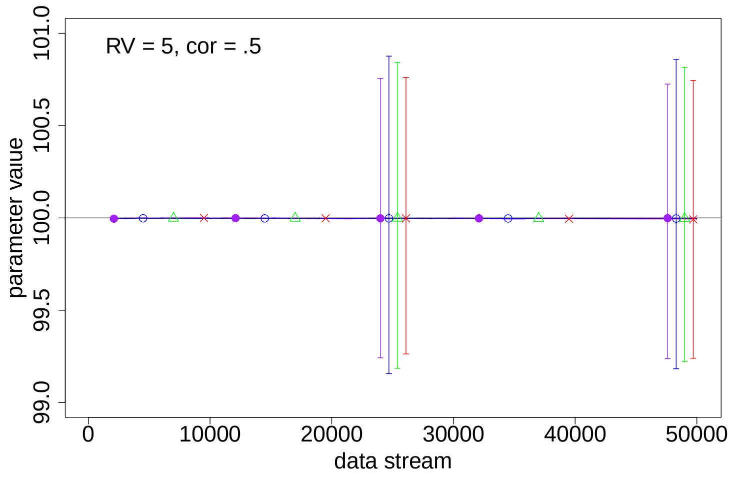

(d) Condition D

Figure (2) Estimated fixed coefficient of the intercept. The true value is 100, and the variance of the intercept is equal to 50. The error bars indicate the 95% empirical interval of the 1000 simulation runs. The blue line with open circle is SEMA; the green line with triangle is SEMA Update; the red line with '×' is EM; and the purple line with solid circle is Sliding Window EM.

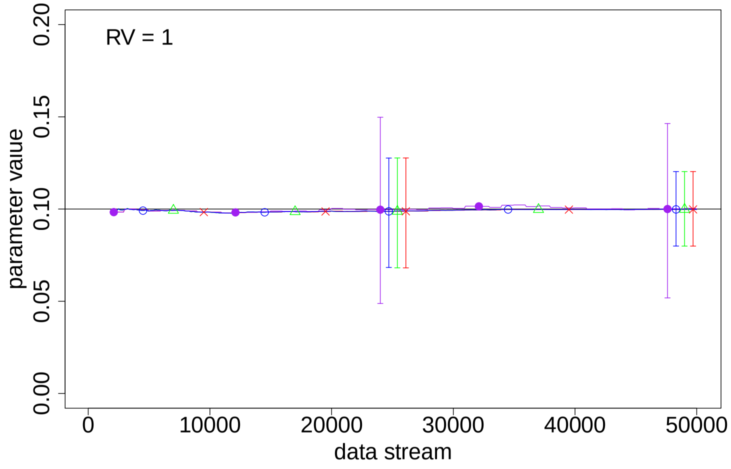

(a) Condition A

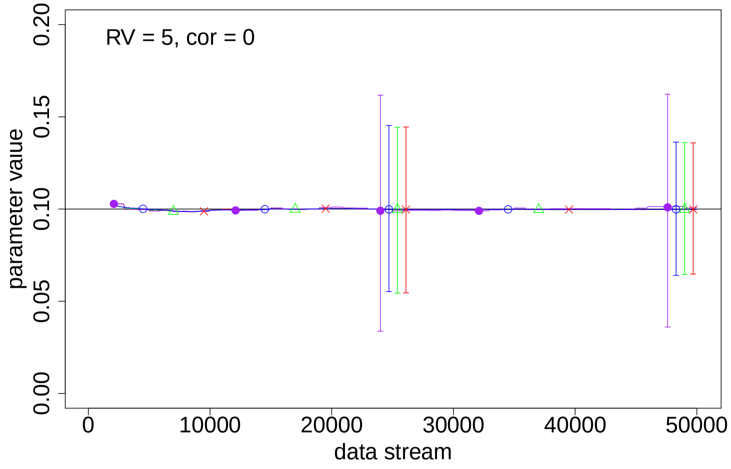

(b) Condition B

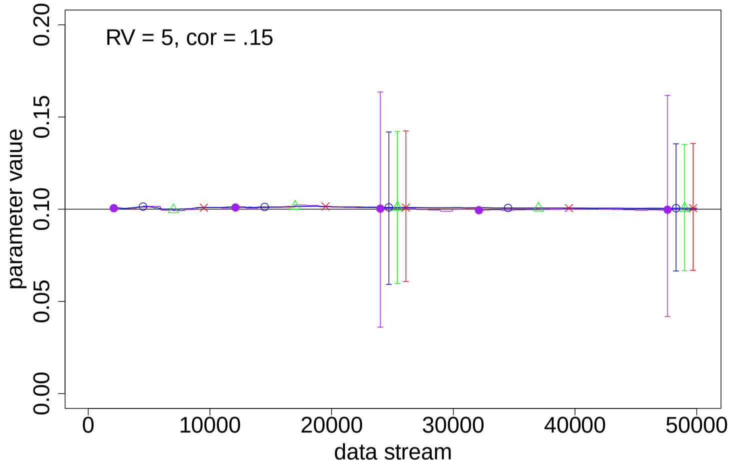

(c) Condition C

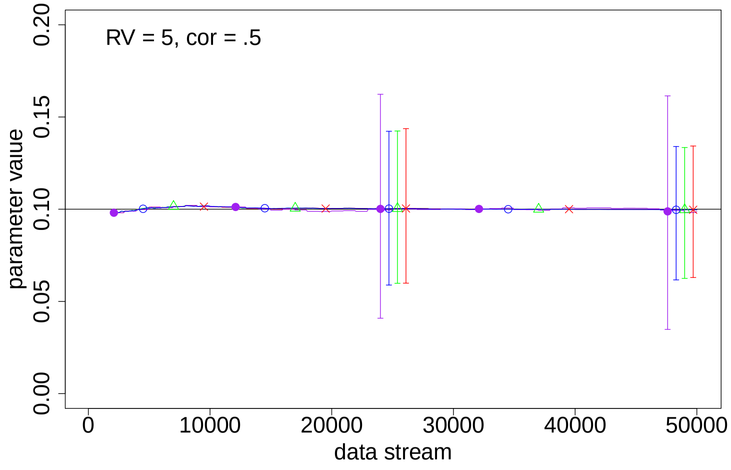

(d) Condition D

Figure (3) Estimated fixed coefficient of level 1 covariate. The true value is .1, and for Conditions B:D the variance of the intercept is equal to .2. The error bars indicate the 95% empirical interval of the 1000 simulation runs. The blue line with open circle is SEMA; the green line with triangle is SEMA Update; the red line with '×' is EM; and the purple line with solid circle is Sliding Window EM.

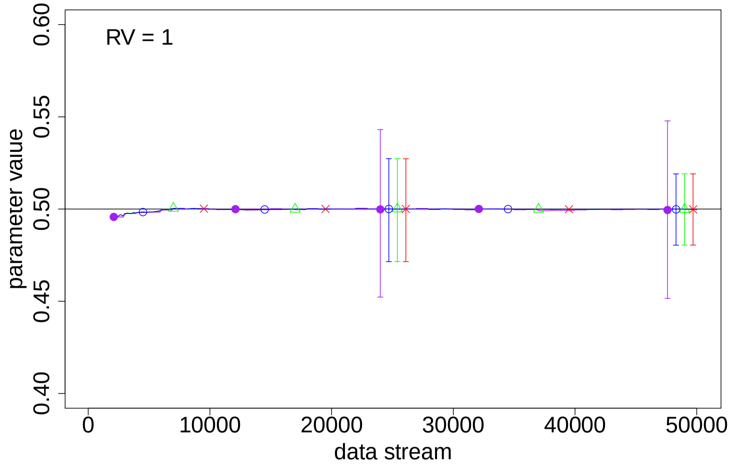

(a) Condition A

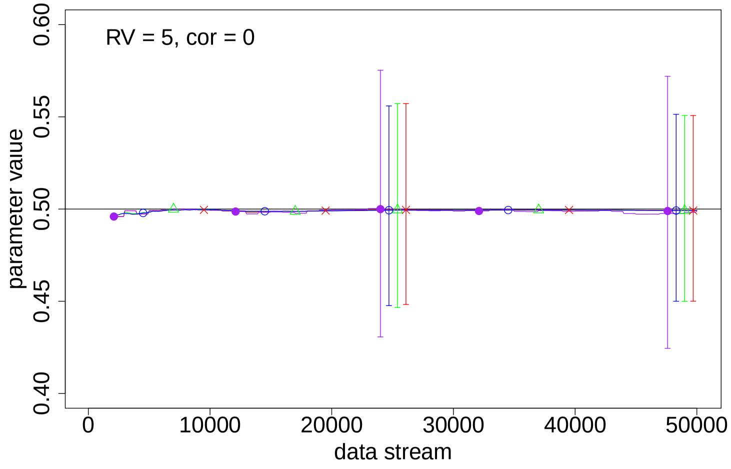

(b) Condition B

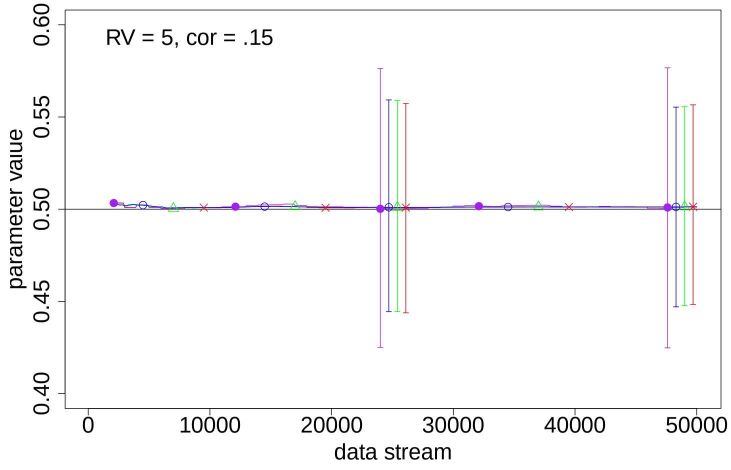

(c) Condition C

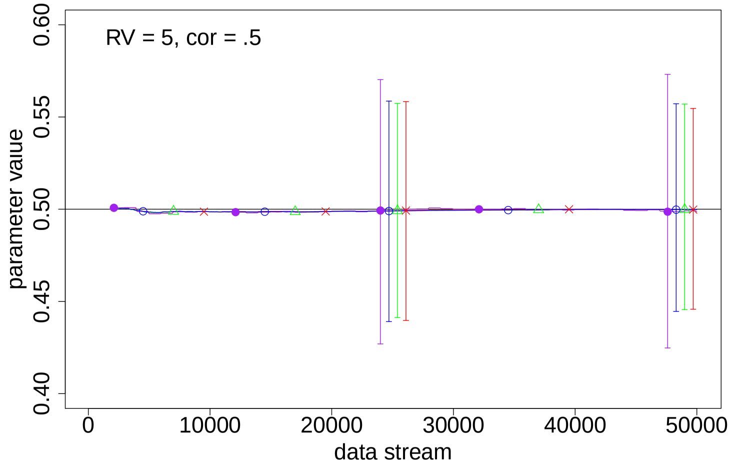

(d) Condition D

Figure (4) Estimated fixed coefficient of level 1 covariate. The true value is .5, and for Conditions B:D the variance of the intercept is equal to .6. The error bars indicate the 95% empirical interval of the 1000 simulation runs. The blue line with open circle is SEMA; the green line with triangle is SEMA Update; the red line with'×'is EM; and the purple line with solid circle is Sliding Window EM.

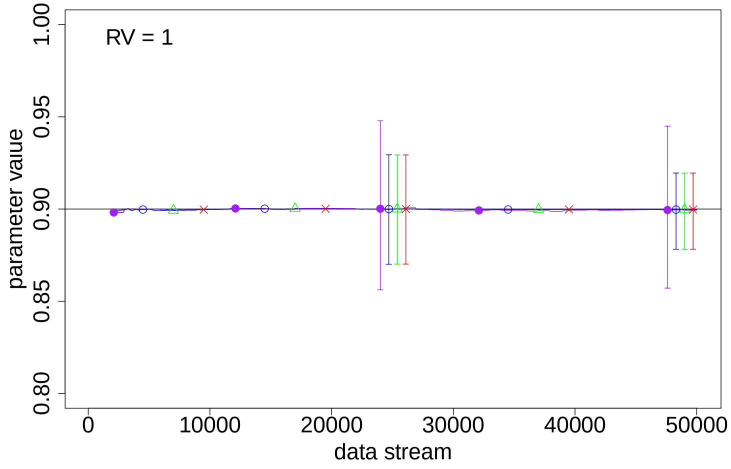

(a) Condition A

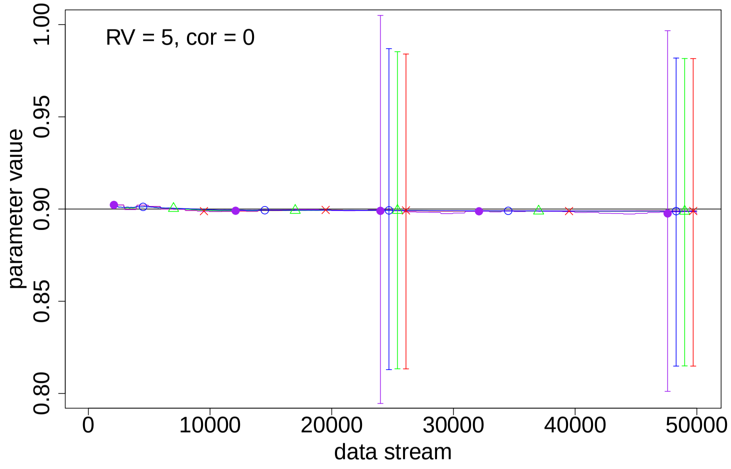

(b) Condition B

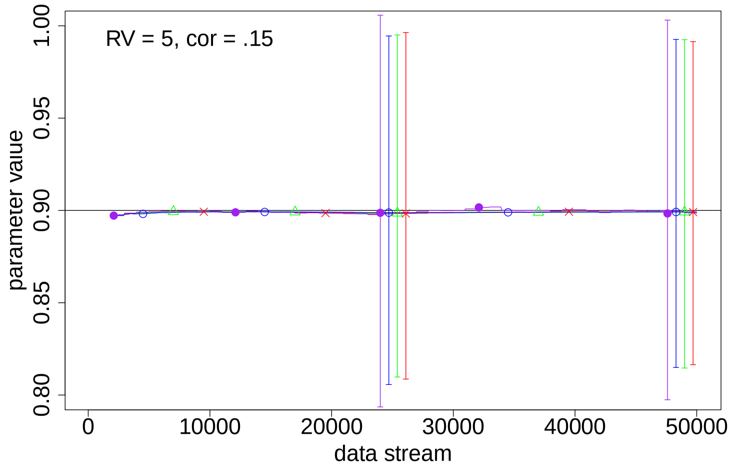

(c) Condition C

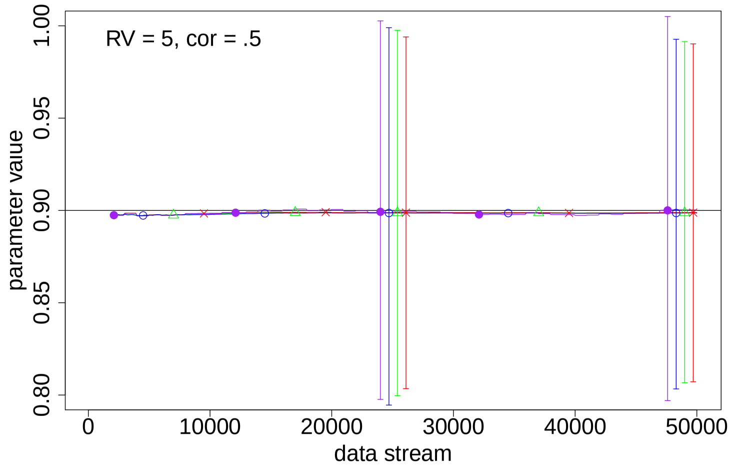

(d) Condition D

Figure (5) Estimated fixed coefficient of level 1 covariate. The true value is .9, and for Conditions B:D the variance of the intercept is equal to 1.8. The error bars indicate the 95% empirical interval of the 1000 simulation runs. The blue line with open circle is SEMA; the green line with triangle is SEMA Update; the red line with'×'is EM; and the purple line with solid circle is Sliding Window EM.

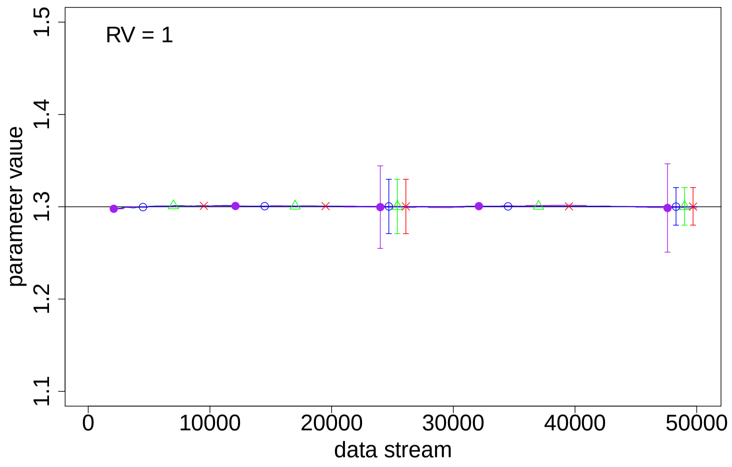

(a) Condition A

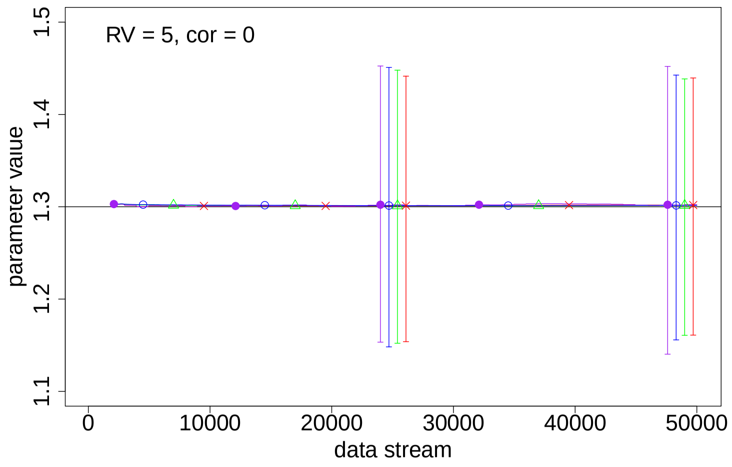

(b) Condition B

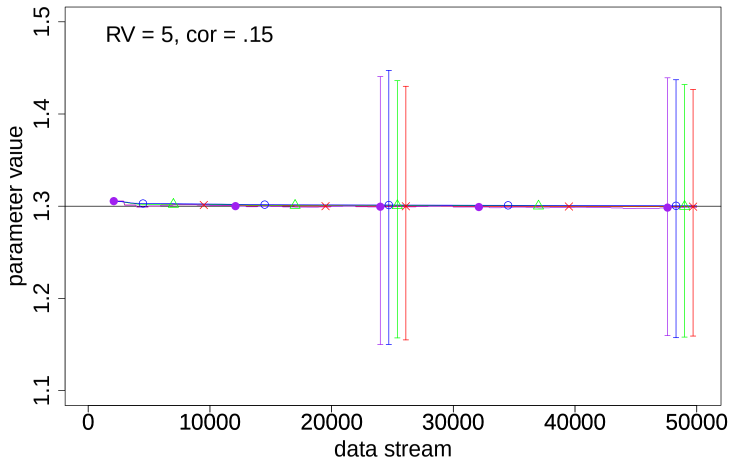

(c) Condition C

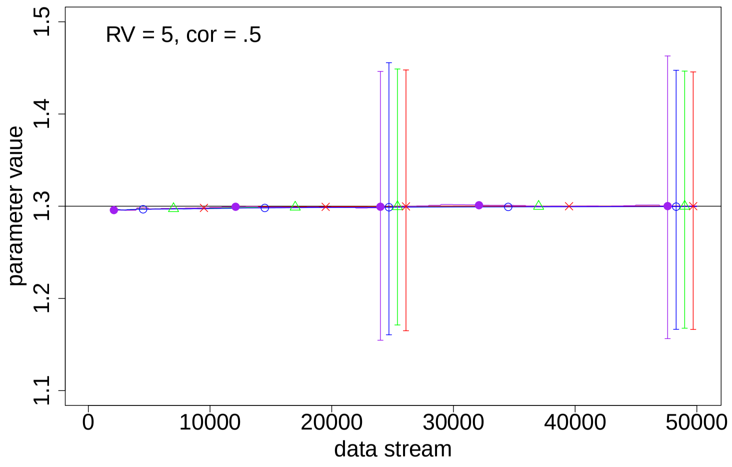

(d) Condition D

Figure (6) Estimated fixed coefficient of level 1 covariate. The true value is 1.3, and for Conditions B:D the variance of the intercept is equal to 5.0. The error bars indicate the 95% empirical interval of the 1000 simulation runs. The blue line with open circle is SEMA; the green line with triangle is SEMA Update; the red line with'×'is EM; and the purple line with solid circle is Sliding Window EM.

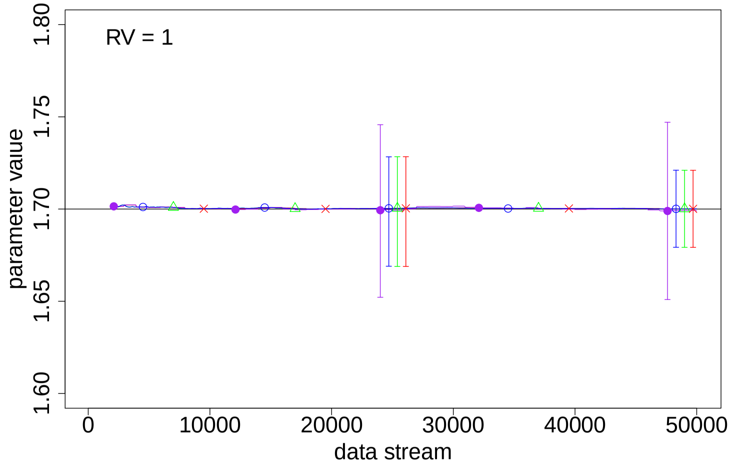

(a) Condition A

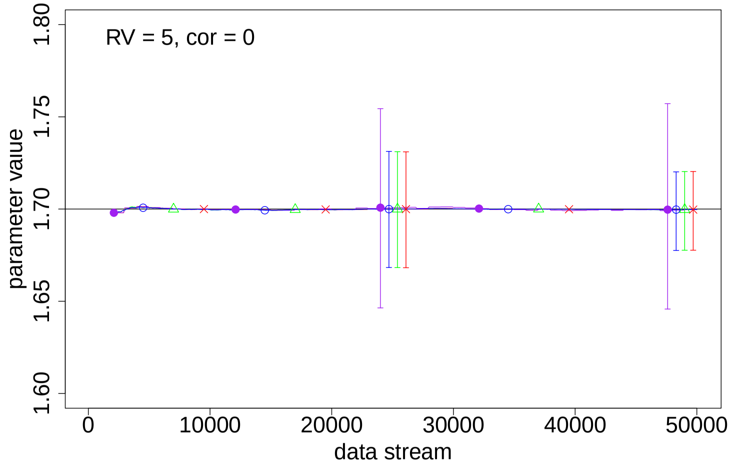

(b) Condition B

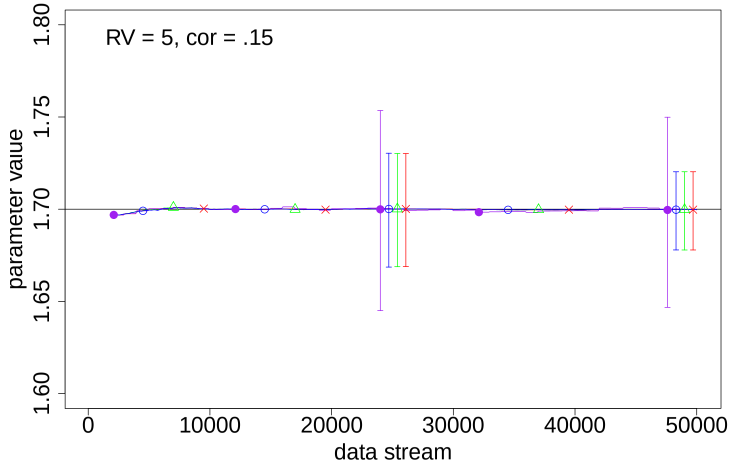

(c) Condition C

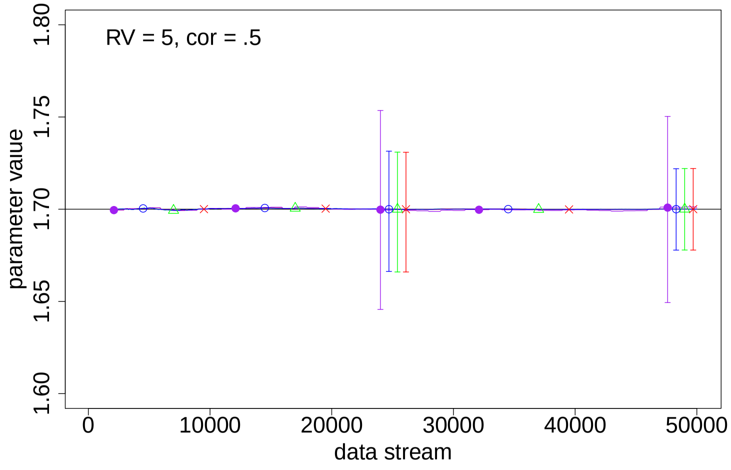

(d) Condition D

Figure (7) Estimated fixed coefficient of level 1 categorical variable, this is the first out of 3 dummy variables. The true value is 1.7. The error bars indicate the 95% empirical interval of the 1000 simulation runs. The blue line with open circle is SEMA; the green line with triangle is SEMA Update; the red line with‘×’is EM; and the purple line with solid circle is Sliding Window EM.

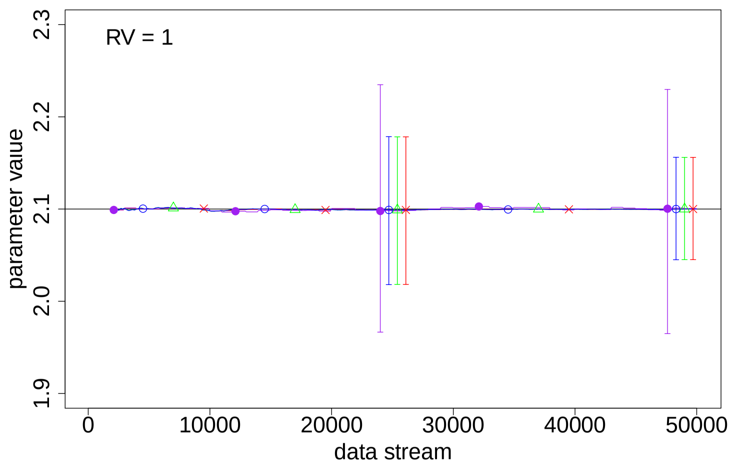

(a) Condition A

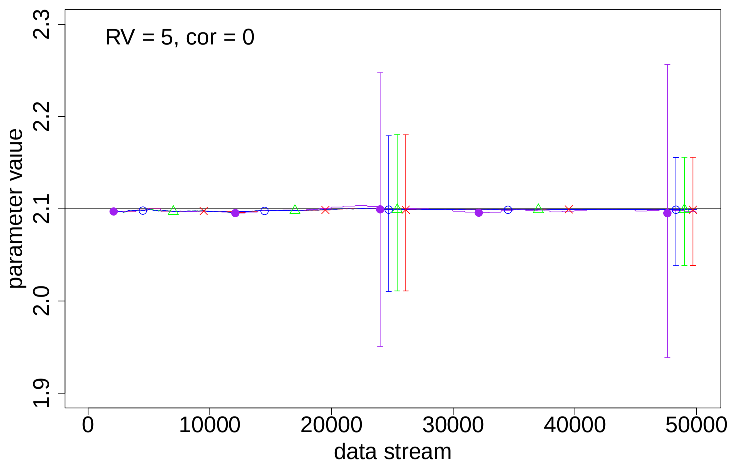

(b) Condition B

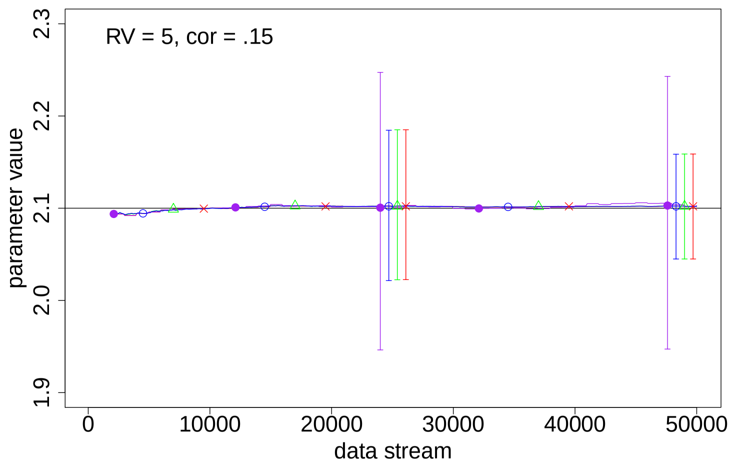

(c) Condition C

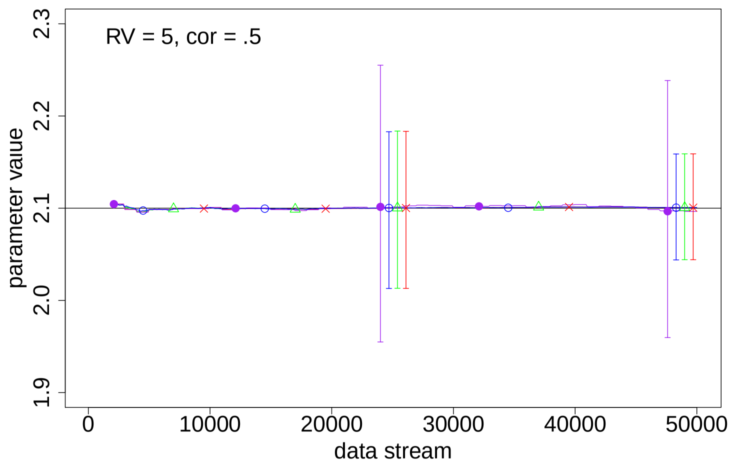

(d) Condition D

Figure (8) Estimated fixed coefficient of level 1 categorical variable, this is the second out of 3 dummy variables. The true value is 2.1. The error bars indicate the 95% empirical interval of the 1000 simulation runs. The blue line with open circle is SEMA; the green line with triangle is SEMA Update; the red line with'×'is EM; and the purple line with solid circle is Sliding Window EM.

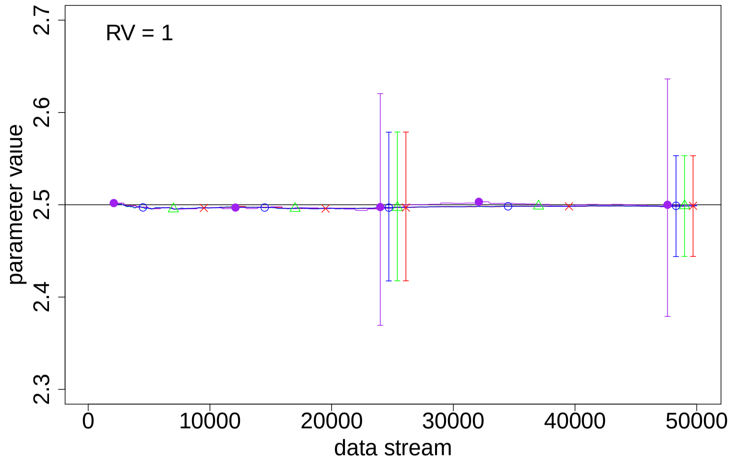

(a) Condition A

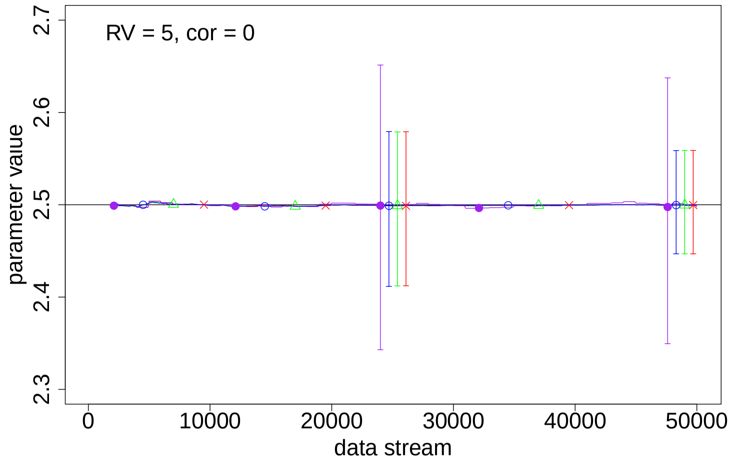

(b) Condition B

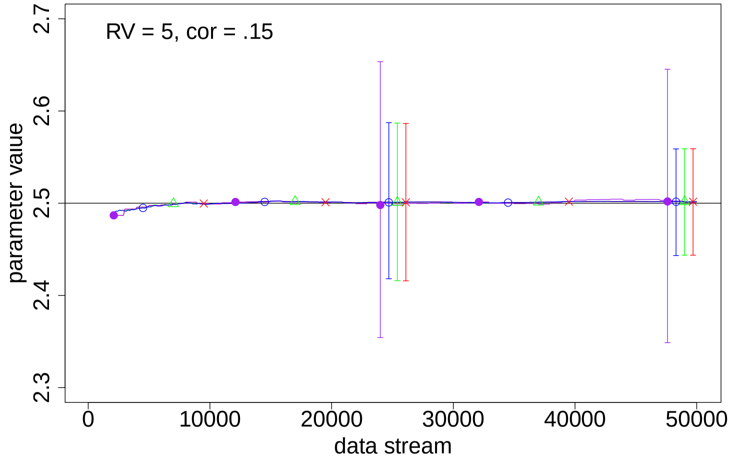

(c) Condition C

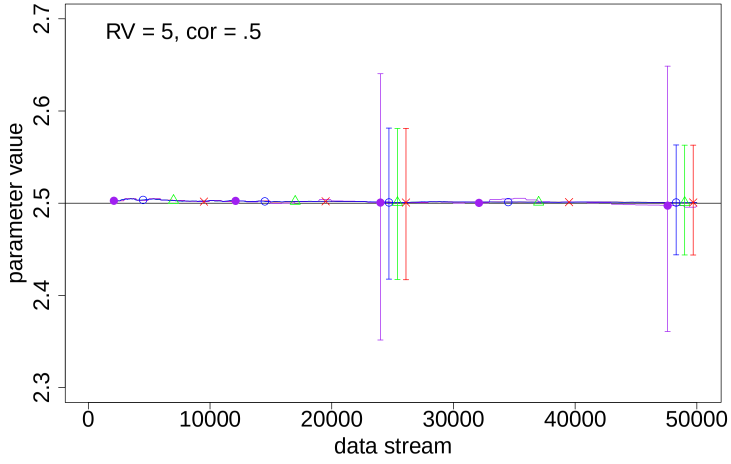

(d) Condition D

Figure (9) Estimated fixed coefficient of level 1 categorical variable, this is the last out of 3 dummy variables. The true value is 2.5. The error bars indicate the 95% empirical interval of the 1000 simulation runs. The blue line with open circle is SEMA; the green line with triangle is SEMA Update; the red line with'×'is EM; and the purple line with solid circle is Sliding Window EM.

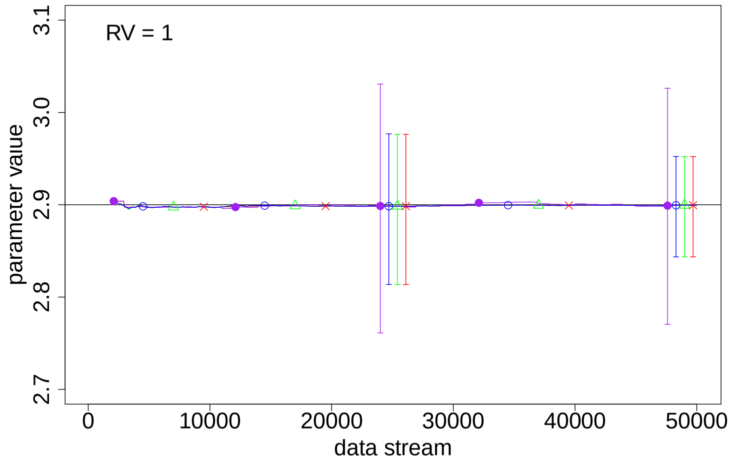

(a) Condition A

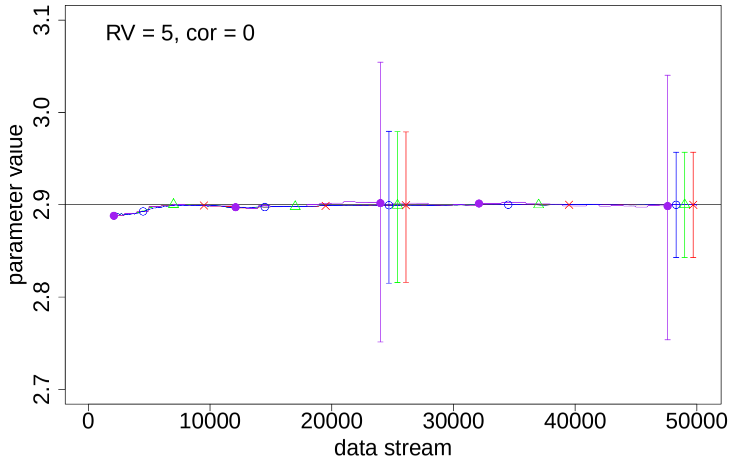

(b) Condition B

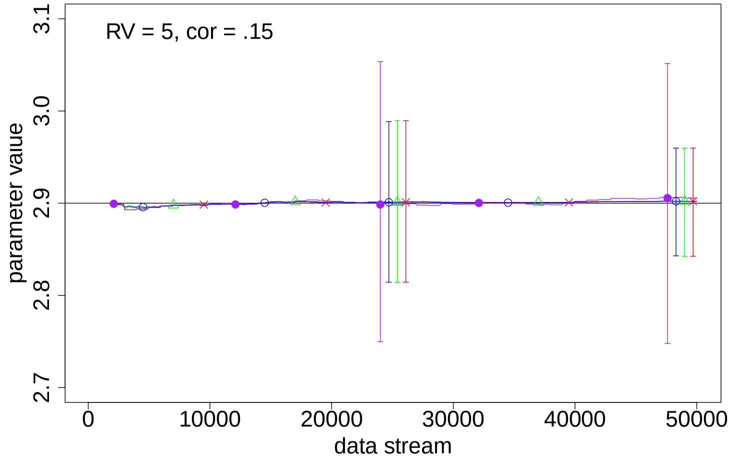

(c) Condition C

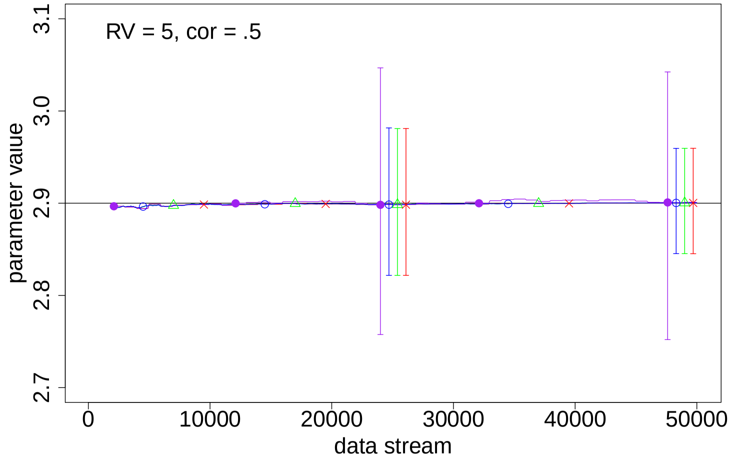

(d) Condition D

Figure (10) Estimated fixed coefficient of level 2 covariate. The true value is 2.9. The error bars indicate the 95% empirical interval of the 1000 simulation runs. The blue line with open circle is SEMA; the green line with triangle is SEMA Update; the red line with‘ × ’is EM; and the purple line with solid circle is Sliding Window EM.

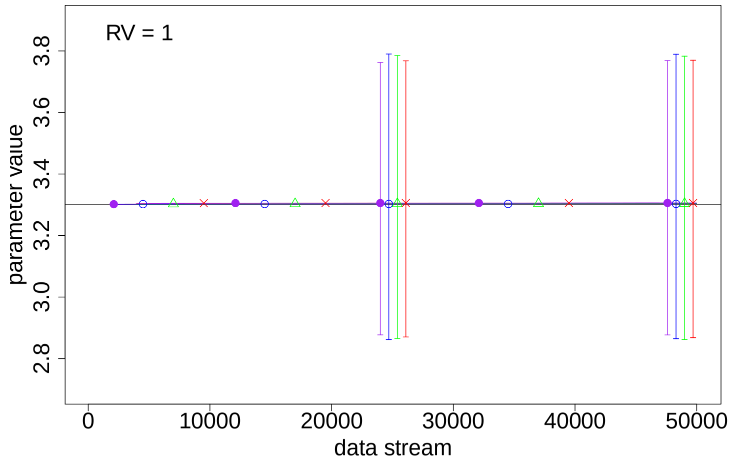

(a) Condition A

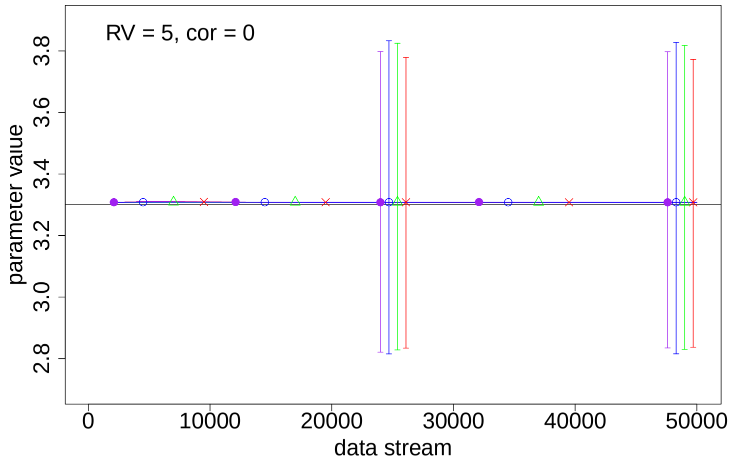

(b) Condition B

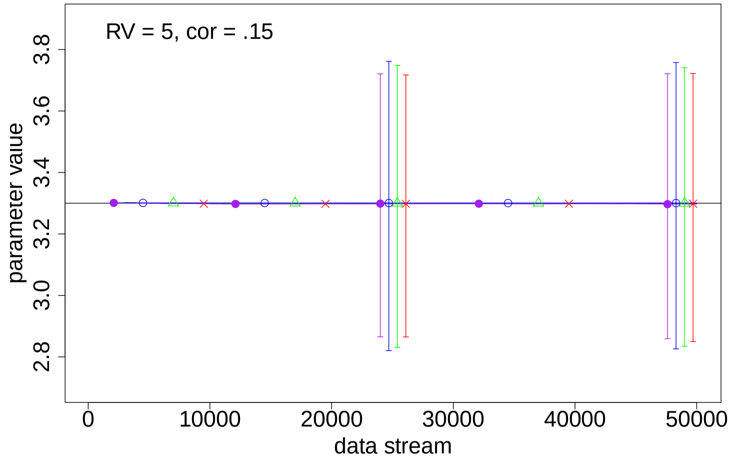

(c) Condition C

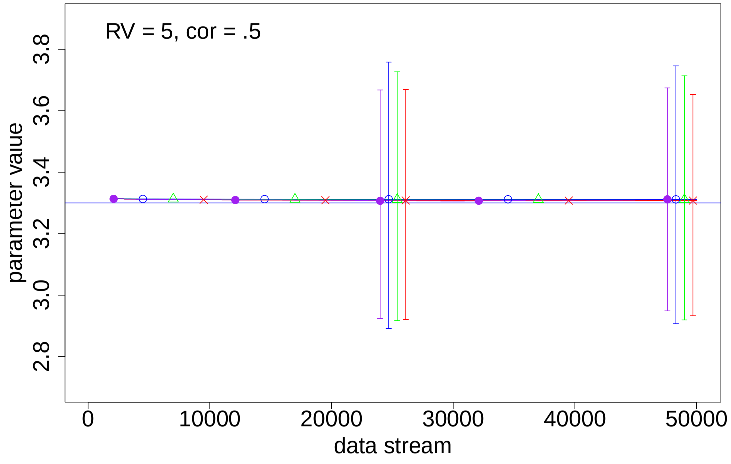

(d) Condition D

Figure (11) Estimated fixed coefficient of level 2 covariate. The true value is 3.3. The error bars indicate the 95% empirical interval of the 1000 simulation runs. The blue line with open circle is SEMA; the green line with triangle is SEMA Update; the red line with‘ ×’is EM; and the purple line with solid circle is Sliding Window EM.

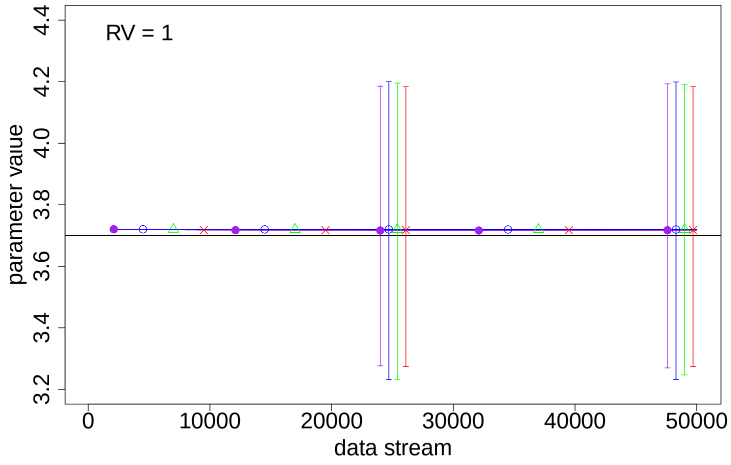

(a) Condition A

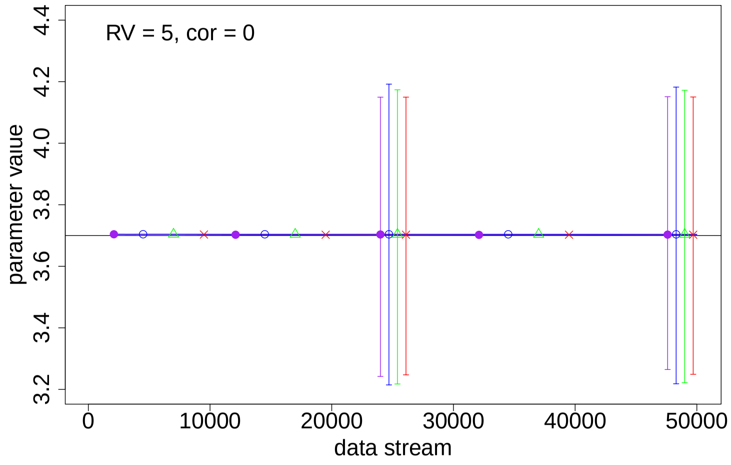

(b) Condition B

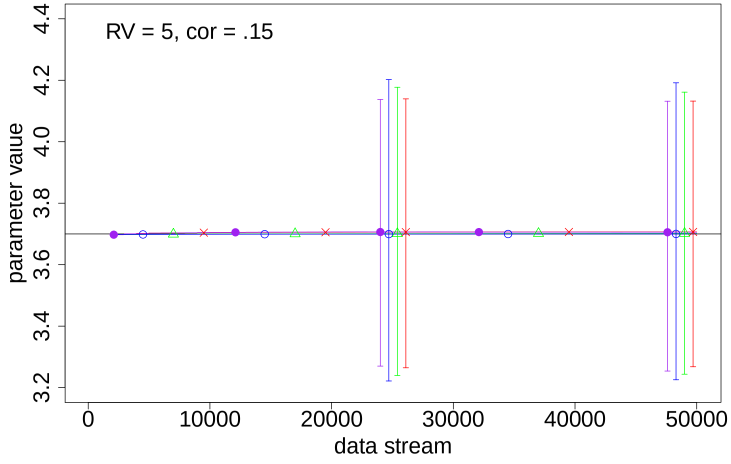

(c) Condition C

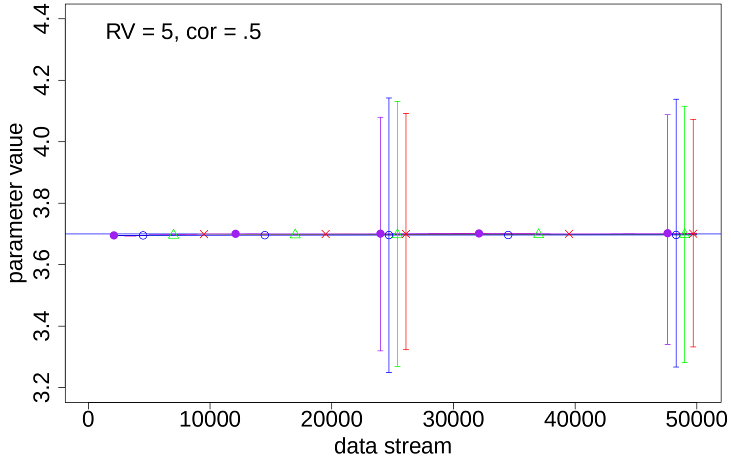

(d) Condition D

Figure (12) Estimated fixed coefficient of level 2 covariate. The true value is 3.7. The error bars indicate the 95% empirical interval of the 1000 simulation runs. The blue line with open circle is SEMA; the green line with triangle is SEMA Update; the red line with‘ × ’is EM; and the purple line with solid circle is Sliding Window EM.

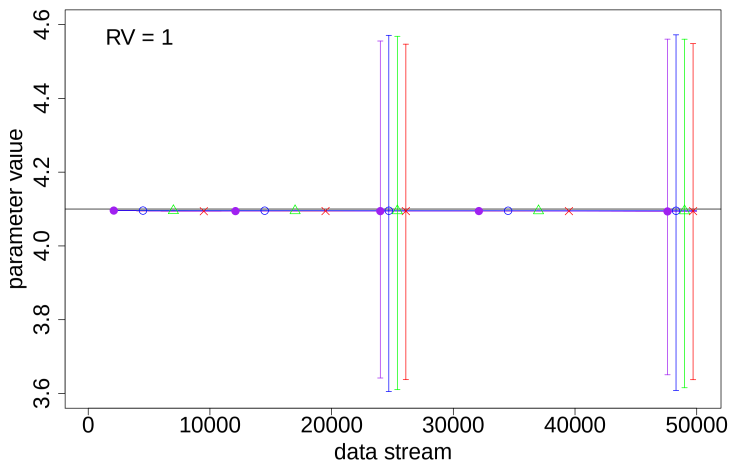

(a) Condition A

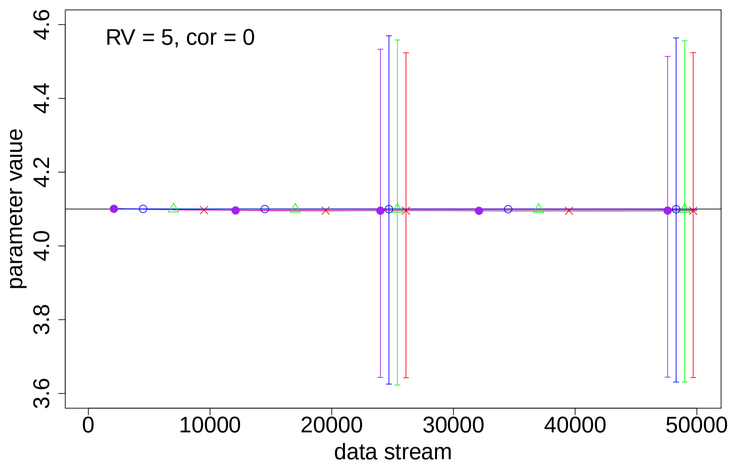

(b) Condition B

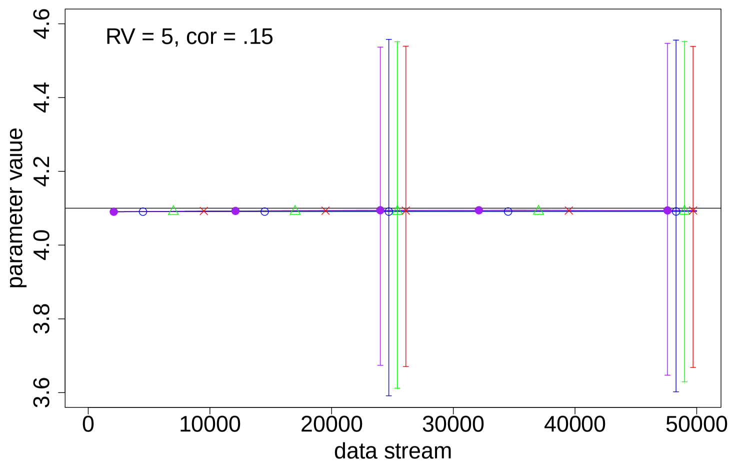

(c) Condition C

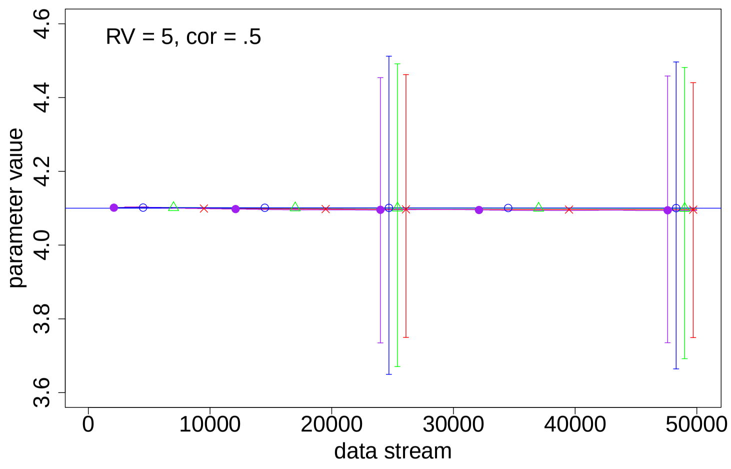

(d) Condition D

Figure (13) Estimated fixed coefficient of level 2 categorical variable, with 2 categories, i.e. a single dummy variable. The true value is 4.1. The error bars indicate the 95% empirical interval of the 1000 simulation runs. The blue line with open circle is SEMA; the green line with triangle is SEMA Update; the red line with'×'is EM; and the purple line with solid circle is Sliding Window EM.

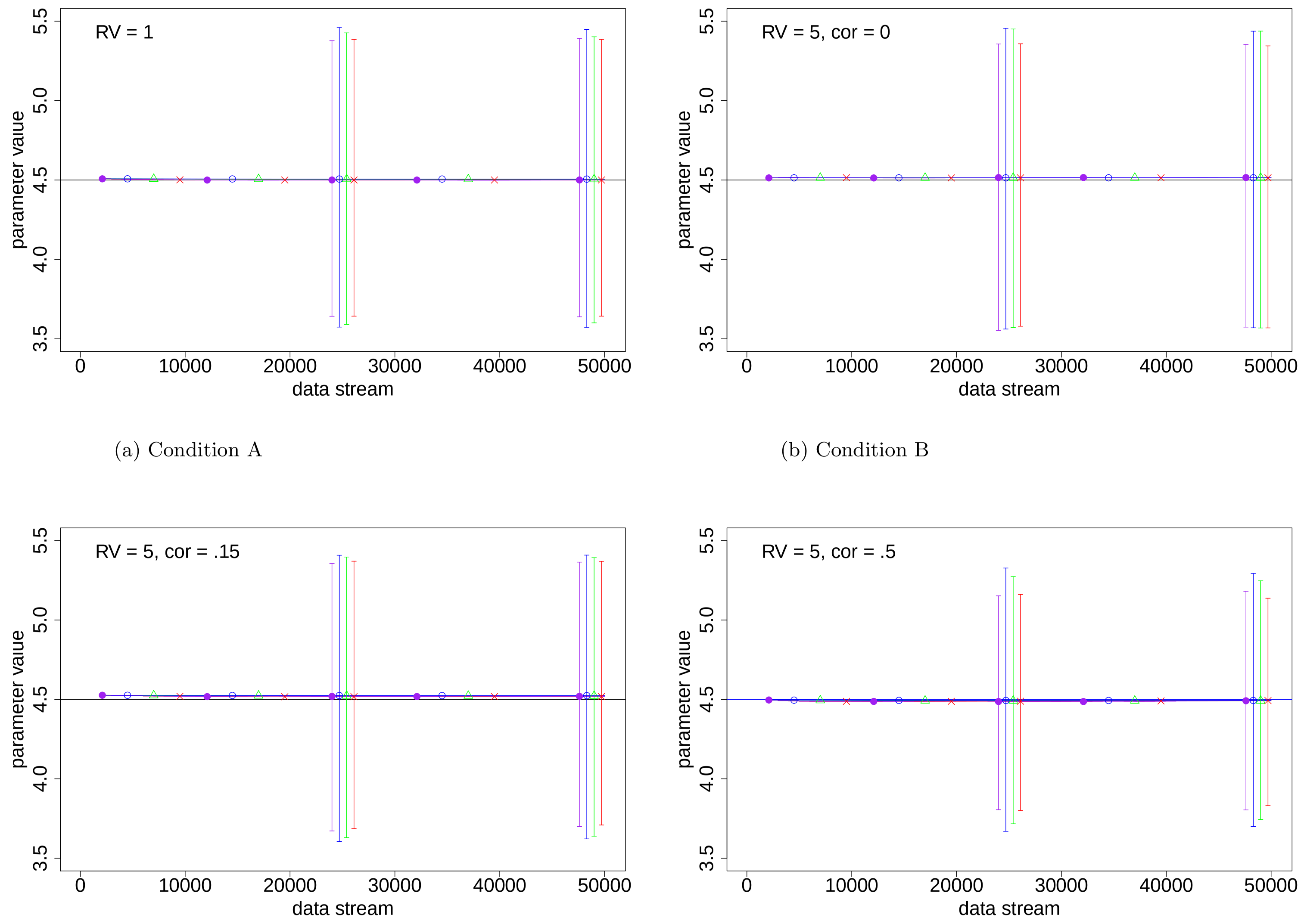

(a) Condition A

(b) Condition B

(c) Condition C

(d) Condition D

Figure (14) Estimated fixed coefficient of level 2 categorical variable, with 3 categories, this is the first dummy variable. The true value is 4.5. The error bars indicate the 95% empirical interval of the 1000 simulation runs. The blue line with open circle is SEMA; the green line with triangle is SEMA Update; the red line with'×'is EM; and the purple line with solid circle is Sliding Window EM.

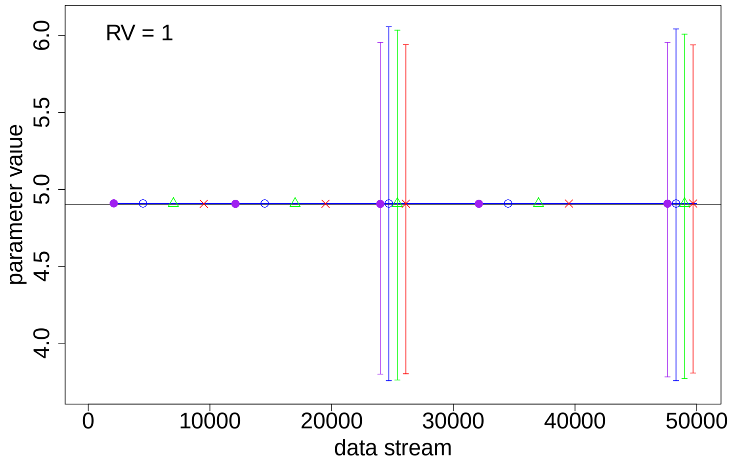

(a) Condition A

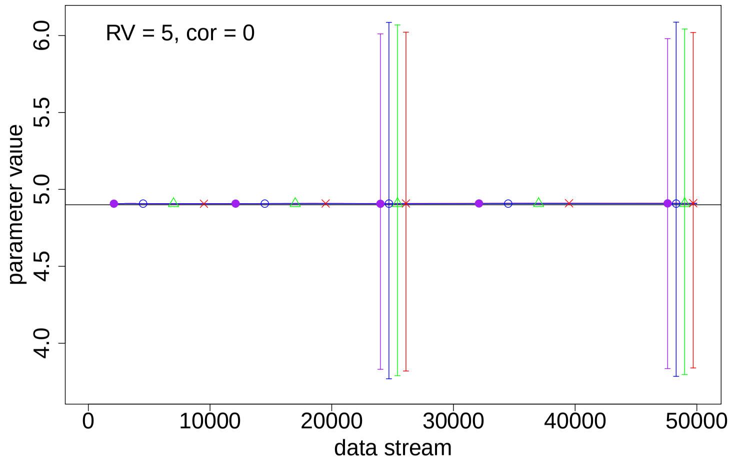

(b) Condition B

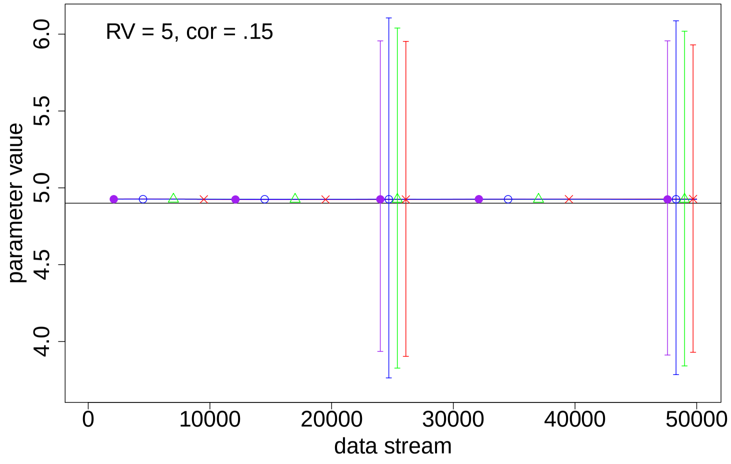

(c) Condition C

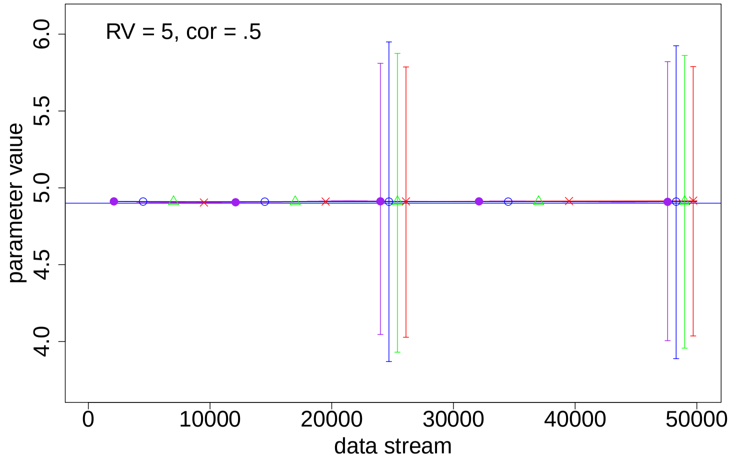

(d) Condition D

Figure (15) Estimated fixed coefficient of level 2 categorical variable, with 3 categories, this is the second dummy variable. The true value is 4.9. The error bars indicate the 95% empirical interval of the 1000 simulation runs. The blue line with open circle is SEMA; the green line with triangle is SEMA Update; the red line with‘×’is EM; and the purple line with solid circle is Sliding Window EM.

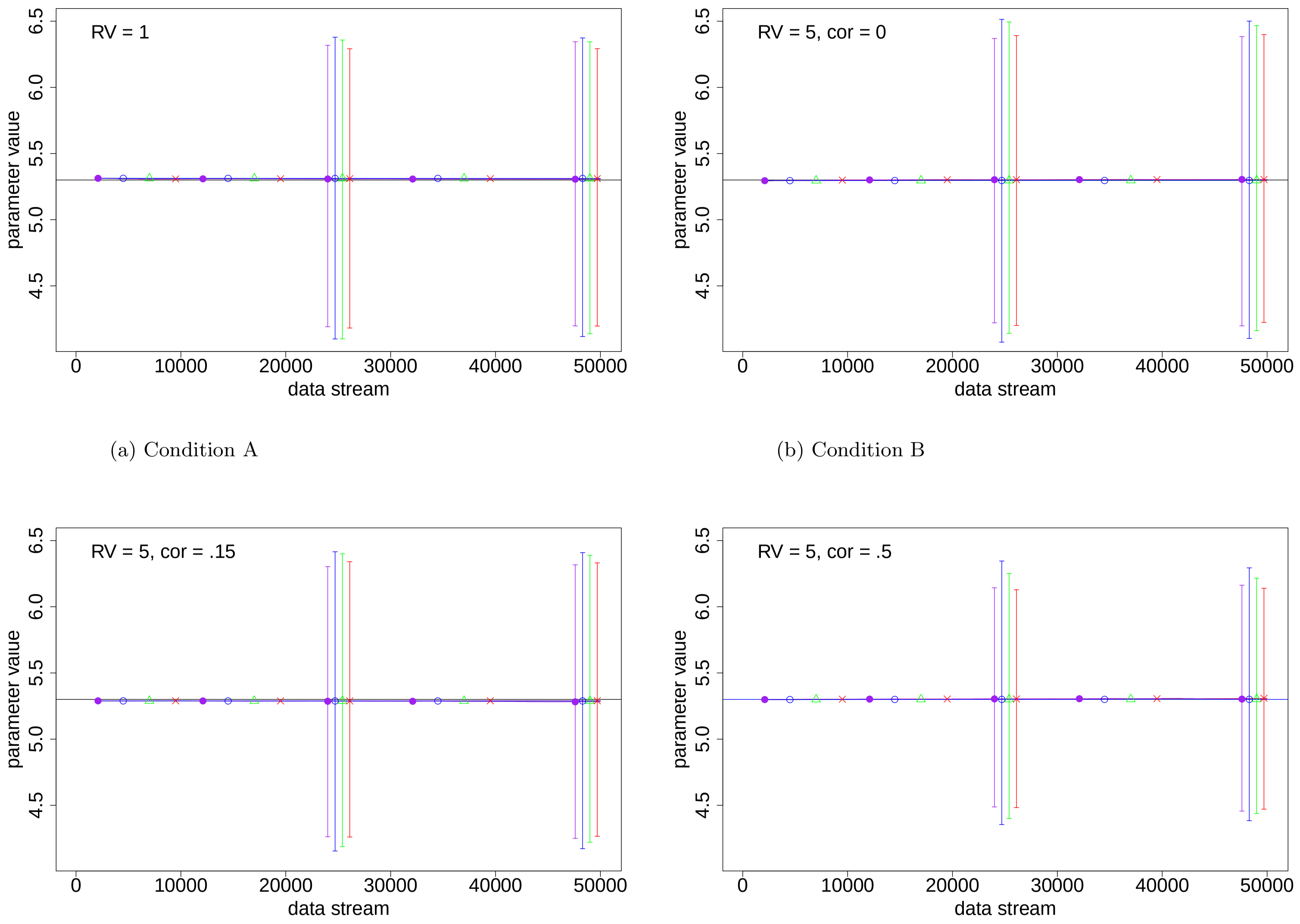

(a) Condition A

(b) Condition B

(c) Condition C

(d) Condition D

Figure (16) Estimated fixed coefficient of level 2 categorical variable, with 3 categories, this is the third dummy variable. The true value is 5.3. The error bars indicate the 95% empirical interval of the 1000 simulation runs. The blue line with open circle is SEMA; the green line with triangle is SEMA Update; the red line with‘×’is EM; and the purple line with solid circle is Sliding Window EM.

2.2 Random effects

The following figures are the results of the random effects. The random effects consist of 5 variances of the random effects and 10 correlations between the random effects. We start with the presentation of the variances (Fig. 16 - 20), followed by the figures containing the results of the correlations (Fig. 21 - 50). Contrary to the figures of the fixed effects and variances, we present the correlations between the random effects per condition. Hence Fig. 21 - 30 present the results of Condition A. The correlations from Condition B are illustrated in Fig. 31 - 40 and the last 10 figures of the random effects belong to Condition D. For each figure, you find the condition noted in the upper left corner and the estimated parameter in the caption of the figure. The methods are again indicated using the same color/symbol combination.

In these figures, you can clearly see the updating scheme of the different methods: while the SWEM and EM methods clearly show steps from update to update, the SEMA update only shows smooth change in between the updates (where it shows more profound changes in parameter estimates), and lastly, SEMA has a smooth curve retrieving the data generating parameters slightly later than the previous methods.

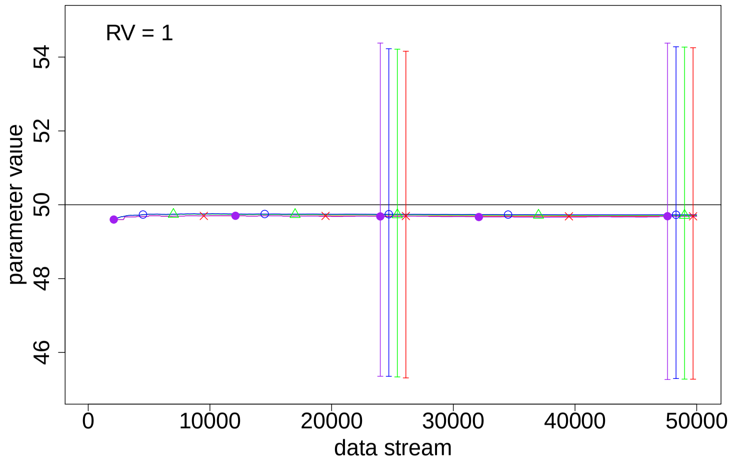

(a) Condition A

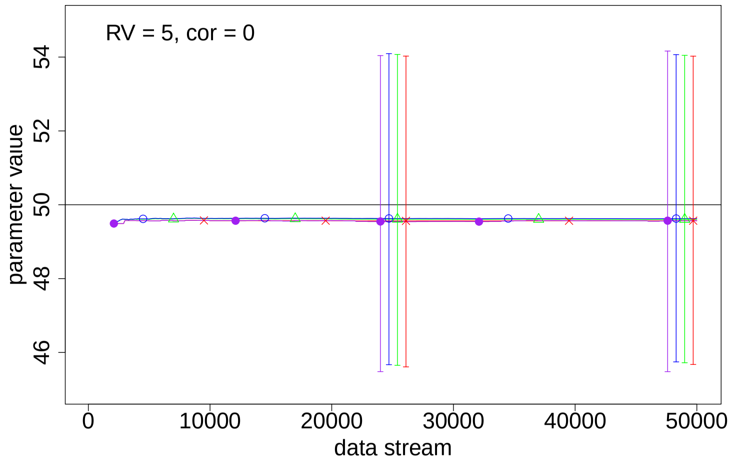

(b) Condition B

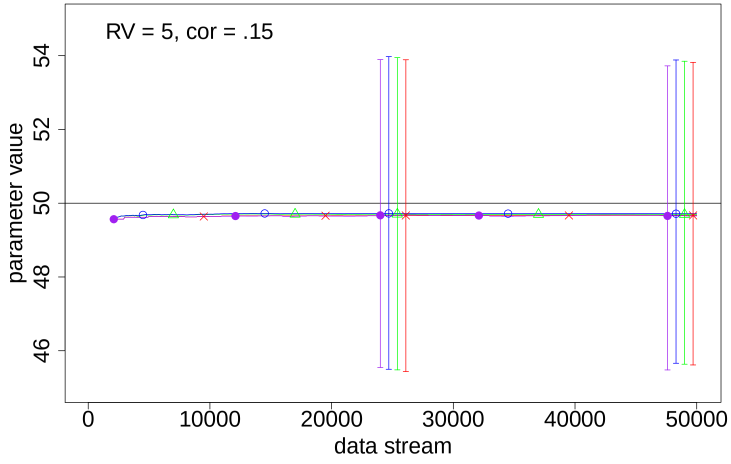

(c) Condition C

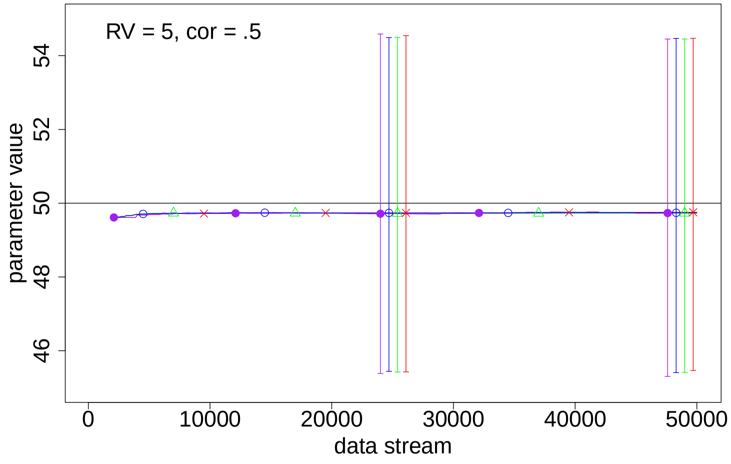

(d) Condition D

Figure (17) Estimated variance of the intercept. The true value is equal to 50. The error bars indicate the 95% empirical interval of the 1000 simulation runs. The blue line with open circle is SEMA; the green line with triangle is SEMA Update; the red line with'×'is EM; and the purple line with solid circle is Sliding Window EM.

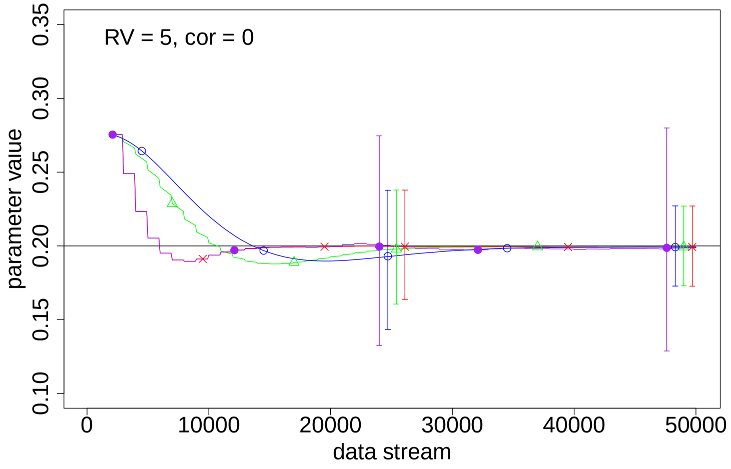

(a) Condition B

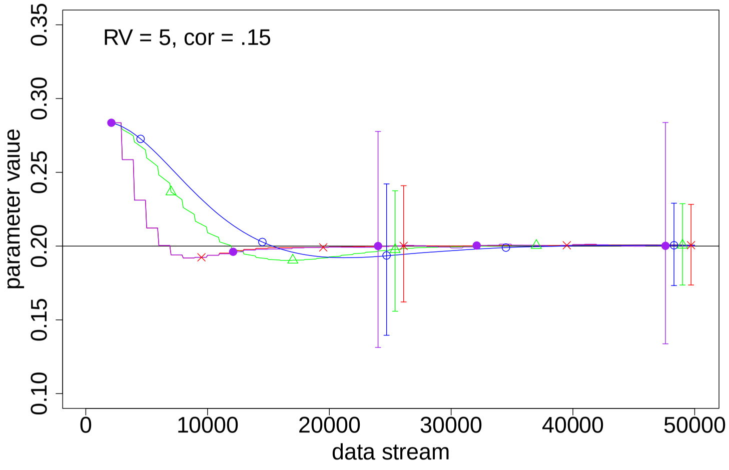

(b) Condition C

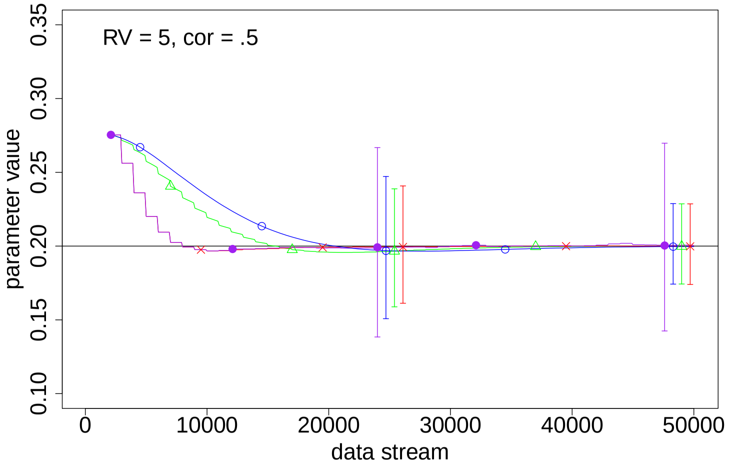

(c) Condition D

Figure (18) Estimated random slope. The true value is .2. The error bars indicate the 95% empirical interval of the 1000 simulation runs. The blue line with open circle is SEMA; the green line with triangle is SEMA Update; the red line with‘×’is EM; and the purple line with solid circle is Sliding Window EM.

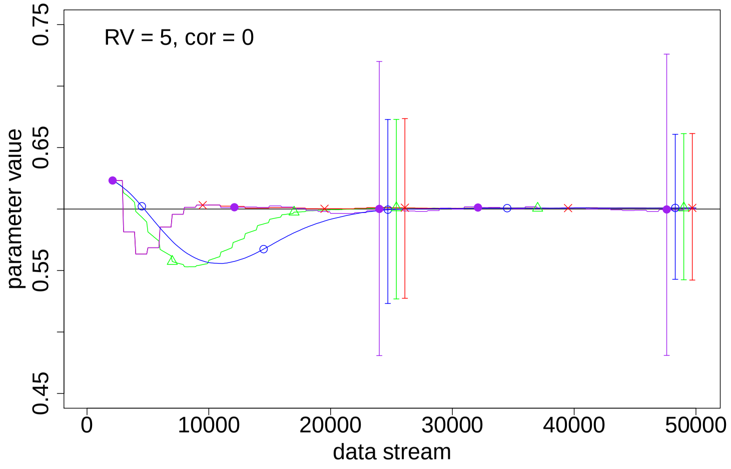

(a) Condition B

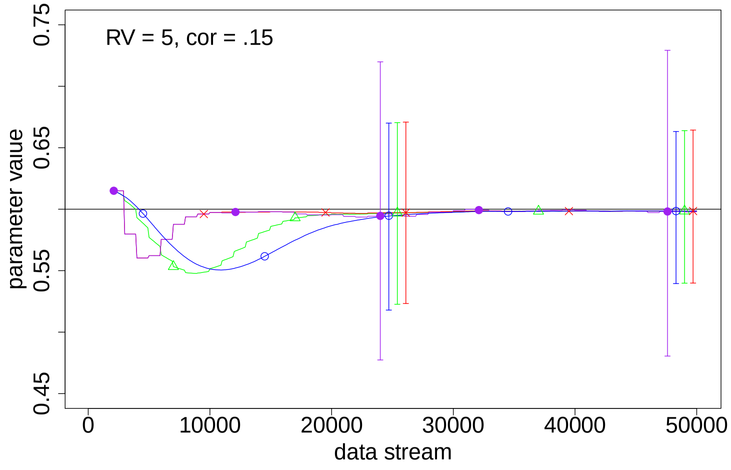

(b) Condition C

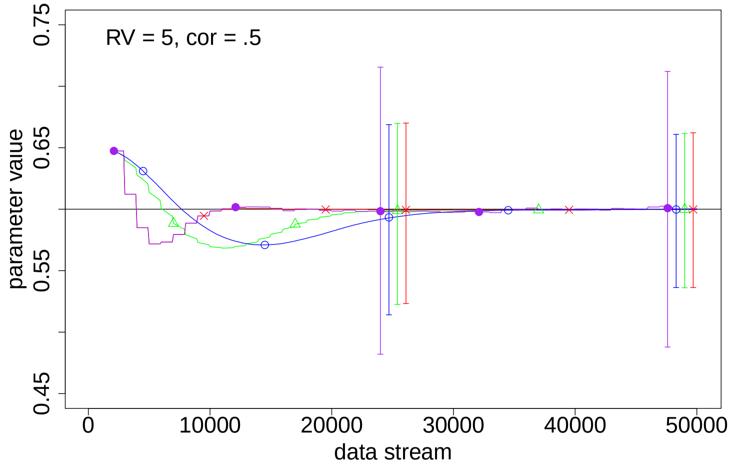

(c) Condition D

Figure (19) Estimated random slope. The true value is equal to .6. The error bars indicate the 95% empirical interval of the 1000 simulation runs. The blue line with open circle is SEMA; the green line with triangle is SEMA Update; the red line with'×'is EM; and the purple line with solid circle is Sliding Window EM.

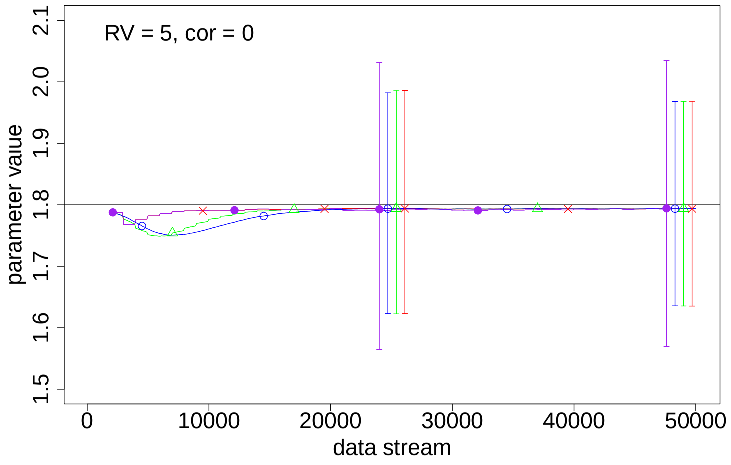

(a) Condition B

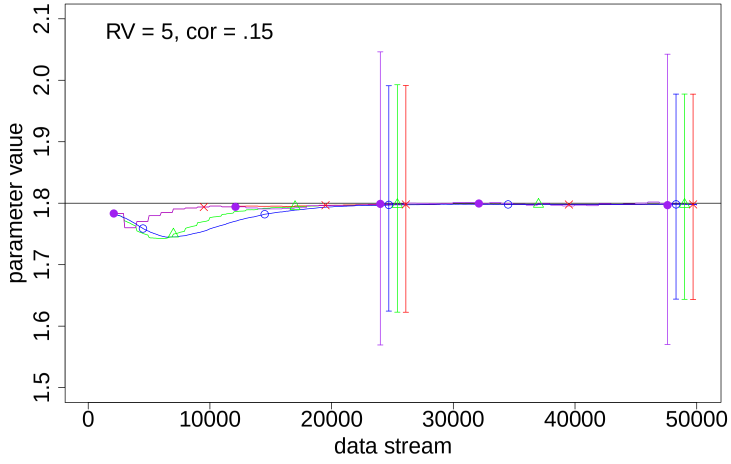

(b) Condition C

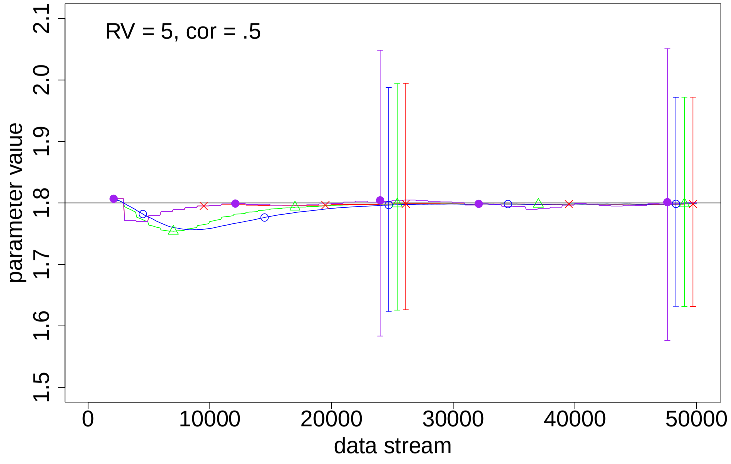

(c) Condition D

Figure (20) Estimated random slope. The true value is equal to 1.8. The error bars indicate the 95% empirical interval of the 1000 simulation runs. The blue line with open circle is SEMA; the green line with triangle is SEMA Update; the red line with'×'is EM; and the purple line with solid circle is Sliding Window EM.

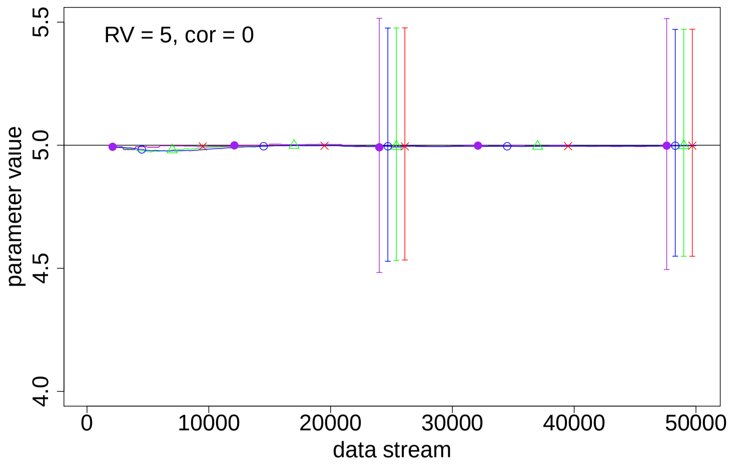

(a) Condition B

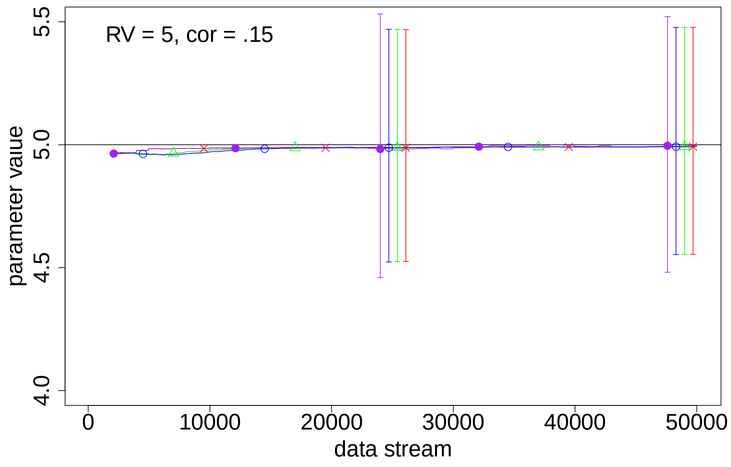

(b) Condition C

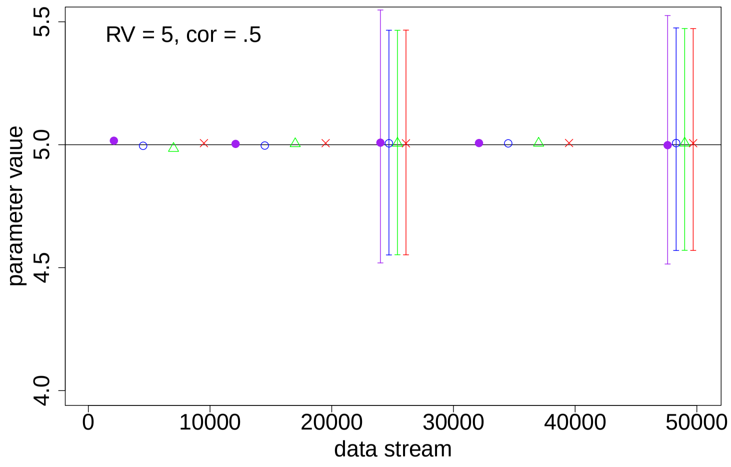

(c) Condition D

Figure (21) Estimated random slope. The true value is equal to 5. The error bars indicate the 95% empirical interval of the 1000 simulation runs. The blue line with open circle is SEMA; the green line with triangle is SEMA Update; the red line with‘×’is EM; and the purple line with solid circle is Sliding Window EM.

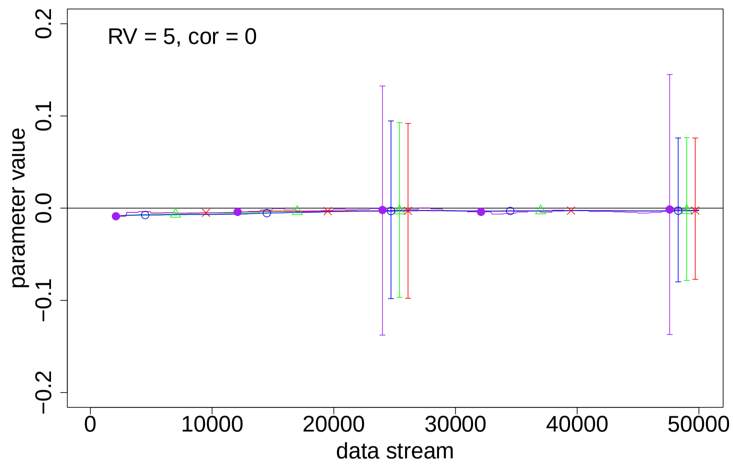

Figure (22) Correlation between intercept and first random slope

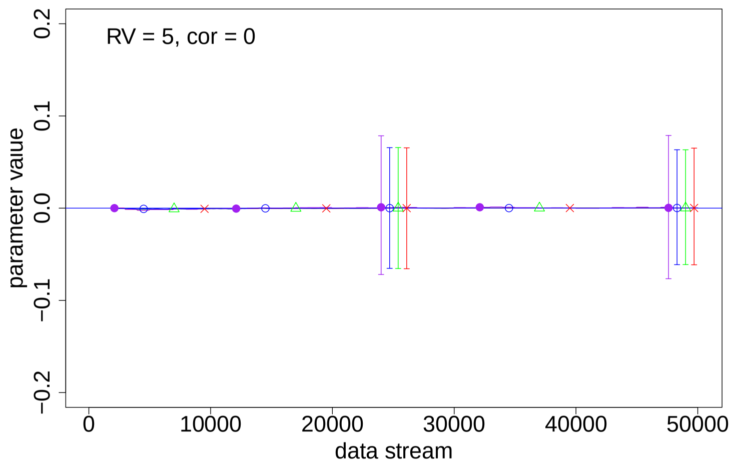

Figure (24) Correlation between intercept and third random slope

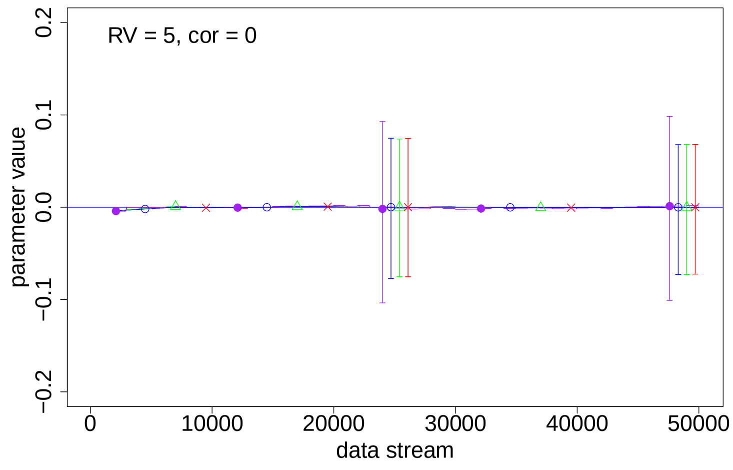

Figure (23) Correlation between intercept and second random slope

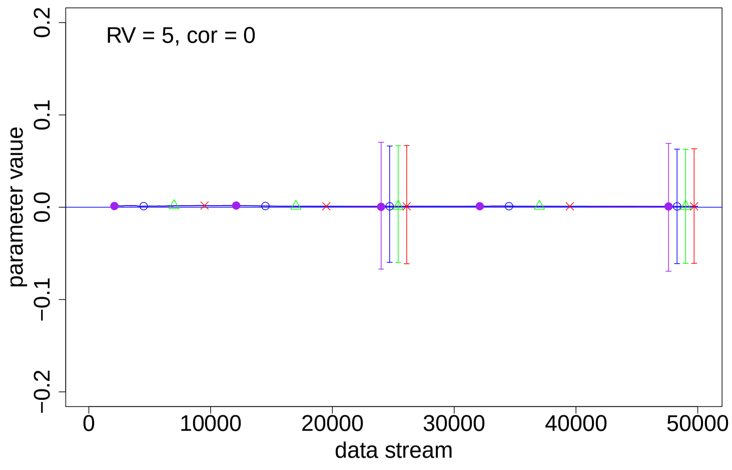

Figure (25) Correlation between intercept and fourth random slope

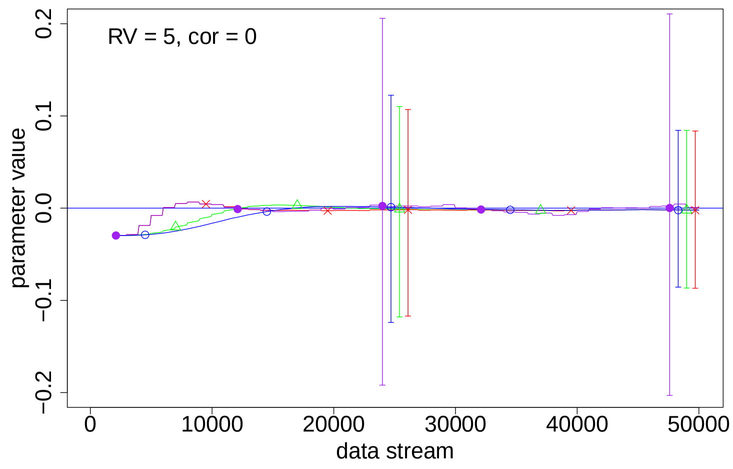

Figure (26) Correlation between first and second random slope

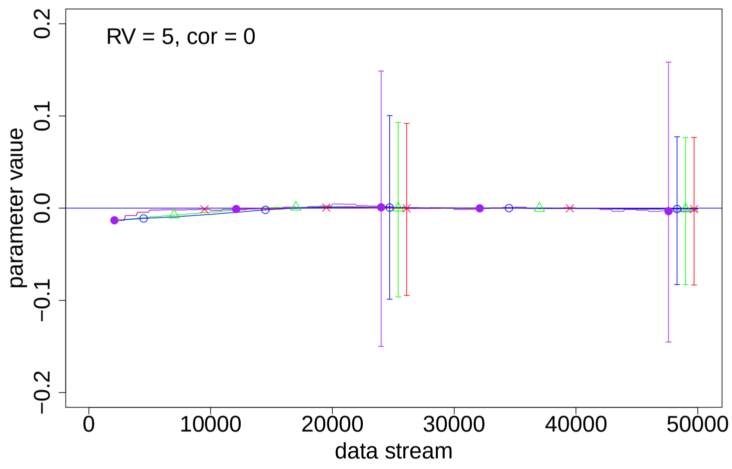

Figure (28) Correlation between first and fourth random slope

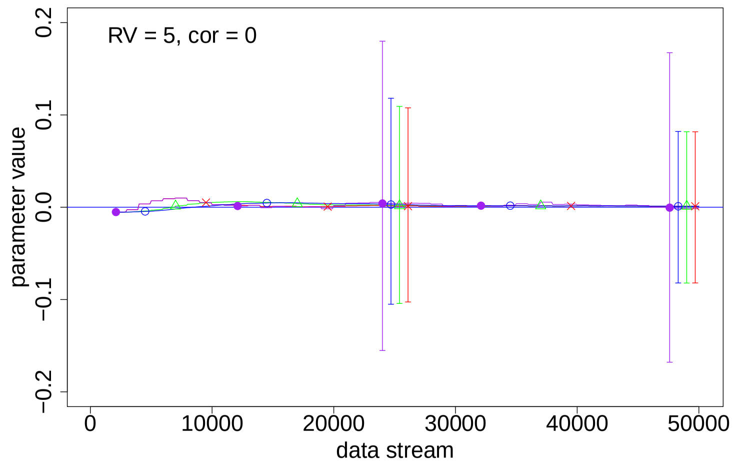

Figure (27) Correlation between first and third random slope

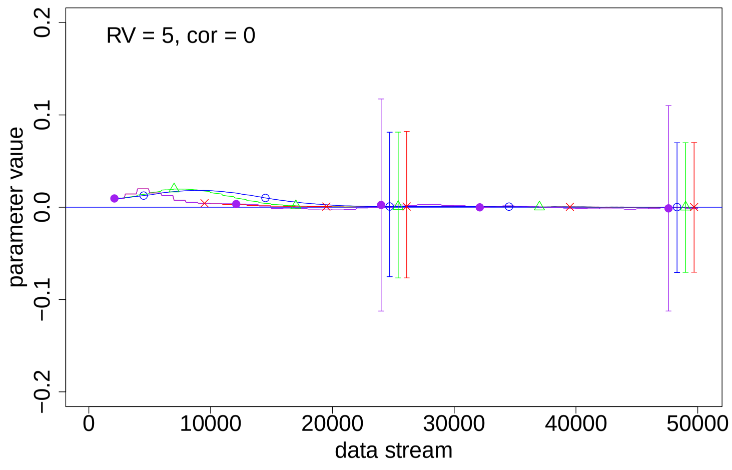

Figure (29) Correlation between second and third random slope

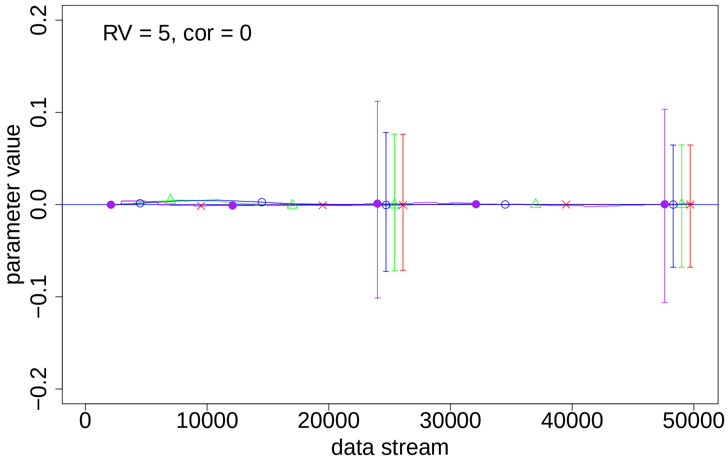

Figure (30) Correlation between second and fourth random slope

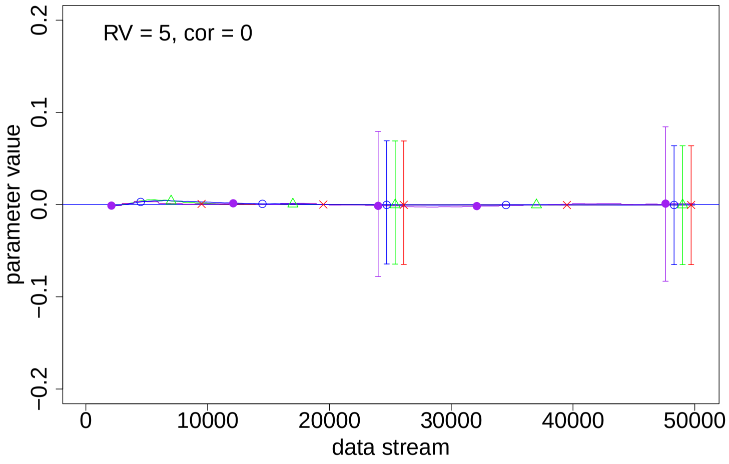

Figure (31) Correlation between third and fourth random slope

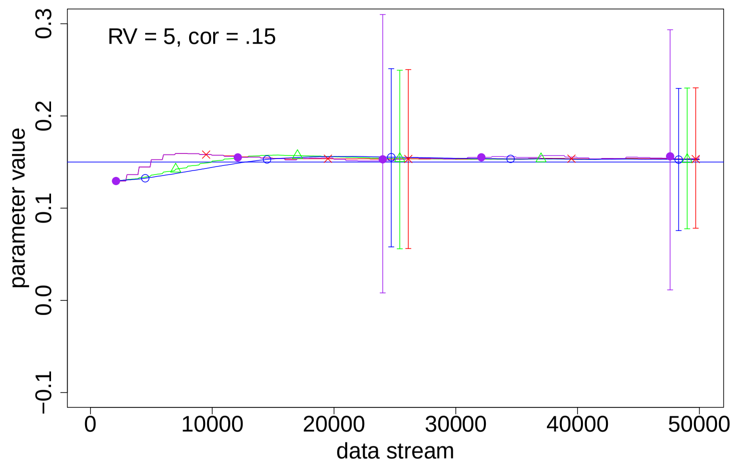

Figure (32) Correlation between intercept and first random slope

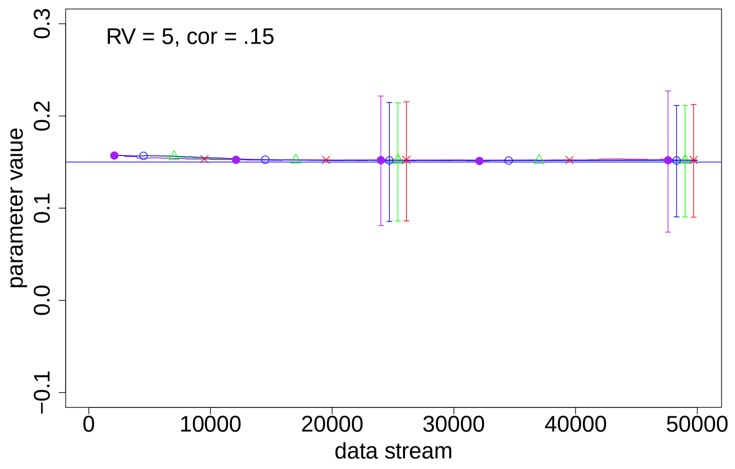

Figure (34) Correlation between intercept and third random slope

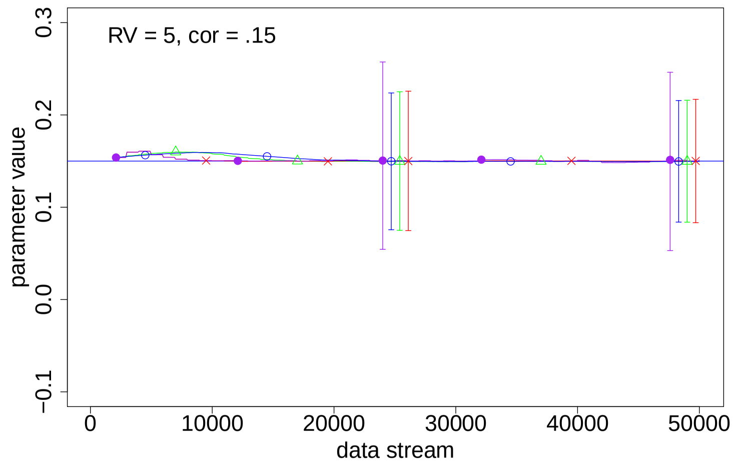

Figure (33) Correlation between intercept and second random slope

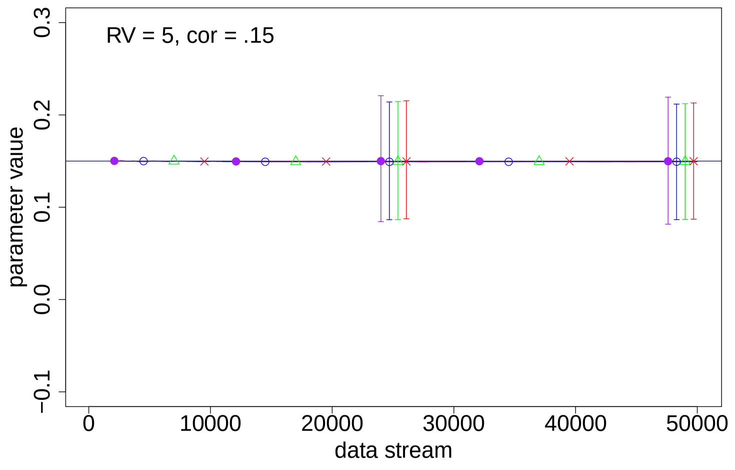

Figure (35) Correlation between intercept and fourth random slope

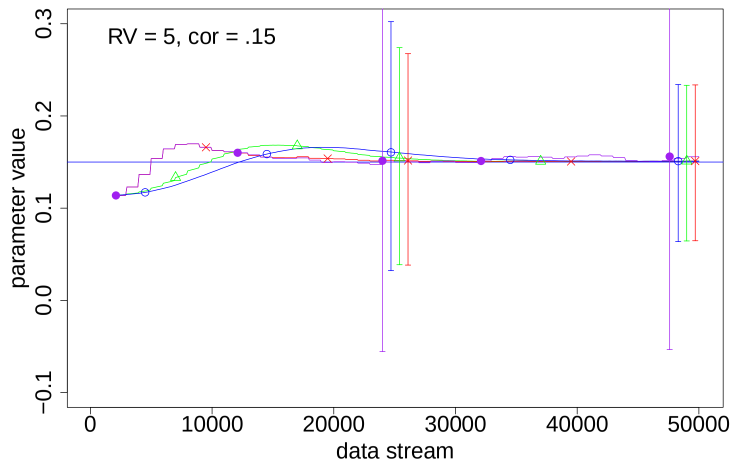

Figure (36)   Correlation between first and second random slope

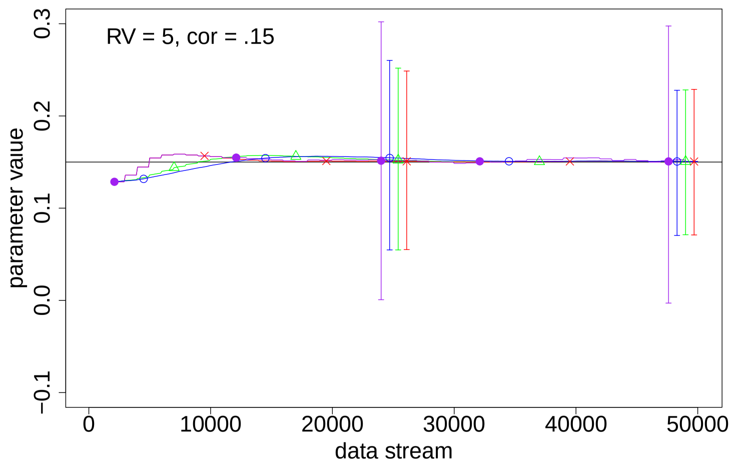

Figure (38)   Correlation between first and fourth random slope

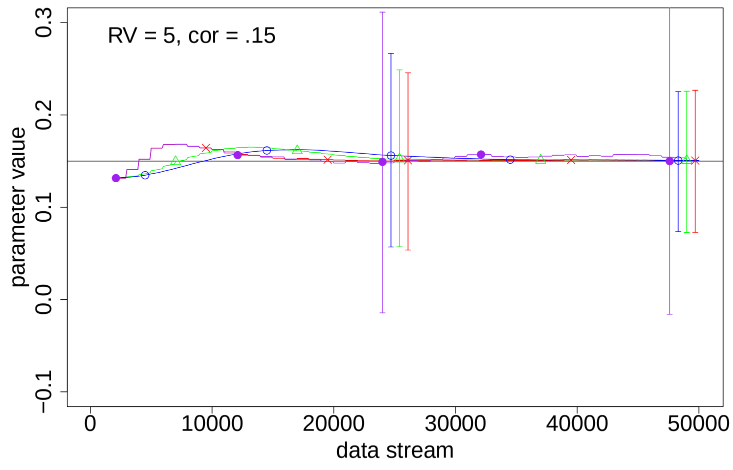

Figure (37)   Correlation between first and third random slope

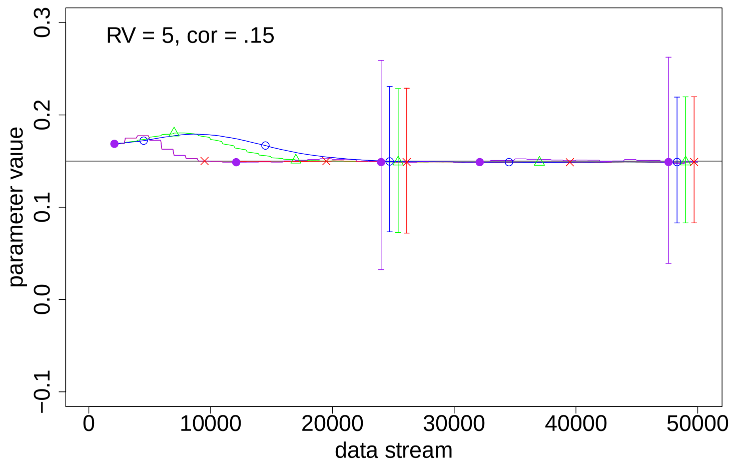

Figure (39)   Correlation between second and third random slope

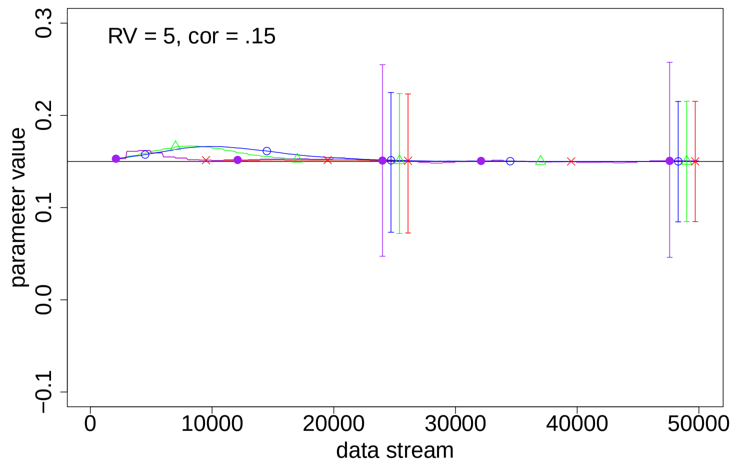

Figure (40) Correlation between third and fourth random slope

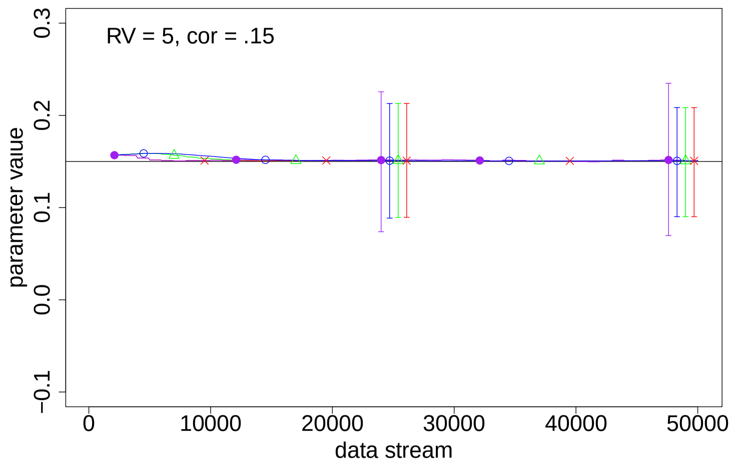

Figure (41) Correlation between third and fourth random slope

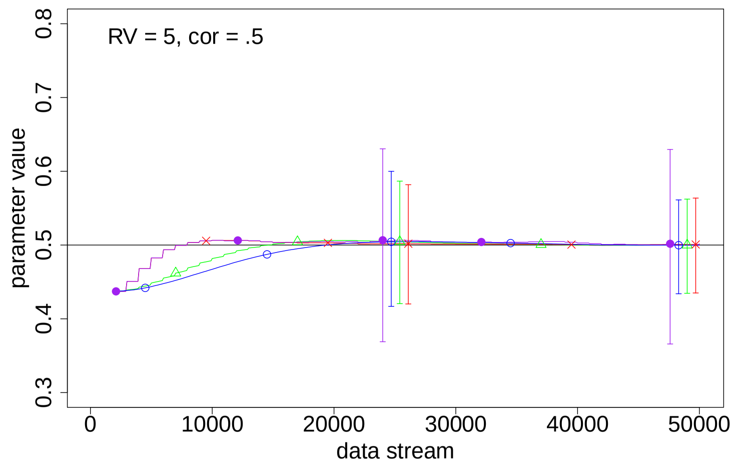

Figure (42) Correlation between intercept and first random slope

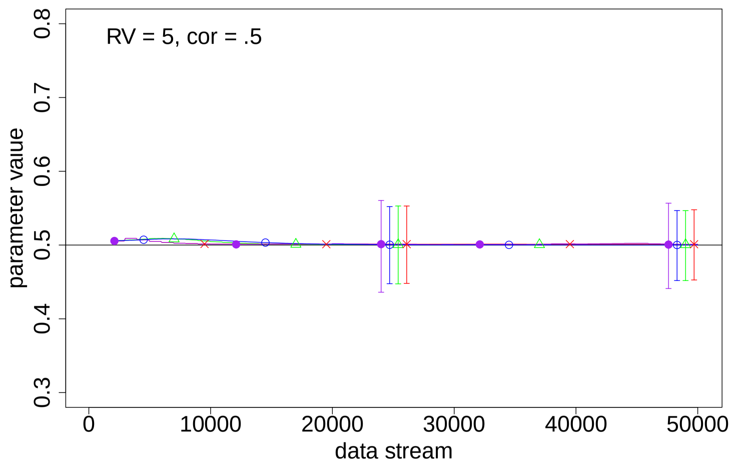

Figure (44) Correlation between intercept and third random slope

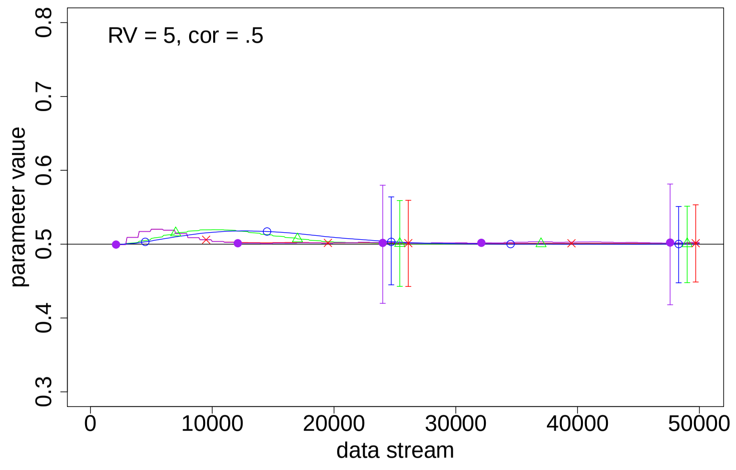

Figure (43) Correlation between intercept and second random slope

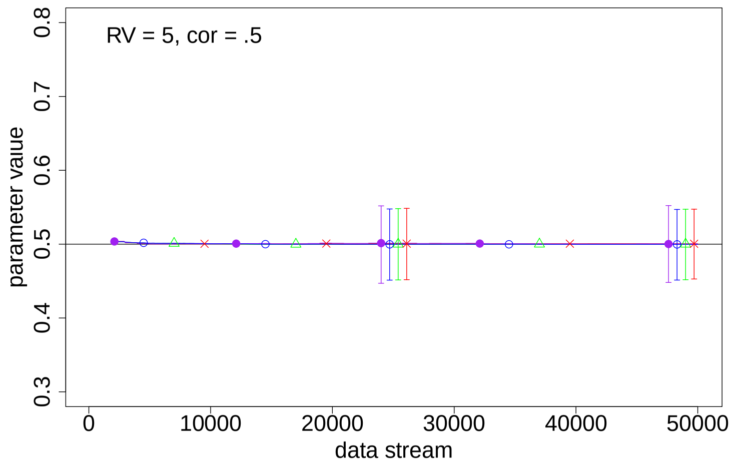

Figure (45) Correlation between intercept and fourth random slope

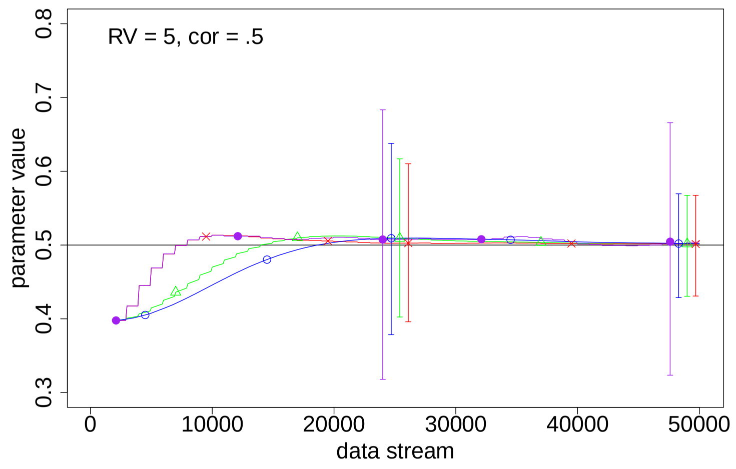

Figure (46) Correlation between first and second random slope

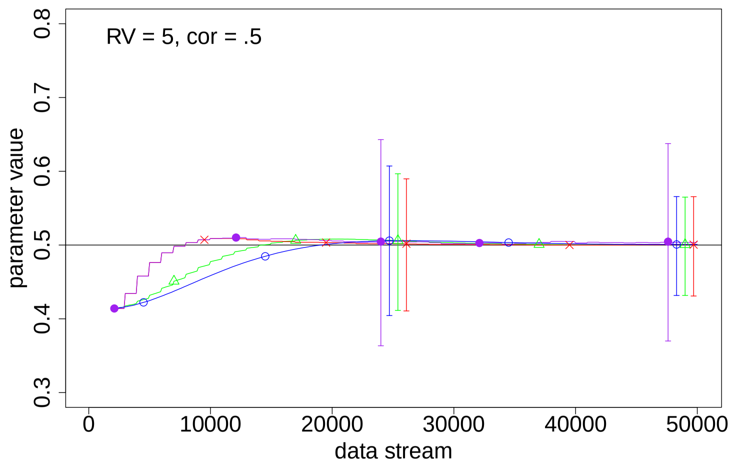

Figure (48) Correlation between first and fourth random slope

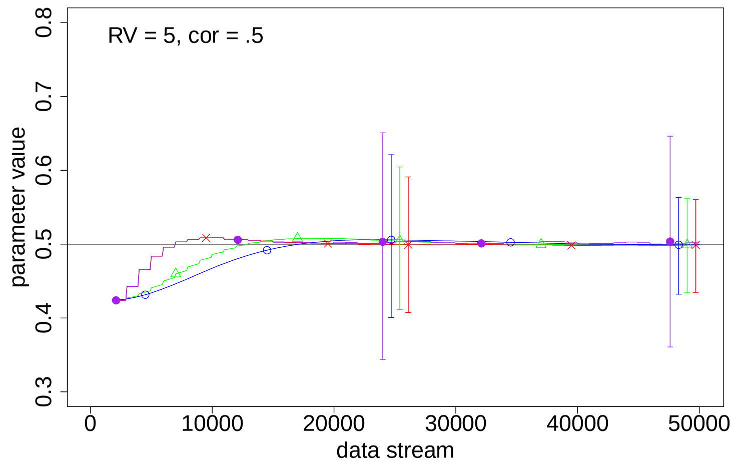

Figure (47) Correlation between first and third random slope

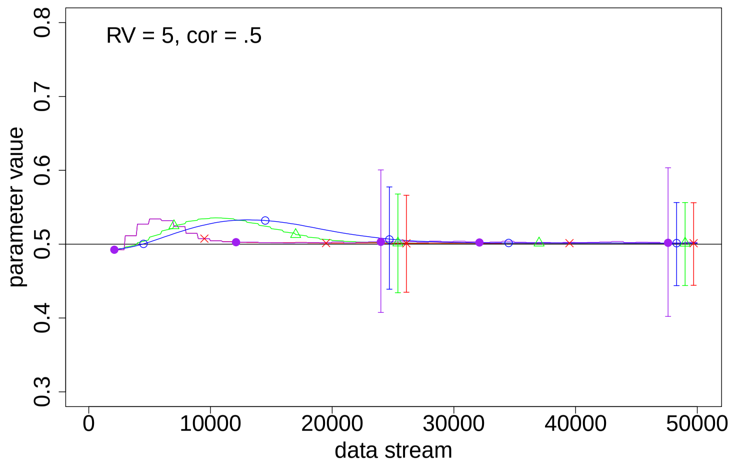

Figure (49) Correlation between second and third random slope

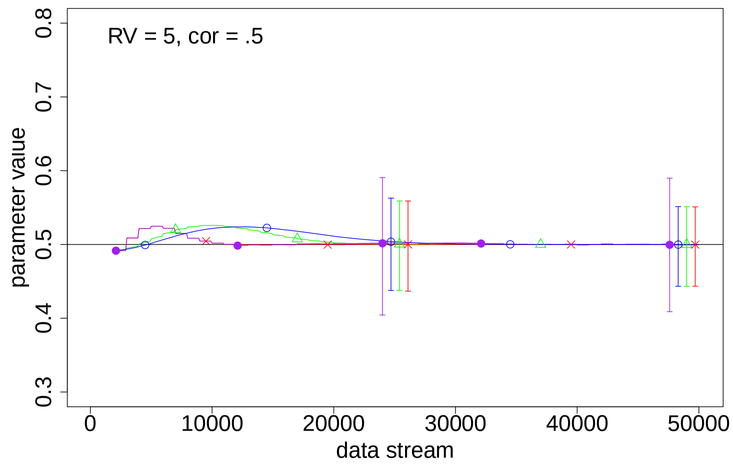

Figure (50) Correlation between third and fourth random slope

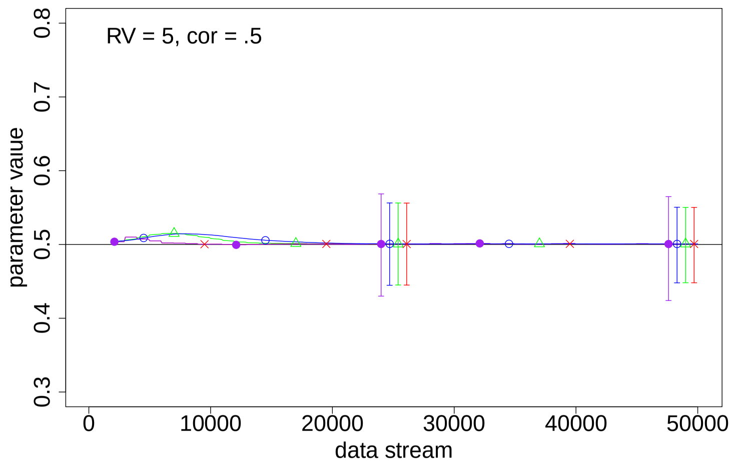

Figure (51) Correlation between third and fourth random slope

3 Results of SEMA in action: fluctuations in weight

This section accompanies the results section of the application presented in the paper. First the results of the fixed effects are presented. The steep change which is visable in the figures is due to retraining when about 300 new individuals were added to the original sample. While the results of EM, SU, and SEMA present similar trends in the effects, SWEM, which only uses about 2 months worth of data, fluctuate much more. The symbols identifying the different methods are the same as in the previous simulation study: blue line with open circle is SEMA, purple line with closed circle is SWEM, the green line with open triangle is SU, the red line with '×' is EM algorithm.

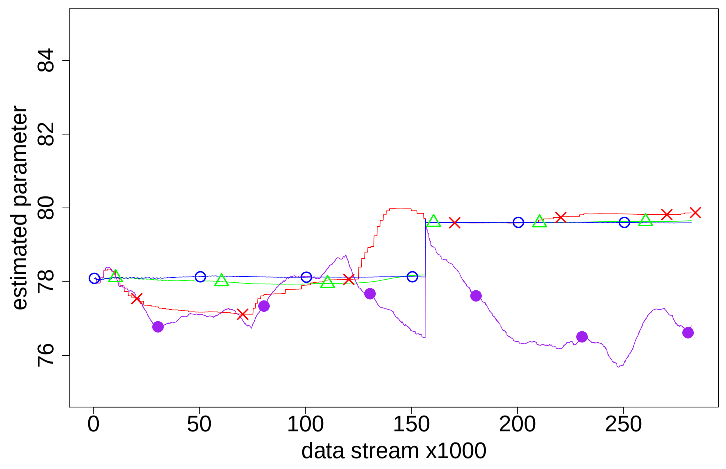

Figure (52) Fixed effect, intercept

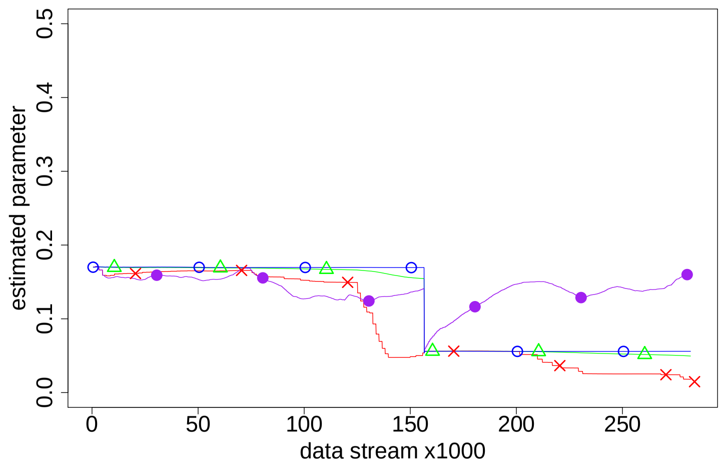

Figure (54) Fixed effect, age(centered)

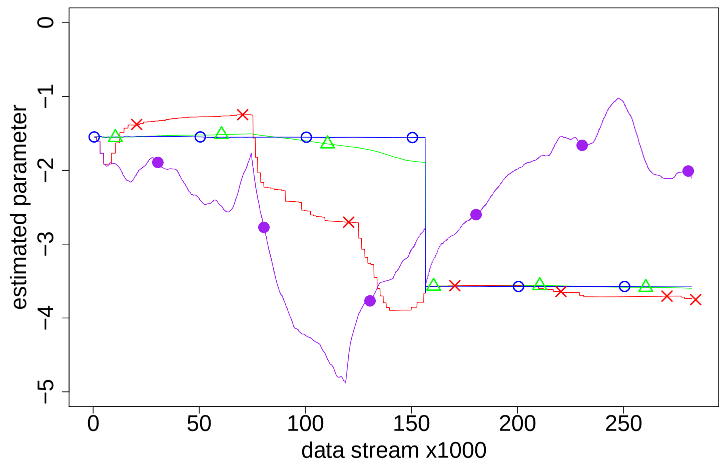

Figure (53) Fixed effect, gender, reference is male

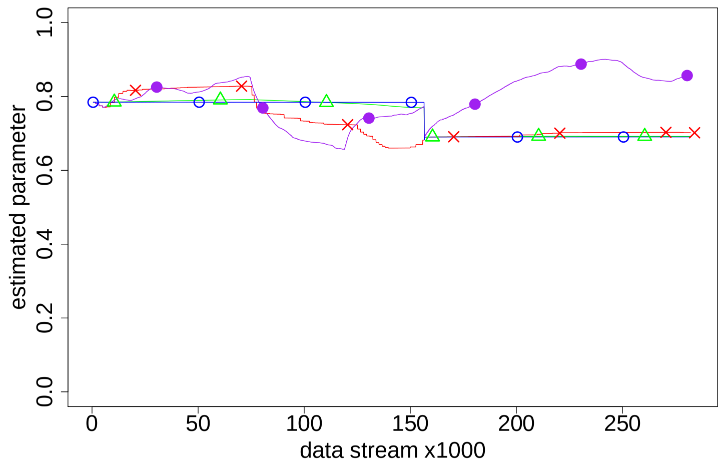

Figure (55) Fixed effect, length (centered)

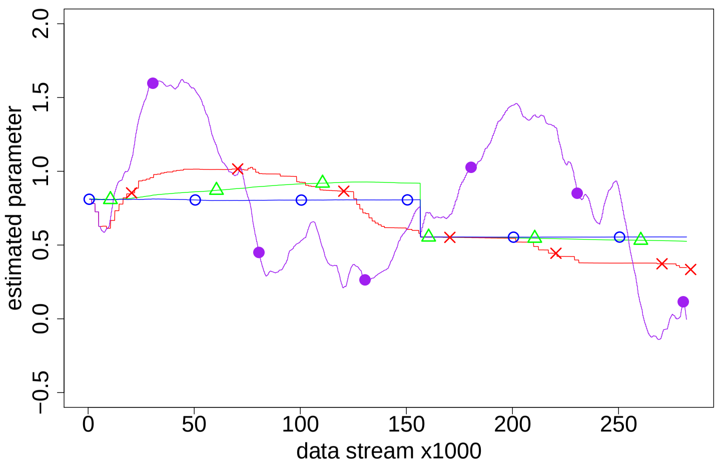

Figure (56) Fixed effect, weigh frequency: daily

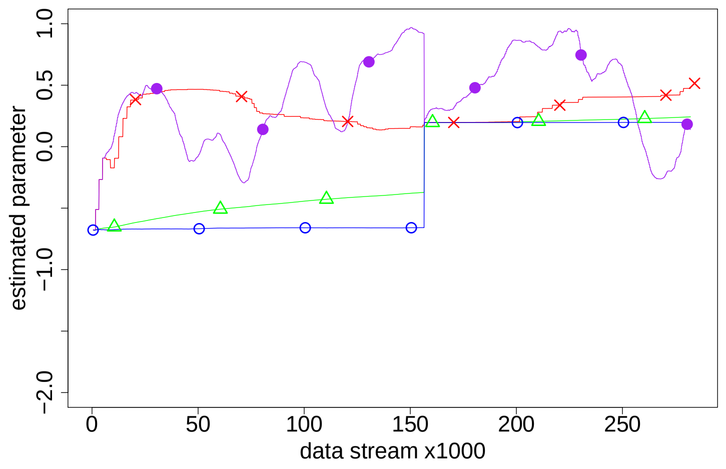

Figure (58) Fixed effect, Feedback type: weight and norm weight

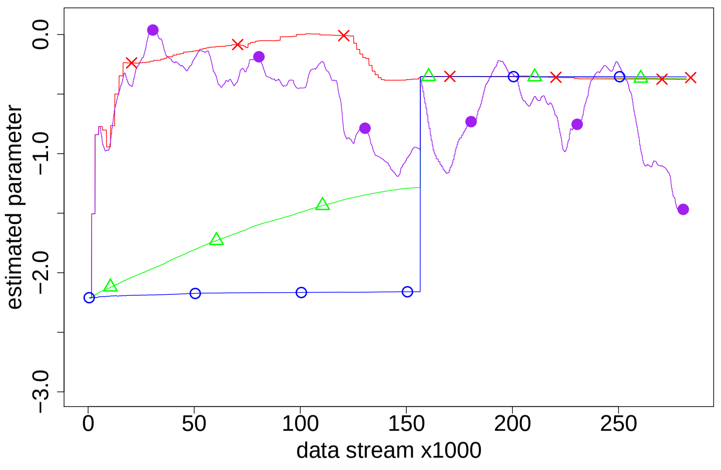

Figure (57) Fixed effect, weigh frequency: weekly

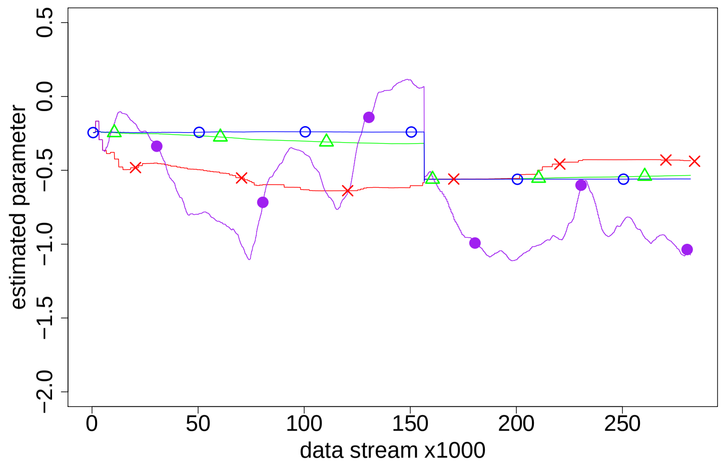

Figure (59) Fixed effect, Feedback type: weight and target weight

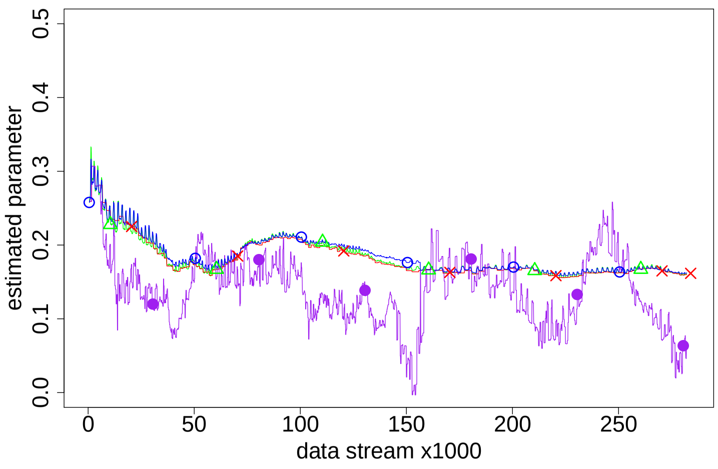

Figure (60) Fixed effect, Sunday compared to Friday

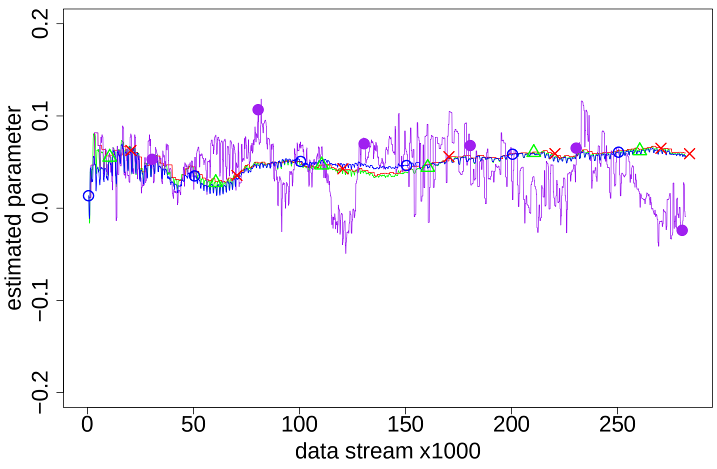

Figure (62) Fixed effect, Wednesday compared to Friday

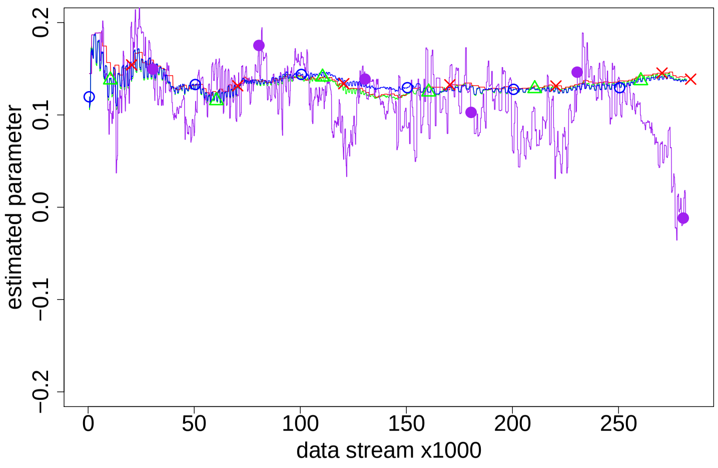

Figure (61) Fixed effect, Tuesday compared to Friday

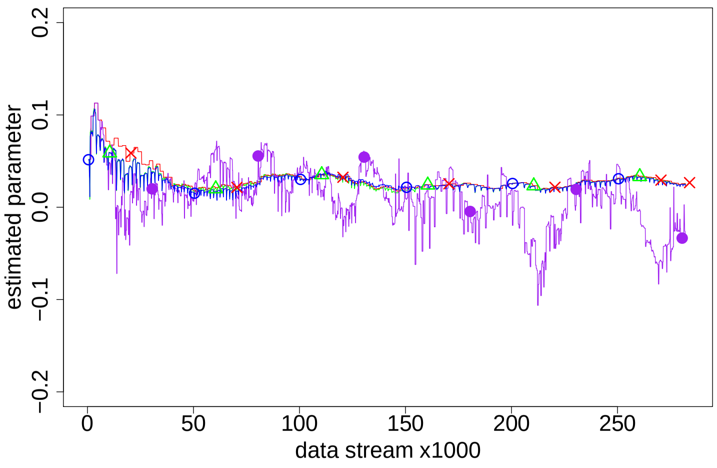

Figure (63) Fixed effect, Thursday compared to Friday

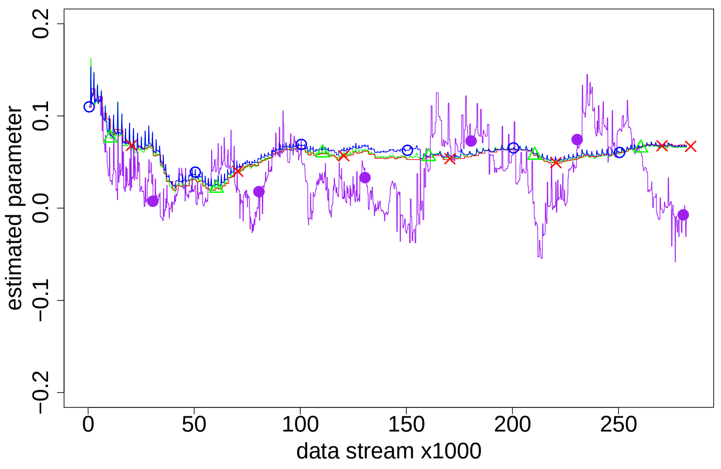

Figure (64) Fixed effect, Saturday compared to Friday

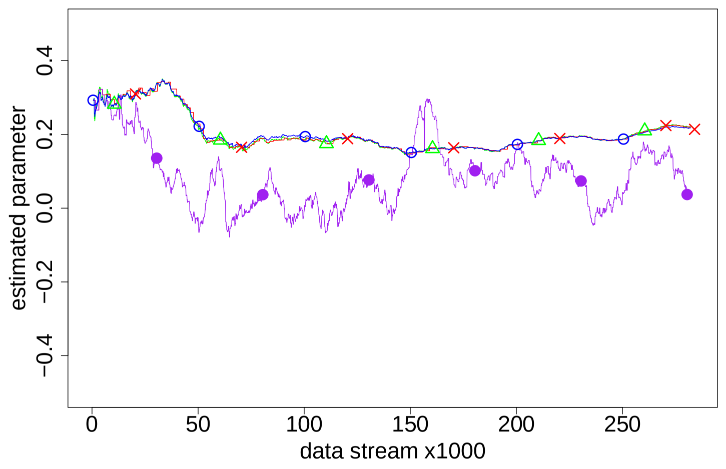

Figure (66) Fixed effect, weigh time afternoon compared to morning

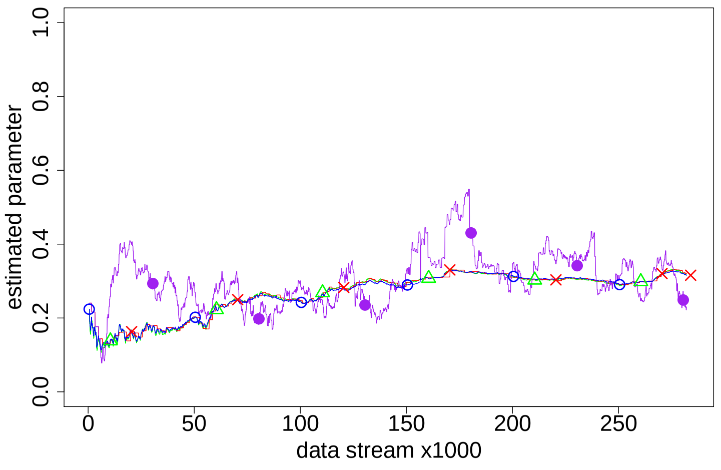

Figure (65) Fixed effect, weigh time night time compared to morning

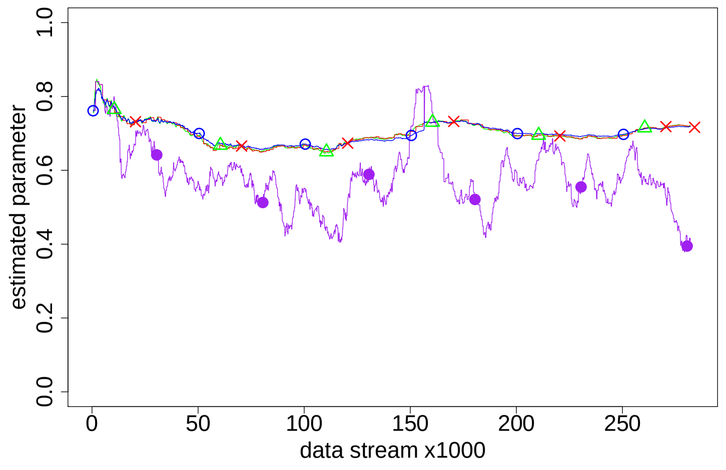

Figure (67) Fixed effect, weigh time evening compared to morning

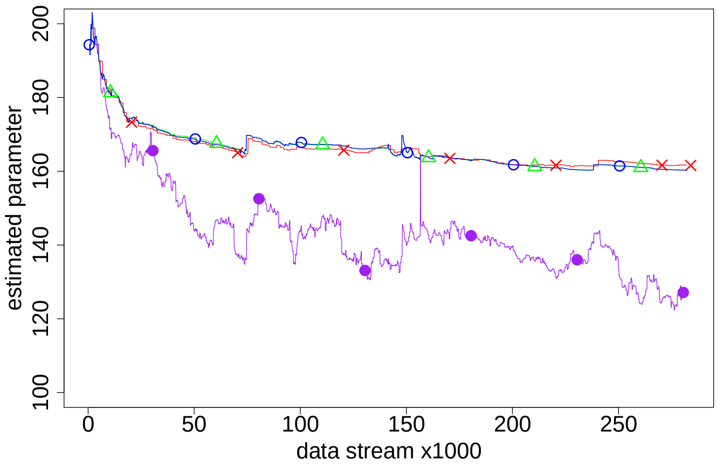

Figure (68)   Variance of the intercept

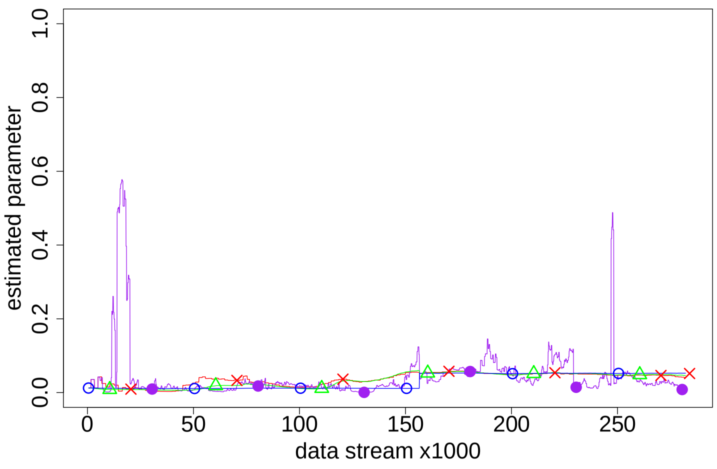

Figure (70)   Variance of Tuesday effect

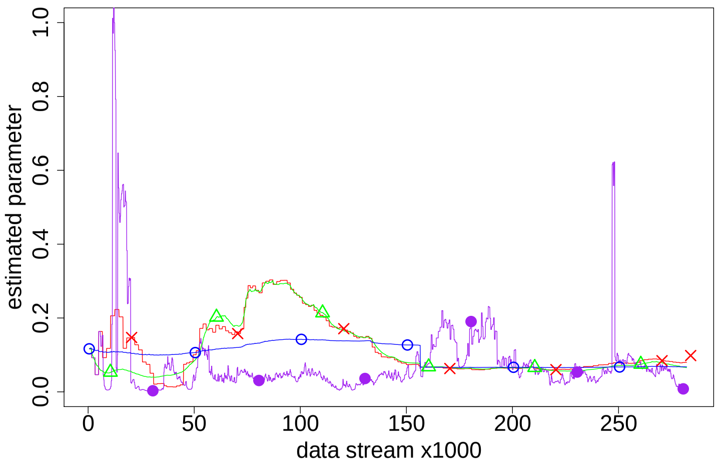

Figure (69)   Variance of Sunday effect

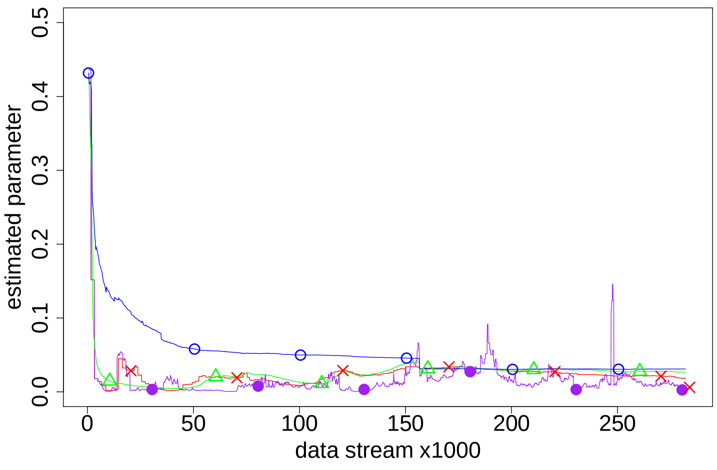

Figure (71)   Variance of Wednesday effect

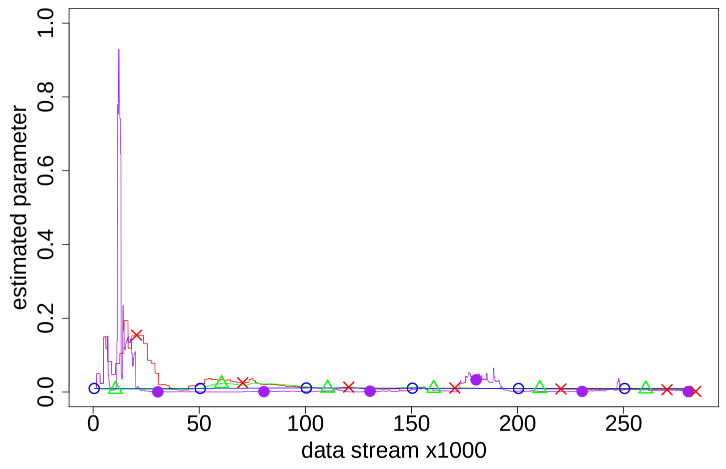

Figure (72) Variance of Thurdayday effect

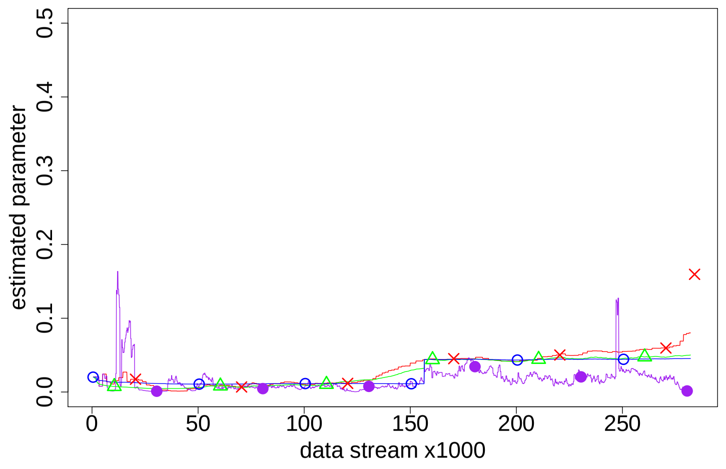

Figure (73) Variance of Saturday effect

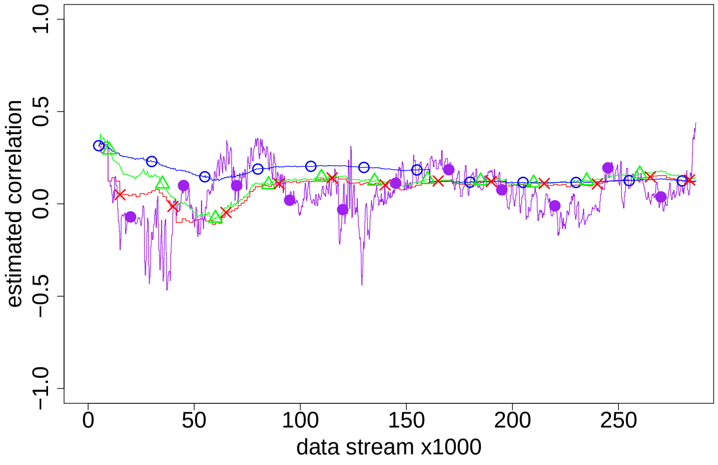

Figure (74) Correlation of the intercept and Sunday

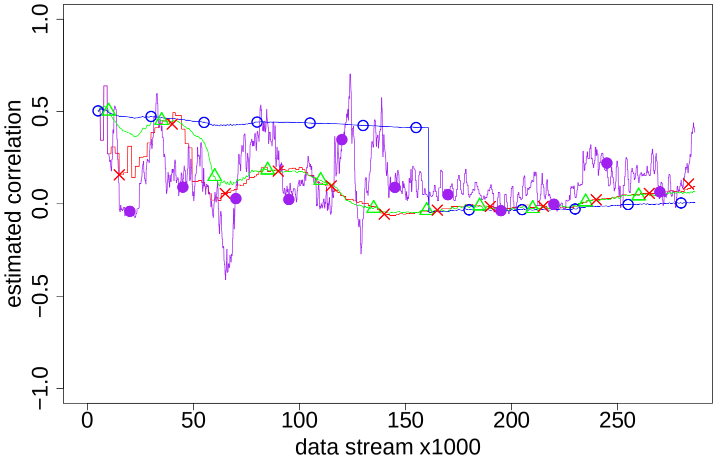

Figure (76) Correlation of the intercept and Tuesday

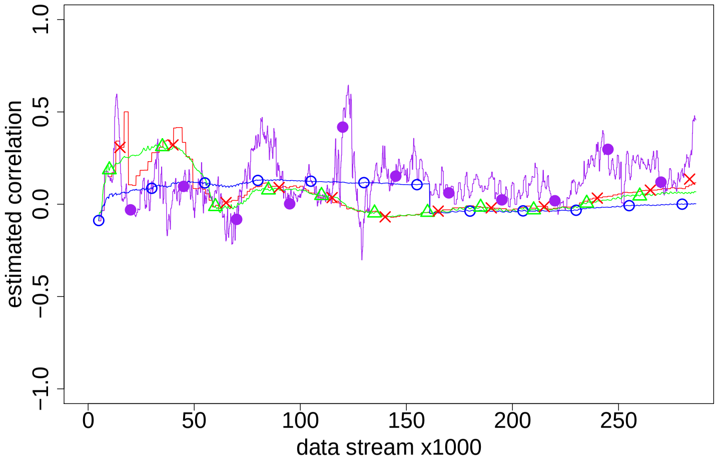

Figure (75) Correlation of the intercept and Monday

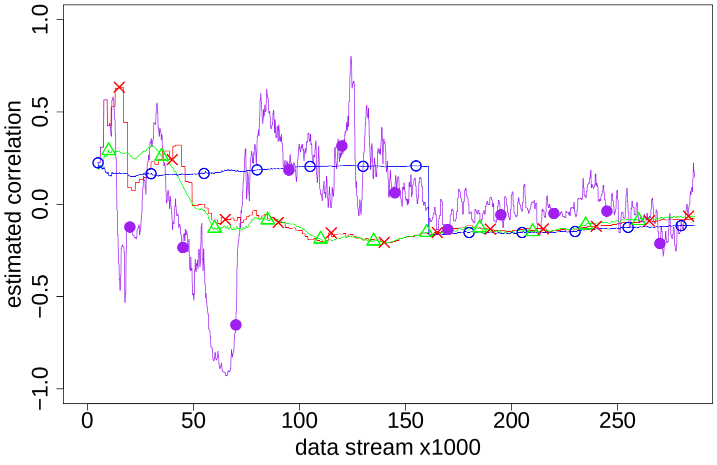

Figure (77) Correlation of the intercept and Wednesday

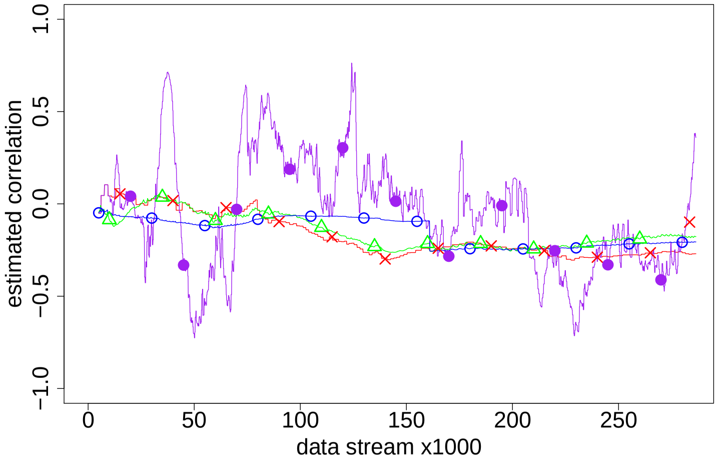

Figure (78) Correlation of the intercept and Thursday

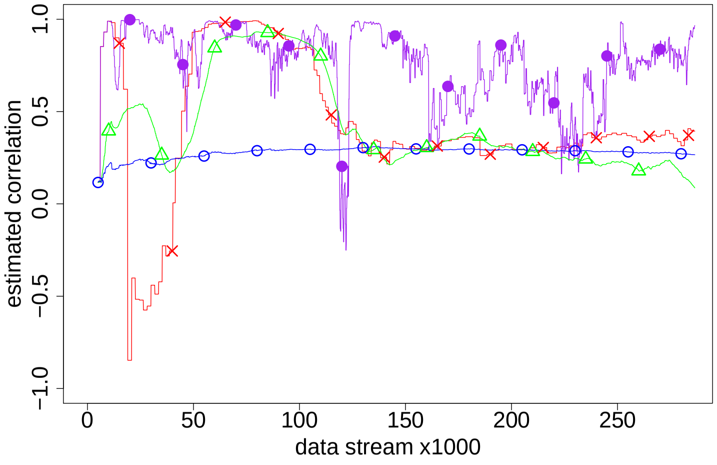

Figure (80) Correlation of Sunday and Monday

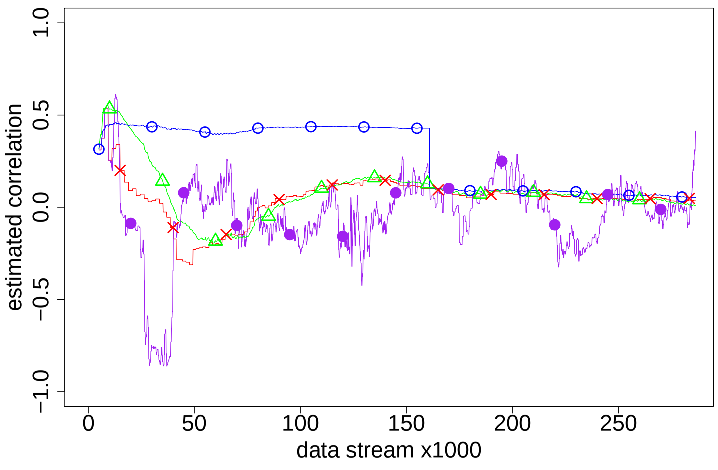

Figure (79) Correlation of the intercept and Saturday

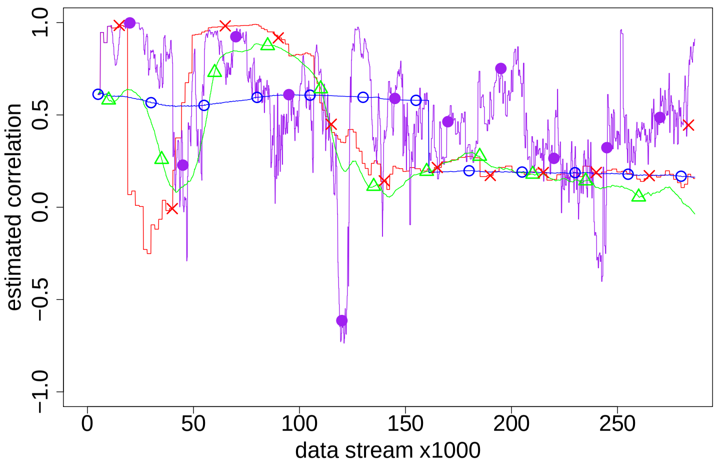

Figure (81) Correlation of Sunday and Tuesday

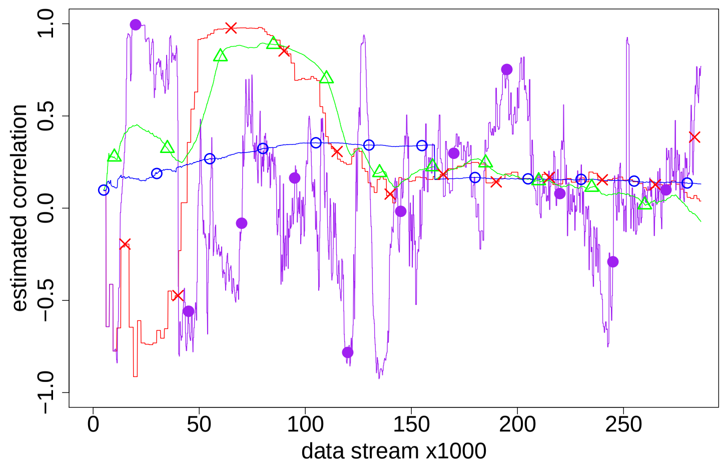

Figure (82) Correlation of Sunday and Wednesday

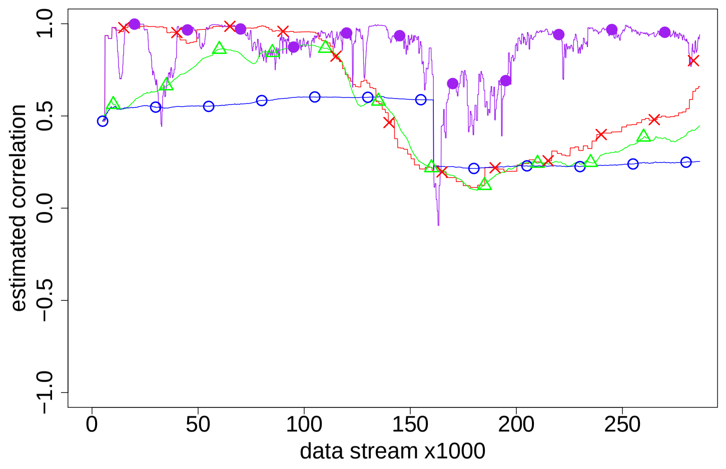

Figure (84) Correlation of Sunday and Saturday

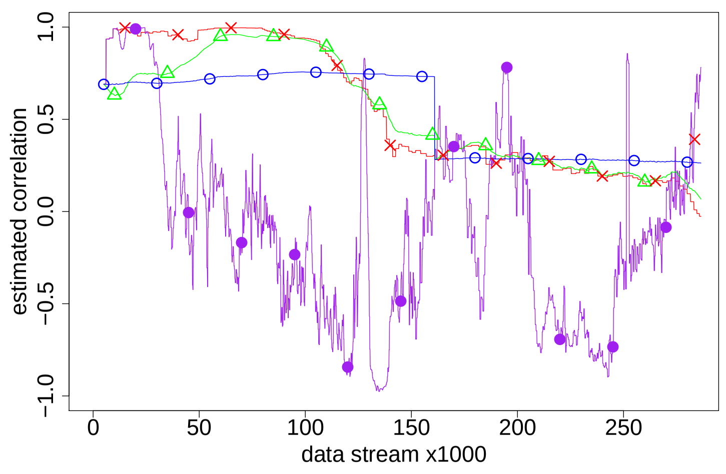

Figure (83) Correlation of Sunday and Thursday

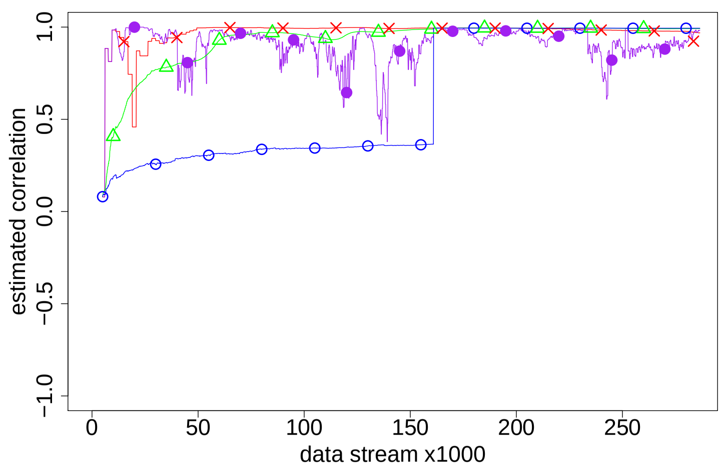

Figure (85) Correlation of Monday and Tuesday

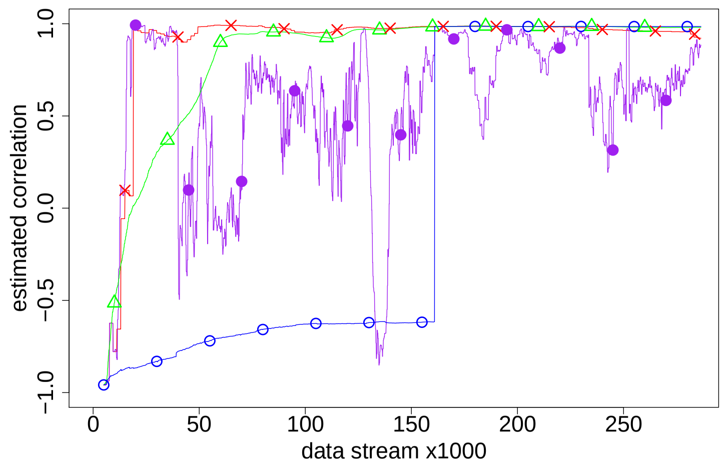

Figure (86) Correlation of Monday and Wednesday

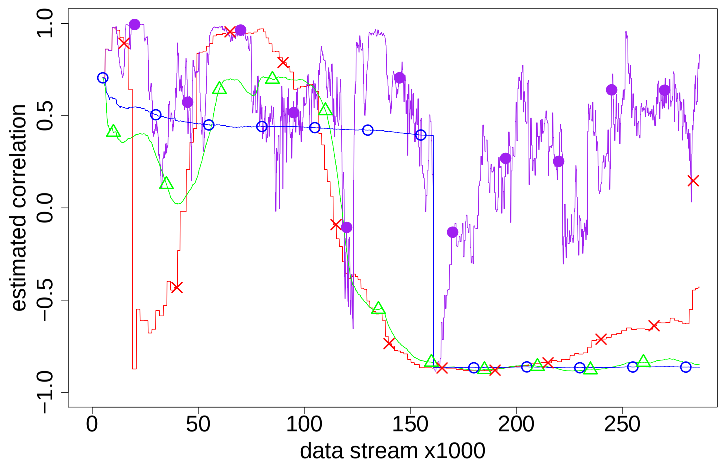

Figure (88) Correlation of Monday and Saturday

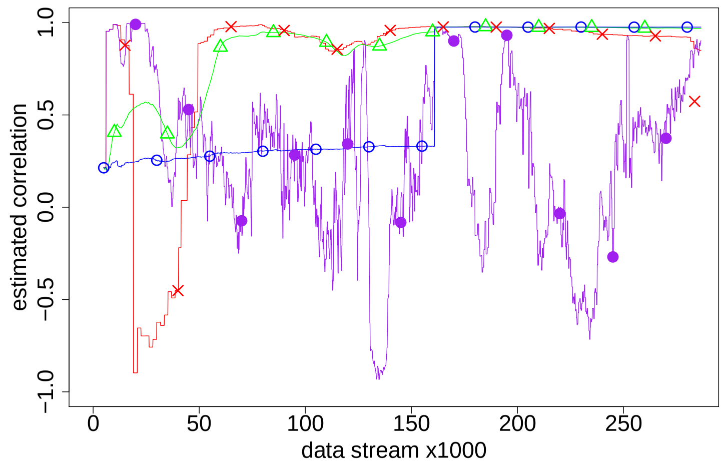

Figure (87) Correlation of Monday and Thursday

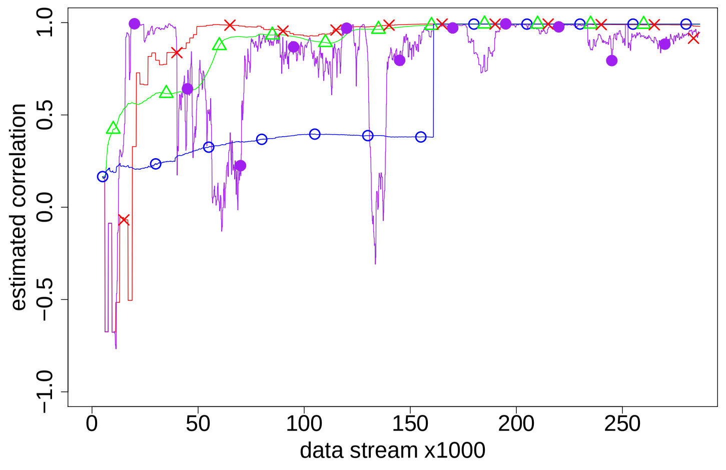

Figure (89) Correlation of Tuesday and Wednesday

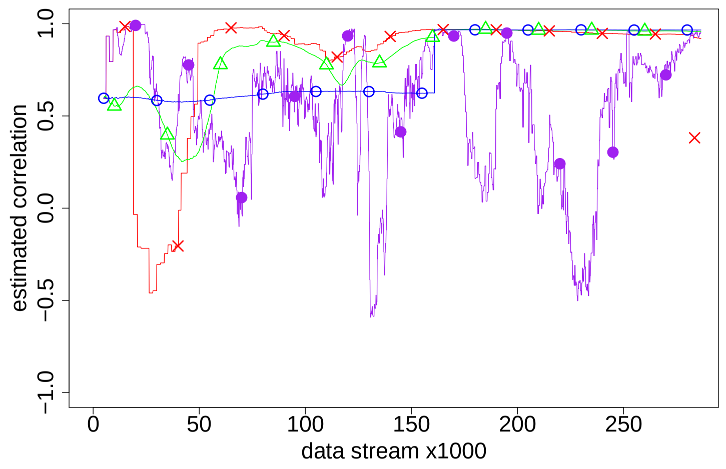

Figure (90) Correlation of Tuesday and Thursday

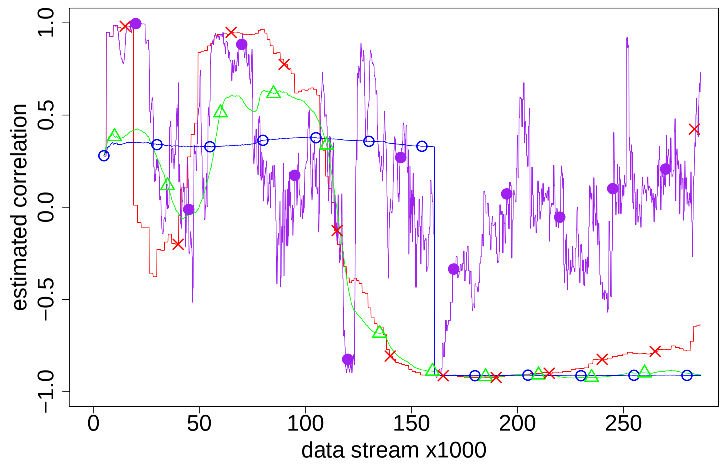

Figure (91) Correlation of Tuesday and Saturday

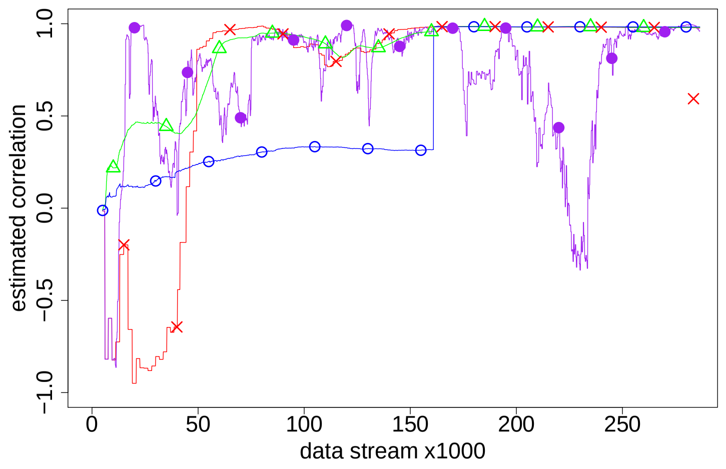

Figure (92) Correlation of Wednesday and Thursday

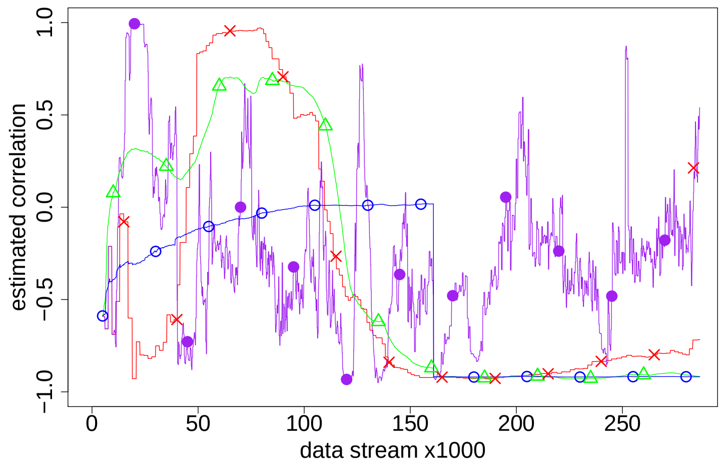

Figure (93) Correlation of Wednesday and Saturday

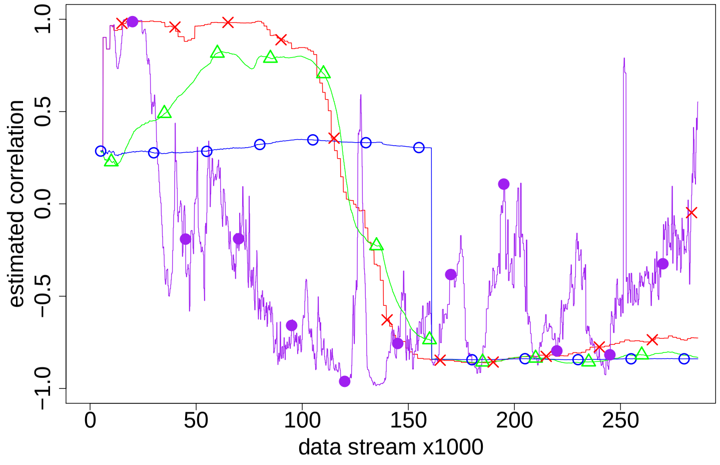

Figure (94) Correlation of Thursday and Saturday

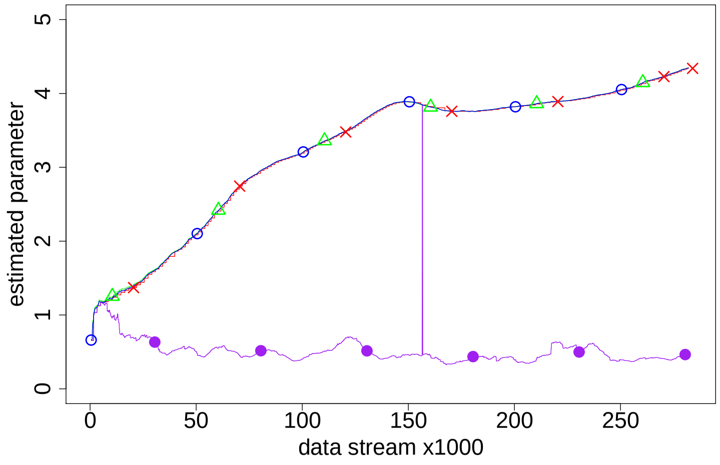

Figure (95) Residual variance

4 Simulation studies

4.1 Design

This simulation study is designed to illustrate SEMA can fit a multilevel model without using a training set and still obtain good parameter estimates. We compare the parameter estimates from SEMA with those provided by a popular R package to fit multilevel models: lmer from the lme4 package (lme4, Bates, Mächler, Bolker, and Walker, 2015). The estimation method used in the lme4 package is coined "bobyqa" (acronym for Bound Optimization BY Quadratic Approximation; Powell, 2009). This algorithm finds the best fitting parameter values by iteratively approximating the likelihood function using quadratic approximation, i.e., this algorithm does not use first or second order derivatives. We explicitly study the effect of three factors: the number of observations per individual (n_j), the number of random effects (r), and the number of level 1 covariates (lvl_1).

The number of observations n_j is an important contributor to the reliability of the estimates of $\mathbf{b}_j$: more observations per individual results in less uncertainty. Additionally, when an individual is observed more often, outdated contributions to the CDSS are more often revised than in a scenario where the number of observations is limited. Therefore, we expect SEMA will obtain accurate parameter estimates in conditions with a higher number of observations using less data points than in conditions where individuals are observed less often. The second factor, the number of random effects (r) also influences the uncertainty: the information in the data is spread out over the number of variables. We expect that more random effects will, therefore, influence SEMA such that it has to evaluate more data points to obtain good parameter estimates than in the condition where the number of random effects is small. Lastly, for the number of covariates on the first level (lvl_1), we have similar expectations for the number of random effects: more fixed effects will lead to a slower retrieval (i.e., more data points will have to enter) of the data-generating parameters because the information is spread out over more parameters. The three factors are all crossed, however, we will not let the number random effects exceed the number of covariates.

The data streams are generated with variance terms of the random effects equal to 4 and 9 in the case of $r = 2$, and in the case of $r = 7$: $\phi^2 = 4$, 9, 16, 2.25, 6.25, 12.25, and 20.25 respectively. The fixed effects are generated with the following parameter values: $\beta = 3.5, -4.5$, and -5.5 for scenarios with 3 level one fixed effects and $3.5, 4.5, -5.5, -2.5, -3.5, -4.5, 5.5$, and 6.5 for 8 level 1 fixed effects. Additionally, the residual variance, the number of level 2 variables, and the length of the data stream were fixed across the conditions (with $\sigma^2 = 25$, $\beta = 1.5$ and 2.5; and $n = 50,000$ data points). In order to illustrate that SEMA performs well, even with start values which are not ideal, the start values of all parameters were set equal to 1. Due to the computational complexity of the offline fitting procedure, the Lmer function is fitted to the data streams only every $n = 1,000$ data points instead of after each data point. Each condition was replicated 100 times.

4.2 Results

In Figure 51, the average estimated variance terms of both σ^2 and the variance of the random intercept and slope over the 100 replications are presented. On the x-axes the length of the data stream are presented, and on the y-axes the parameter estimates are presented. In the top right corner of each figure, the simulation condition is written. The gray lines represent the parameter estimates obtained using Lmer, the black lines the parameters estimates of SEMA. The Lmer function was not always able to converge in the beginning of the data stream, in these cases the gray lines are omitted from the figure. A comparison between the $n_j = 50$ and the $n_j = 10$ conditions shows that SEMA rapidly approaches Lmer's parameter estimates, especially when the number of observations for each individual is large. Furthermore, SEMA provides estimates even when Lmer is unable to converge. There is hardly any difference between including 3 level 1 predictors or 8 level 1 predictors: the top two figures and the two figures in the middle row are, given the number of observations per individual, very similar.

The bottom two figures deviate from the figures above since these conditions are, even for the Lmer algorithm, very difficult to fit. In the bottom left figure, Lmer is only able to fit the model when at least 34,000 data points are available. Even when Lmer is able to fit the model, Lmer cycled through the data thousands of times to obtain convergence. While Lmer is able to fit the model using less data in the lower right panel than in the lower left panel, still Lmer revisited the same data thousands of times to fit the model and hence took (very) long times to compute. Comparing the results of SEMA in the panel in the lower left panel with the results of SEMA in the other panels, it is clear that this lower left panel (condition: $n_j = 10, r = 7$, $\text{lvl}_1 = 8$) is, as expected, a difficult condition. Especially in the extremely challenging condition where only 10 observations per individual are available to estimate a large number of fixed and random effects, it is clear that the parameter estimates of SEMA have not yet converged, even at the end of the data stream. However, when there is more information available per individual ($n_j = 50$), SEMA performs much better (lower right panel) than in the condition with $n_j = 10$.

Next, we present three tables with the parameter estimates averaged over the replications, the standard deviation over the replications and the 95% interval based on the empirical distribution of results of the simulation study (percentiles). In Table 1, we present the estimates of two of the fixed effects estimated by SEMA and Lmer at two points in the data stream. Since the (qualitative) behavior is similar across all fixed effects, we choose to present only these two. Across conditions, we can conclude from Table 1 that the fixed effects are estimated well by both Lmer and SEMA, with the mere difference that the SD's of SEMA are slightly larger (however, SEMA is magnitudes faster).

The estimates of the variance of the random intercept and one of the random slopes ($\phi^2 = 4$, and 9) are presented in Table 2. Clearly, these variance terms are more difficult to estimate for SEMA than the fixed effects. While Lmer retrieves the true values as soon as it is able to fit the model, SEMA needs more data to obtain good estimates of the variance terms. Finally, in Table 3, the estimates of the residual variance ($\sigma^2 = 25$) are presented. The same conditions which showed a large SD for $\hat{\phi}$, have a large SD for the estimates of $\hat{\sigma}^2$. Overall, SEMA and Lmer produce very similar estimates of σ^2, although the condition of $n_j = 10, r = 7$, $\text{lvl}_1 = 8$ with start values equal to 1, remains difficult for SEMA even when 50,000 data points have entered.

5 Ordering of data points

Here, we illustrate that the ordering of the data points does not influence the end results of the parameter estimates. To do so, we generated a data stream consisting of $n = 1,000,000$ and $J = 20,000$ and examine the effects of reordering the data during the stream. The data were generated using a random intercept model. The values of the fixed effects are: 100.0, 0.1, 0.5, 0.9, 1.3, 1.7, 2.1, 2.5, 2.9, 3.3, 3.7, 4.1, 4.5, 4.9, and 5.3. The variance of the random intercept is 50, and the residual variance was equal to 5. The data set was reordered 11 times, using the correlation between the b_j's and the moment of entering in the data stream. The correlation is varied from nearly perfectly positive to nearly perfectly negative. Even though there are differences between the different orderings, overall the parameter estimates are very comparable across orderings.

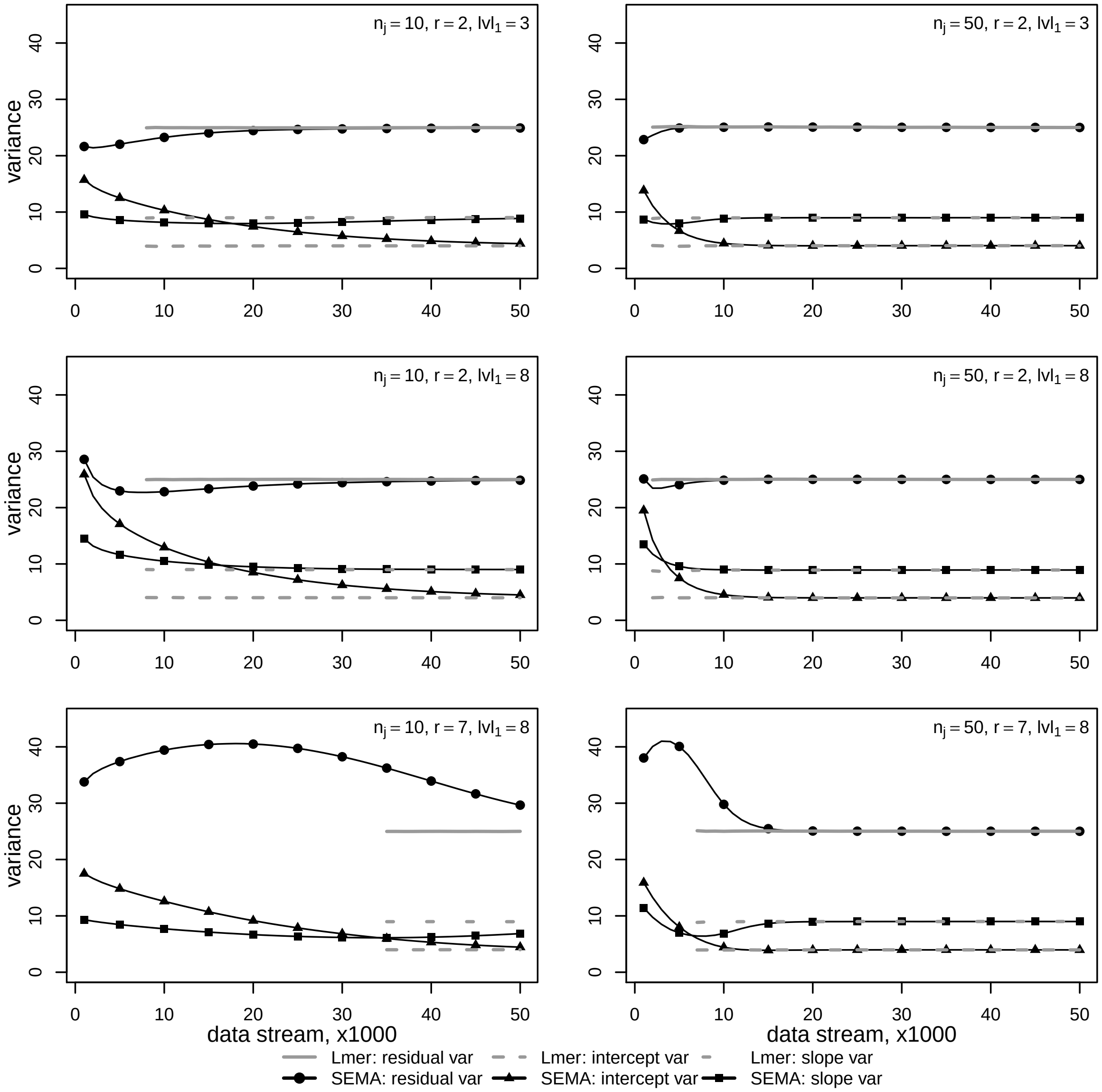

Figure (96) Estimated residual variance and random intercept and random slope. Note that for two graphs on the bottom, not all variance terms are included in the graph.

Table (1) The variability of the estimates of β, across replications of Lmer and SEMA

					Lmer				SEMA			
n_j	r	lvl_1	β	$n_{\times 1000}$	$\hat{\beta}$	SD	2.5%	97.5%	$\hat{\beta}$	SD	2.5%	97.5%
10	2	3	1.5	25	1.508	0.047	1.415	1.602	1.511	0.066	1.411	1.634
				50	1.505	0.037	1.438	1.567	1.507	0.039	1.440	1.591
			-5.5	25	-5.500	0.034	-5.555	-5.447	-5.499	0.035	-5.559	-5.445
				50	-5.499	0.024	-5.542	-5.452	-5.499	0.024	-5.542	-5.452
50	2	3	1.5	25	1.490	0.069	1.331	1.604	1.490	0.067	1.341	1.601
				50	1.491	0.065	1.339	1.591	1.491	0.065	1.343	1.587
			-5.5	25	-5.497	0.032	-5.554	-5.437	-5.497	0.032	-5.554	-5.437
				50	-5.497	0.021	-5.535	-5.461	-5.497	0.021	-5.535	-5.461
10	2	8	1.5	25	1.500	0.041	1.415	1.570	1.511	0.169	1.405	1.583
				50	1.502	0.035	1.432	1.568	1.506	0.061	1.426	1.568
			-5.5	25	-5.497	0.038	-5.566	-5.424	-5.496	0.038	-5.564	-5.420
				50	-5.496	0.026	-5.544	-5.444	-5.496	0.026	-5.544	-5.445
50	2	8	1.5	25	1.501	0.073	1.361	1.641	1.500	0.073	1.358	1.639
				50	1.500	0.071	1.374	1.632	1.501	0.071	1.377	1.637
			-5.5	25	-5.495	0.035	-5.554	-5.430	-5.495	0.035	-5.554	-5.430
				50	-5.497	0.023	-5.542	-5.459	-5.497	0.023	-5.542	-5.459
10	7	8	1.5	25	–	–	–	–	1.469	0.238	0.962	1.972
				50	1.502	0.039	1.419	1.576	1.494	0.080	1.344	1.657
			-5.5	25	–	–	–	–	-5.175	0.337	-5.563	-4.305
				50	-5.498	0.044	-5.574	-5.411	-5.401	0.127	-5.558	-5.093
50	7	8	1.5	25	1.489	0.071	1.371	1.632	1.489	0.070	1.374	1.626
				50	1.492	0.073	1.374	1.633	1.491	0.073	1.367	1.626
			-5.5	25	-5.490	0.083	-5.653	-5.337	-5.475	0.088	-5.630	-5.309
				50	-5.487	0.080	-5.644	-5.331	-5.486	0.080	-5.648	-5.325

Table (2) The variability of the estimates of ϕ, across replications of Lmer and SEMA (the "–" indicate that Lmer failed to converge in this condition).

					Lmer				SEMA			
n_j	r	lvl_1	ϕ	$n_{\times 1000}$	$\hat{\phi}^2$	SD	2.5%	97.5%	$\hat{\tau}^2$	SD	2.5%	97.5%
10	2	3	4	25	4.000	0.211	3.575	4.335	6.468	0.706	4.649	7.379
				50	4.001	0.137	3.738	4.258	4.396	0.215	3.989	4.664
			9	25	9.007	0.374	8.399	9.807	8.057	1.979	3.963	11.041
				50	9.018	0.257	8.560	9.580	8.860	0.403	7.875	9.422
50	2	3	4	25	4.024	0.220	3.636	4.466	4.022	0.219	3.631	4.464
				50	4.032	0.205	3.684	4.439	4.032	0.205	3.687	4.441
			9	25	8.987	0.481	8.220	10.148	8.987	0.480	8.230	10.146
				50	8.990	0.444	8.294	9.930	8.990	0.443	8.296	9.930
10	2	8	4	25	4.018	0.201	3.588	4.451	7.196	0.828	6.521	8.209
				50	3.999	0.140	3.774	4.283	4.507	0.167	4.276	4.794
			9	25	8.981	0.315	8.391	9.568	9.257	1.775	5.356	11.121
				50	8.994	0.224	8.586	9.388	9.013	0.306	8.486	9.528
50	2	8	4	25	3.982	0.251	3.448	4.454	3.982	0.250	3.441	4.452
				50	3.975	0.223	3.562	4.356	3.976	0.223	3.560	4.355
			9	25	8.921	0.474	7.913	9.762	8.922	0.475	7.917	9.773
				50	8.928	0.432	8.101	9.646	8.928	0.432	8.100	9.647
10	7	8	4	25	–	–	–	–	7.870	1.398	5.723	10.464
				50	4.000	0.156	3.714	4.288	4.449	0.451	3.667	5.326
			9	25	–	–	–	–	6.358	2.529	2.434	12.214
				50	8.971	0.279	8.465	9.453	6.855	1.205	4.656	9.175
50	7	8	4	25	3.971	0.236	3.546	4.436	3.965	0.238	3.536	4.439
				50	3.959	0.197	3.566	4.325	3.960	0.197	3.567	4.323
			9	25	8.999	0.441	8.165	9.857	8.996	0.442	8.164	9.873
				50	8.999	0.417	8.310	9.745	8.999	0.417	8.309	9.746

Table (3) The variability of the estimates of σ^2 across replications of Lmer and SEMA

				Lmer				SEMA			
n_j	r	lvl_1	$n_{\times 1000}$	$\hat{\sigma}^2$	SD	2.5%	97.5%	$\hat{\sigma}^2$	SD	2.5%	97.5%
10	2	3	25	24.936	0.254	24.548	25.551	24.659	0.911	23.657	26.977
			50	24.985	0.178	24.627	25.308	24.921	0.195	24.568	25.314
50	2	3	25	25.068	0.206	24.727	25.534	25.069	0.206	24.726	25.528
			50	25.012	0.167	24.679	25.348	25.012	0.167	24.679	25.349
10	2	8	25	25.019	0.288	24.470	25.531	24.202	0.524	23.429	25.512
			50	24.985	0.179	24.619	25.350	24.865	0.175	24.494	25.269
50	2	8	25	25.020	0.239	24.566	25.449	25.021	0.240	24.565	25.452
			50	25.006	0.155	24.726	25.293	25.006	0.155	24.725	25.293
10	7	8	25	–	–	–	–	39.729	5.599	30.297	50.673
			50	25.001	0.217	24.635	25.397	29.656	2.173	26.032	34.261
50	7	8	25	25.027	0.238	24.622	25.498	25.030	0.236	24.630	25.496
			50	25.016	0.159	24.737	25.345	25.016	0.159	24.739	25.345

Table (4) Parameter estimates of 11 different orderings of a single data set of $n = 1,000,000$.

	Correlation b_j and ordering of data stream											
	0.9773	0.6913	0.4849	0.3053	0.1773	0.0000	0.1789	-0.1782	-0.3026	-0.4859	-0.6911	-0.9773
int	97.4267	95.5715	98.0962	99.0922	99.2939	99.7997	99.0662	101.1900	101.3517	102.2185	104.38	103.5271
$lvl_{1.x1}$	0.1024	0.1023	0.1023	0.1023	0.1023	0.1023	0.1256	0.1023	0.1023	0.1023	0.1023	0.1022
$lvl_{1.x2}$	0.5013	0.5014	0.5014	0.5014	0.5014	0.5014	0.5368	0.5014	0.5014	0.5014	0.5014	0.5014
$lvl_{1.x3}$	0.8983	0.8984	0.8984	0.8984	0.8984	0.8984	0.8835	0.8984	0.8984	0.8984	0.8985	0.8985
$lvl_{1.x4}$	1.2988	1.2988	1.2988	1.2988	1.2988	1.2988	1.3062	1.2988	1.2988	1.2988	1.2988	1.2988
$lvl_{1.x5}$	1.6982	1.6982	1.6982	1.6982	1.6982	1.6982	1.6946	1.6983	1.6982	1.6983	1.6982	1.6982
$lvl_{1.D1a}$	2.0998	2.0998	2.0998	2.0998	2.0999	2.0998	2.0697	2.0999	2.0998	2.0998	2.0998	2.0997
$lvl_{1.D1b}$	2.5023	2.5024	2.5024	2.5025	2.5025	2.5025	2.4938	2.5026	2.5025	2.5025	2.5026	2.5026
$lvl_{1.D1c}$	2.9037	2.9037	2.9038	2.9038	2.9038	2.9038	2.8815	2.9038	2.9038	2.9038	2.9039	2.9038
$lvl_{2.x1}$	3.2935	3.3461	3.3113	3.0596	3.0139	3.2786	3.5727	1.0695	3.3196	3.2782	3.3465	3.5826
$lvl_{2.x2}$	3.3154	3.7240	3.7462	3.9605	3.9796	3.7720	3.6695	3.1365	3.7420	3.6376	3.6287	3.4164
$lvl_{2.x3}$	4.2986	4.1372	4.0777	4.1825	4.5108	4.0821	4.0483	5.1336	4.0725	4.1986	4.2793	4.1971
$lvl_{2.D2a}$	3.9016	4.4241	4.2476	4.4717	5.2626	4.5242	4.5962	5.3135	4.5014	4.0305	4.8190	4.3363
$lvl_{2.D3a}$	4.1729	4.7281	5.1091	4.8984	5.4055	5.3849	5.3415	4.6942	4.6960	4.8131	4.6376	5.1934
$lvl_{2.D3b}$	4.6200	5.0752	5.3473	5.3129	4.6014	5.6063	6.0743	9.6533	5.0536	5.3101	4.9207	5.5336
ϕ^2	61.4059	71.0848	53.6706	50.9233	50.8272	50.0100	40.8477	69.8272	51.3685	53.7577	68.6703	63.1411
σ^2	5.0003	5.0003	5.0003	5.0003	5.0003	5.0003	4.9718	5.0002	5.0003	5.0002	5.0003	5.0002

References

[1] Bates, Douglas, Mächler, Martin, Bolker, Ben & Walker, Steve (2015). Fitting Linear Mixed-Effects Models Using lme4. *Journal of Statistical Software*, *67*(1), 1–48, doi = 10.18637/jss.v067.i01.

[2] Kooreman, Peter & Scherpenzeel, Annette. (2014). High frequency body mass measurement, feedback and health behaviors. *Economics and Human Biology*, *14*, 141–153.

[3] McLachlan, Geoffrey, & Peel, David. (2000). *Finite Mixture Models*. New York, USA: Wiley series in probability and statistics.

[4] Powell, Michael J. D. (2009). *The BOBYQA algorithm for bound constrained optimization without derivatives*, Report No. DAMTP 2009/NA06, Centre for Mathematical Sciences, University of Cambridge, UK. Retrieved: March 30, 2017.
